# Global Analysis of Posttranslational Protein Arginylation

**DOI:** 10.1371/journal.pbio.0050258

**Published:** 2007-09-25

**Authors:** Catherine C. L Wong, Tao Xu, Reena Rai, Aaron O Bailey, John R Yates, Yuri I Wolf, Henry Zebroski, Anna Kashina

**Affiliations:** 1 The Scripps Research Institute, LaJolla, California, United States of America; 2 Department of Animal Biology, University of Pennsylvania, Philadelphia, Pennsylvania, United States of America; 3 National Center for Biotechnology Information, National Library of Medicine, National Institutes of Health, Bethesda, Maryland, United States of America; 4 Rockefeller University, New York, NewYork, United States of America; Stanford University, United States of America

## Abstract

Posttranslational arginylation is critical for embryogenesis, cardiovascular development, and angiogenesis, but its molecular effects and the identity of proteins arginylated in vivo are largely unknown. Here we report a global analysis of this modification on the protein level and identification of 43 proteins arginylated in vivo on highly specific sites. Our data demonstrate that unlike previously believed, arginylation can occur on any N-terminally exposed residue likely defined by a structural recognition motif on the protein surface, and that it preferentially affects a number of physiological systems, including cytoskeleton and primary metabolic pathways. The results of our study suggest that protein arginylation is a general mechanism for regulation of protein structure and function and outline the potential role of protein arginylation in cell metabolism and embryonic development.

## Author Summary

A common cellular mechanism for the regulation of proteins, once they have been translated from mRNA, is the addition and removal of chemical groups via enzymatic reactions. The posttranslational addition of arginyl groups is critical for the embryonic development and survival of an organism, but the molecular effects and the identity of proteins arginylated in vivo are largely unknown. We developed a technique to screen large numbers of proteins for this modification and identified 43 proteins arginylated in vivo on highly specific sites. Arginylation can occur on any exposed residue at the N-terminus of a protein and appears to require a specific structural recognition motif on the protein surface. It preferentially affects a number of physiological systems, including cytoskeleton and primary metabolic pathways and seems to be a general mechanism for regulation of protein structure and function. Our data provide insights into the previously unknown arginylation-dependent mechanisms of the regulation of embryonic development.

## Abstract 3

A comprehensive study indicates that protein arginylation may be a general mechanism for regulation of protein structure and function, similar to other major posttranslational modifications.

## Introduction

Protein arginylation is an enigmatic posttranslational modification mediated by arginyl-tRNA-protein transferase (ATE1) that transfers arginine (Arg) from tRNA onto proteins [1]. Until recently it had been believed that arginylation can only occur on the exposed N-terminus of aspartic acid (Asp), glutamic acid (Glu), or cysteine (Cys); however, a single case of the addition of Arg onto the side chain of Glu has been recently identified in vivo [2].

Despite the fact that arginylation was originally discovered more than 40 y ago [3,4], its biological functions are still poorly understood. ATE1 enzyme is highly conserved in evolution. Eukaryotic species, from yeast to human, appear to contain only one arginyltransferase gene and no other known enzymes with a similar activity. ATE1 is nonessential in yeast, but required for embryogenesis in mammals [5]. Mouse *Ate1* knockout results in embryonic lethality and severe defects in cardiovascular development and angiogenesis [5]. It has been believed that the molecular function of arginylation is to induce degradation of the target protein substrates by the ubiquitin-dependent N-end rule pathway [6]. Indeed, arginylation by ATE1 induces rapid degradation of experimentally constructed test proteins in yeast [6], and the half-life of RGS family proteins in mammals has been shown to decrease upon arginylation [7]. Recently, however, it has been found that arginylation regulates structure and intracellular assembly of beta actin in motile cells without affecting its short-term metabolic stability [8], suggesting that the function of arginylation in vivo may be more complex.

The most direct way to understand the function of protein arginylation is by identifying the in vivo substrates of ATE1; however, such identification is difficult for the following reasons. First, arginylation by ATE1 is believed to involve Arg-tRNA that is also utilized during protein synthesis, and to result in the formation of a normal peptide bond. As a result, arginylated proteins are difficult to distinguish in biochemical tests or to label differentially from other intracellular proteins. Second, arginylation is believed to occur only on the N-terminally exposed residues other than methionine (Met) 1 found in all proteins immediately after translation initiation; therefore, arginylation requires a preceding posttranslational modification either by proteolysis or by aminopeptidation—modifications whose targets are themselves poorly characterized. Finally, the situation is further complicated by the cases where arginylation results in decreased metabolic stability, making at least some arginylated proteins less abundant in vivo. Thus, despite numerous evidence of protein arginylation in vivo [9–14] and multiple attempts to identify arginylated proteins, no systematic progress has been made beyond identification of several targets [7,15–19].

In this study we utilize the arginyltransferase knockout (*Ate1* ^−/−^) mouse model [5] and use two-dimensional (2D) gel fractionation, metabolic labeling by Arg, immunoaffinity chromatography, and mass spectrometry (MS) to estimate the entire complexity of arginylation, identify key proteins arginylated in vivo, and gain insights into the biological functions of arginylation as a posttranslational modification. Our analysis reveals that a vast number of proteins, on the order of hundreds, are potentially arginylated in vivo. While some of these proteins are possibly metabolically destabilized upon arginylation, a much larger number of proteins are not. We find that in addition to N-terminal Asp, Glu, and Cys, proteins can be arginylated on other N-terminally exposed amino acid residues, including one identified case of arginylation on N-terminal Met. We further find that proteins arginylated in vivo fall into several distinct functional groups with roles in cytoskeleton, transcriptional regulation, and general metabolic pathways. These proteins are arginylated in vivo on a limited number of sites predicted to affect their key functional properties.

Our results suggest that protein arginylation plays a general regulatory function in vivo by modulating the structure of proteins and affecting their intracellular functions.

## Results

### Protein Arginylation and Proteasome-Dependent Protein Degradation

**Figure 1:**
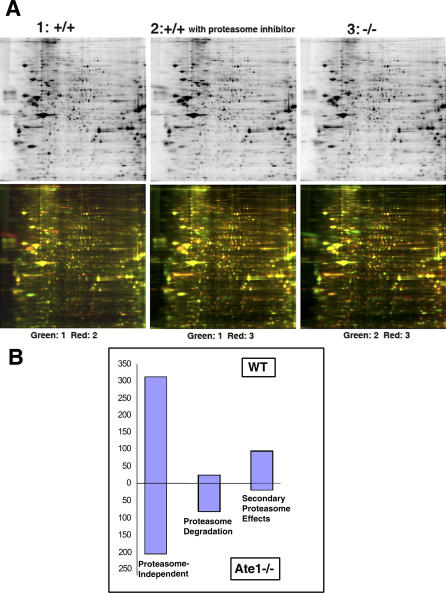
2D Gel Comparison of the Effects of Arginylation and Proteasome Degradation (A) Top row: individual images of 10% 2D gels (pH range 3.5–10 increases left to right) of whole cell extracts from wild-type (+/+, WT) cells labeled with Cy2 (1, left), WT cells treated with proteasome inhibitor labeled with Cy3 (2, middle), and arginylation-free *Ate1* ^−/−^ cells labeled with Cy5 (3, right). Bottom row: pairwise comparison of the gels shown on top in different fluorescence channels. Colors were altered to red and green to allow better visual comparison. (B) Quantification of the number of spots up- or down-regulated in response to each treatment. See also Table S4. Proteins identified in the most abundant spots from Coomassie-stained gels obtained under similar conditions are listed in Tables S1 (reflecting the comparison between gels 1 and 3) and S2 (reflecting the comparison between gels 1 and 2). See explanations in the text.

To estimate the complexity of effects of arginylation on the protein level, we performed a comparison of all proteins up- and down-regulated in response to arginylation in a proteasome-degradation-dependent and -independent way. To do that, we fractionated whole cell extracts from wild-type immortalized fibroblasts in culture (Figure 1A, panel 1), wild-type fibroblasts treated with proteasome inhibitor MG-132 (panel 2), and *Ate1* ^−/−^ fibroblasts derived from an *Ate1* knockout mouse [5] (panel 3) by 2D gel electrophoresis, and compared protein composition in the extracts (Figure 1). Such analysis is estimated to resolve approximately 2,500 protein spots. To enable accurate comparison, cell extracts were equalized by protein concentration, labeled with Cy2 (Figure 1A, panel 1), Cy3 (panel 2), and Cy5 (panel 3), ran on the same gel, and visualized in three fluorescence channels (pictures in individual channels are shown in black and white in the top set of images in Figure 1A, and their pairwise color comparisons are shown in the bottom set of images).

A total of 1,030 different protein spots were found altered between the three gels (Figure 1; Table S4). Of those, 297 spots were up- or down-regulated in response to the treatment with proteasome inhibitor but showed no dependence on the absence or presence of ATE1, being of similar level in wild-type and *Ate1* ^−/−^ extracts. The remaining 733 protein spots were altered in an *Ate1*-dependent way. These spots were subjected to further analysis.

First, we estimated the number of spots whose arginylation is likely to result in their degradation by the N-end rule pathway. We reasoned that such spots would show increased levels in *Ate1* ^−/−^ compared to wild-type cells (indicating that arginylation in wild-type cells results in their destabilization and degradation), and that the same spots would be similarly increased in wild-type cells treated with the proteasome inhibitor (indicating that their degradation in wild-type cells is proteasome-dependent). Surprisingly, out of the 733 spots regulated by ATE1 only 83 showed such behavior.

The “N-end rule-independent” spots showed the following patterns of regulation. A total of 313 spots were up-regulated in wild-type cells compared to *Ate1* ^−/−^ cells, but showed no response to proteasome inhibitor treatment. A total of 110 spots were up-regulated in wild-type cells compared to *Ate1* ^−/−^ cells, while showing uncorrelated up- or down-regulation in response to proteasome inhibitor, suggesting that regulation of these proteins by arginylation and proteasome degradation are independent of each other. A total of 208 spots were up-regulated in *Ate1* ^−/−^ cells compared to wild-type cells, but showed no dependence on proteasome inhibition. A total of 19 spots were up-regulated in *Ate1* ^−/−^ cells compared to wild-type cells, and showed various arginylation-independent responses to proteasome inhibition. These results suggest that while arginylation apparently regulates the levels of many proteins, this regulation generally has no direct correlation with their degradation by the proteasome, and point to other functions of protein regulation by arginylation or downstream effects of knocking out *Ate1*.

To identify proteins whose levels are regulated by either arginylation or proteasome inhibition, we performed a computerized comparison of protein spot composition on individually run Coomassie-stained 2D gels of wild-type, MG-132-treated, and *Ate1* ^−/−^ cell extracts, excised spots clearly visible by Coomassie staining that were up- or down-regulated in response to *Ate1* knockout or up-regulated in response to proteasome inhibition, and performed their mass spectrometric identification. The names and accession numbers of those proteins are listed in Tables S1 and S2. We would like to point out that, given the complexity of the samples, the presence of multiple proteins in some spots, and the limited comparative abundance of some proteins, this list does not cover, or definitively reflect, the entire complexity of proteins regulated by arginylation. Limited abundance and high complexity of the spots also precluded us from accurate determination of the behavior of the specific identified proteins in response to proteasome inhibition and *Ate1* knockout.

### Posttranslational Arg Incorporation into Proteins in Cultured Cells

**Figure 2:**
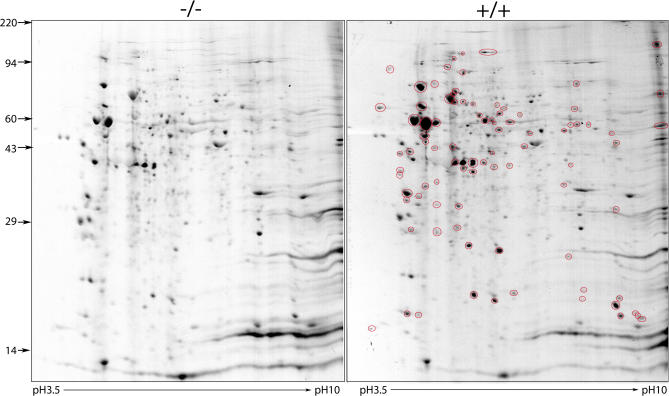
Posttranslational Incorporation of Arg into Proteins in Cultured Cells Autoradiographs of wild-type (+/+) and *Ate1* ^−/−^ (−/−) whole cell extracts treated with cycloheximide in medium containing ^14^C-Arg, fractionated on 2D gels. Spots circled in red in the wild-type gel show increase in Arg incorporation above that in the *Ate1* ^−/−^ cells, suggesting that Arg in these spots is incorporated posttranslationally. Proteins identified in the most abundant of the up-regulated spots are listed in Table S3.

To identify proteins arginylated in vivo, we first applied the previously developed assays of posttranslational incorporation of radioactively labeled Arg into cells in culture in the presence of protein synthesis inhibitors [9], and analyzed the total homogenates from such cells by 2D gel autoradiography (Figure 2). To distinguish between posttranslationally incorporated Arg and Arg incorporated into proteins during the residual protein synthesis, we performed similar treatment of arginylation-deficient cells derived from the *Ate1* ^−/−^ mouse (see above). We concluded that the spots showing increased Arg incorporation in wild-type compared to *Ate1* ^−/−^ cells under these conditions represent proteins with posttranslationally incorporated Arg.

Protein abundance in the majority of these spots was low, which precluded accurate identification of proteins in these spots. However, we were able to excise 52 spots from duplicate nonradioactive gels whose positions corresponded to those circled in red in the wild-type gel shown in Figure 2. Proteins in these spots were identified by MS (Table S3) and represent potential arginylation targets in vivo. It should be noted that the majority of these proteins were found at their expected molecular weights, indicating that if they were indeed arginylated, their arginylation was not accompanied by degradatory proteolysis.

Since many of the excised spots contained more than one protein, we could not tell definitively which of those proteins were targets for arginylation. In addition, a large number of the spots were not abundant enough for reliable MS identification. Therefore, we developed a definitive approach to the mass target identification in vivo.

### Identification of Proteins Arginylated In Vivo

**Figure 3:**
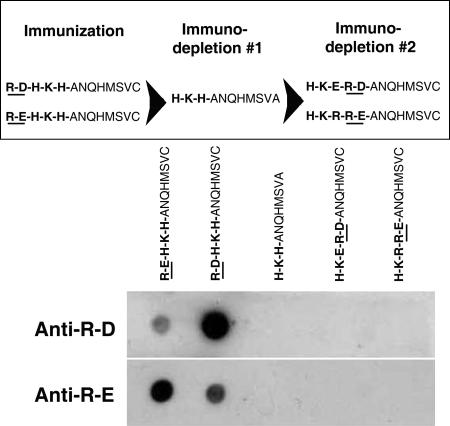
Generation of the Antibodies to N-Terminal Arg Top: antibody generation strategy showing the sequences of the peptides used for antibody production and immunodepletion to ensure antibody specificity for Arg in the N-terminal position. Bottom: dot blot of the affinity-purified antibodies to N-terminal Arg–Asp and Arg–Glu sequences against the peptides listed on the top.

To isolate and definitively identify proteins arginylated in vivo, we raised two antibodies against N-terminally arginylated proteins. As antigens we used peptides with N-terminal Arg–Asp– and Arg–Glu– sequences (Figure 3, top), which are the major known residues to be arginylated in vivo, and incorporated these residues into a predictably highly immunogenic epitope (identified in bold in the leftmost peptides in Figure 3, top), followed by an amino acid stretch with predicted low immunogenicity. Rabbit antiserum raised against these peptides was first affinity purified on the corresponding peptides, and then immunodepleted against similar peptides without the N-terminal Arg–Asp– and Arg–Glu–, followed by immunodepletion against “scrambled” peptides, i.e., peptides containing an epitope similar to that of the original peptide but with the Arg–Asp– or Arg–Glu– in the internal position. The resulting antibodies showed extremely high specificity for the N-terminally arginylated sequences without a strong specificity for the residue in the second position (Figure 3, bottom). A similar attempt was made with a peptide containing N-terminal Arg–Cys– sequence; however, the resulting antibodies also recognized the “scrambled” Arg–Cys– peptide, and thus were omitted from our analysis.

We next covalently coupled the Arg–Asp– and Arg–Glu– antibodies to Aminolink agarose (Pierce) and performed immunoaffinity chromatography of whole cell extracts from several wild-type mouse tissues, including fibroblasts, heart, lung, liver, kidney, spleen, and brain; in addition, extracts from whole embryos collected at embryonic day 12 were used in the later parts of the analysis. Proteins bound to the columns were eluted with glycine (pH 2.7), digested with a set of proteases (varying combinations of trypsin, subtilisin, elastase, and endopeptidase Lys-C) to achieve higher sequence coverage, and analyzed by MS. We found approximately 300 proteins in the column eluates from two columns with peptide antibodies, shown in Figure 3.

To identify which of these proteins were arginylated, tandem MS (MS/MS) database searching was used to both identify peptide amino acid sequences and to determine N-terminal differential modification with Arg [20,21]. Searches yielded a large number of peptides within the initial list of 300 proteins identified that had an additional Arg (a mass of 156.1011). To eliminate potential false positives produced by modifications other than arginylation that could result in a similar mass change, we used stringent criteria for picking out the putative arginylated peptides (see Materials and Methods). We expect that by using these criteria a number of true targets were sacrificed in favor of stringency, and thus the list of arginylated proteins reported in this study is by no means exhaustive.

The initial list of proteins contained 104 stringently picked targets; however, several ambiguities were identified during the analysis. First, peptides with N-terminally adjacent Arg in the original protein sequence were discarded, since we had no way to tell that these peptides were produced by posttranslational arginylation and not proteolysis. Second, peptides with N-terminally adjacent valine (Val)–glycine (Gly) or Gly–Val sequence or an N-terminal Val, isoleucin (Ile), leucine (Leu), or Asp residue were separated for further verification, since the masses of Val + Gly (occurring naturally in the peptide or produced by glycylation of Val due to the presence of high concentration of Gly in the buffer), carbamylated Ile or Leu (modified by urea used during sample preparation), and acetylated Asp (occurring naturally or during trichloroacetic acid precipitation of the samples) are very close to the mass of Arg. To eliminate these ambiguities, standard peptides of arginylated and non-arginylated versions of these residues were produced and analyzed by MS under similar conditions to compare to the experimentally identified targets (See Dataset S2 for the standard peptide spectra of the Val–Gly– versus Arg– case and [8] for the acetylated versus arginylated spectra). Some of the analysis was also repeated on the freshly prepared antibody columns using high-salt elution conditions (3.5 M MgCl_2_) instead of Gly, and further preparation of these samples was done without urea.

**Figure 4:**
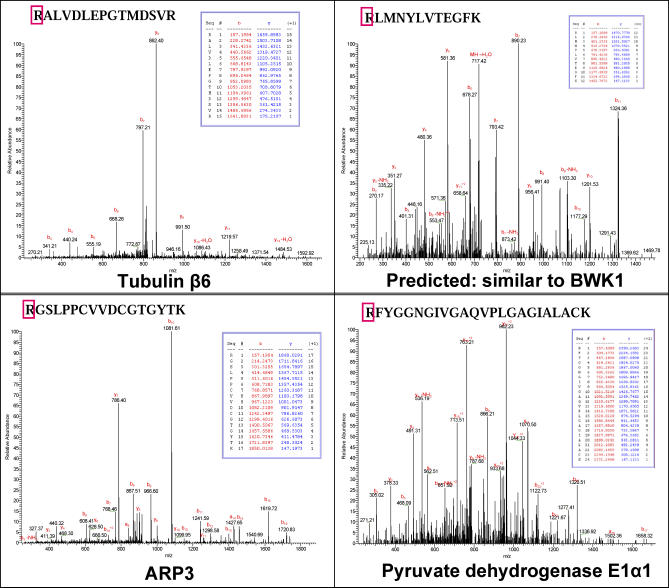
Selected MS/MS Spectra for the Arginylated Peptides Shown in Table 1 Peptide sequences are shown on top of each spectrum, with the added Arg boxed in red. Observed b and y ions are shown in red and blue, respectively on the MS/MS spectra and in the corresponding tables for each spectra. Generic names of the proteins are shown underneath the corresponding spectra. The spectra show peptides arginylated on N-terminal alanine (Ala), Leu, Gly, and phenylalanine (Phe) residues, all of which were previously believed to be unable to serve as an Arg acceptor site. Precursor mass accuracies for the shown spectra are 2.1 ppm (Ala), 3.8 ppm (Leu), 8.0 ppm (Gly), and 5.6 ppm (Phe). See Table 1 for accession numbers and arginylated positions and Dataset S1 for larger scale images of the MS/MS spectra for all identified residues.

Elimination of these ambiguities resulted in reduction of the list of arginylated proteins to a final list of 43 substrates, shown in Table 1. MS/MS spectra of arginylated peptides for these substrates were verified manually and are shown in Figure 4 and Dataset S1 (except beta actin N-terminal arginylated peptide, which was previously published [8]). High mass accuracy of the precursor ions and the characteristic pattern of b ion fragments in these spectra allowed identification of the arginylated positions within the proteins shown in Table 1 with high confidence.

**Table 1:**
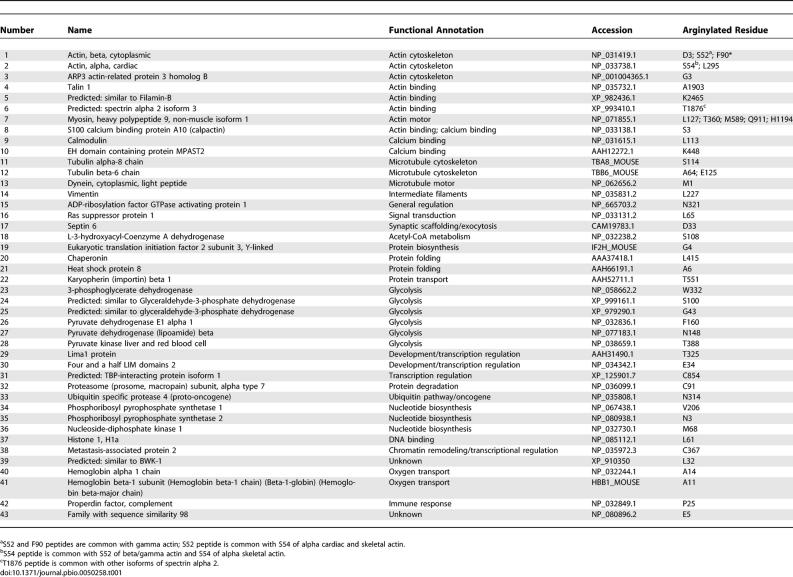
Proteins Arginylated In Vivo

**Figure 5:**
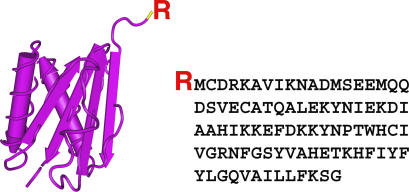
8-kDa Dynein Light Chain Is Arginylated on N-Terminal Met That Is Exposed on the Molecule Surface Crystal structure (left) and primary amino acid sequence (right) of 8-kDa dynein light chain with the arginylated position marked in yellow on the polypeptide chain and denoted with red letter R. Arginylation results in modification of the protein surface, expected to affect molecular interactions and possibly association with the dynein polypeptide complex.

Our analysis revealed several striking findings. First and foremost, we found that arginylation can occur on various N-terminally exposed residues, not only on Asp, Glu, or Cys, as suggested before. We found evidence of almost every residue arginylated on the N-terminus (Figure 4; Dataset S1), including, remarkably, one case of arginylated N-terminal Met (Figure 5 and Figure 19 of Dataset S1). Each protein was found arginylated on a very limited number of sites, which in some cases occurred repeatedly in different samples.

It has been previously suggested that N-terminal asparagine (Asn) and glutamine (Gln) require deamidation in order to be arginylated [22], and that arginylation of N-terminal Cys requires a preceding oxidation [23]. In our analysis, however, we found that Asn and Cys can be arginylated directly without modifications (Figures 10, 14, 26, 35, and 44 of Dataset S1).

As seen from Table 1, eight of the 43 identified proteins were arginylated within several residues of the N-terminus, including the previously identified actin [8] and a closely related protein, ARP3, modified on a similar site. At the same time the remaining 35 proteins were arginylated in the middle of the molecule a significant distance away from the N-terminus. This suggests that such arginylation either occurs on the side chains of the identified residues (in the cases where such side chain modifications are chemically possible, as shown in [2]) or requires a preceding cleavage of the polypeptide chain to expose a free N-terminus of the modified residue.

Analysis of the positions flanking the arginylated residue using the online WebLogo tool (http://weblogo.berkeley.edu/) revealed that there is no apparent preference for particular amino acids in the arginylation site itself and only a weak bias for certain amino acid residues flanking the arginylation site (Figure S1). This apparent absence of the arginylation site motif at the primary structure level suggests that arginylation specificity for certain sites is defined at the secondary or higher structural level.

The identified arginylated proteins shown in Table 1 fall into several distinct functional groups, with a very prominent presence of cytoskeletal proteins and a notable representation of proteins involved in transcriptional regulation and general metabolic pathways. There is no apparent bias toward the overall abundance of arginylated proteins, suggesting that arginylation is highly specific to certain functionally important targets.

**Figure 6:**
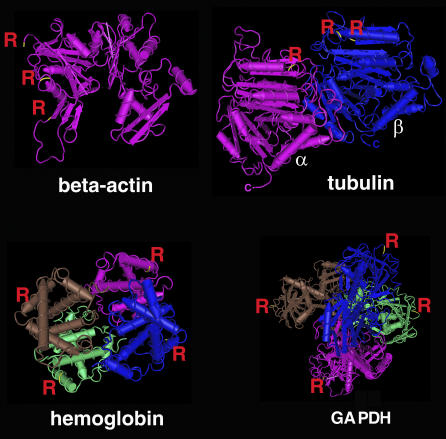
Structural Models of Proteins Regulated by Arginylation Three-dimensional structures of proteins downloaded from the National Center for Biotechnology Information structure database at http://www.ncbi.nlm.nih.gov/ (PDB identifiers 1HLU for beta actin, 1TUB for tubulin, 1DXT for hemoglobin, and 1ZNQ for GADPH) with arginylated positions highlighted in yellow within the polypeptide chains and marked with red letters R. Alpha and beta for tubulin indicate the corresponding polypeptide chains. See Text S1 for explanation of the functional significance of the arginylated sites in each shown protein.

To further assess the possible role of arginylation in modulating the activity of the proteins found on our list, we selected a few targets from our list whose biological role is well characterized and for which three-dimensional structures are available in a public database, including non-muscle actin (PDB identifier 1HLU), tubulin (1TUB), hemoglobin (1DXT), GAPDH (1ZNQ), ARP3 (as part of ARP2/3 complex, 1K8K), 8-kDa dynein light chain (1PWK), and fragment of calmodulin in complex with spectrin (2FOT). We then “mapped” the arginylation sites onto their structures. The most complete molecular structures from this list available in the database are shown in Figures 5 and 6. All mapped arginylation sites (marked with red “R” for proteins shown in Figures 5 and 6) are located on the surface of the assembled molecule or the domain interface between the subunits. In some cases (e.g., vimentin and talin, not shown), arginylated sites are located at the interface between two domains and cleavage at this interface might possibly result in the production of functional protein fragments. Thus, it seems likely that in all of the identified cases arginylation is involved in modifying the surface properties and molecular interactions of the protein targets without major structural or functional perturbations.

## Discussion

In this study we report a systematic characterization of posstranslational protein arginylation and identification of 43 proteins arginylated in vivo in different mouse tissues. Our analysis shows that (1) arginylation can occur on almost any N-terminally exposed residue without an apparent bias; (2) arginylation occurs on a highly limited and conserved number of sites in each protein located on the surface of the assembled molecule, which suggests arginylation target recognition by the secondary or higher order structure; and (3) arginylation is functionally associated with proteins involved in cytoskeleton and primary metabolic pathways.

It has been previously suggested that arginylation can occur only on N-terminally exposed Asp, Glu, and Cys [24] and that such arginylation should result in a decrease in metabolic stability and increase in protein degradation by the ubiquitin-dependent N-end rule pathway [22]. Our study expands this view and demonstrates that arginylation can also occur on other residues and that in many, apparently the majority of cases, it does not result in protein degradation. It has been previously shown that beta actin regulation by arginylation involves structural rearrangements of actin polymers without an effect on its metabolic stability [8]. The current study is in agreement with this finding and suggests that N-terminal arginylation regulates structural properties of a number of other proteins. It should be noted that since our analysis by its nature was biased to proteins abundant (and therefore not degraded) in the wild-type extracts, those proteins that are metabolically destabilized upon arginylation are not a part of the current study, and the conclusions presented here do not take such proteins into account. Therefore, we must acknowledge the possibility that another subset of arginylated proteins exists in vivo that follow different rules than the proteins described here. We expect, however, that some similarities should exist that would be common to the arginylation reaction and its function in vivo.

Since most of the proteins in our analysis were found to be arginylated on the residues internal to the initiator Met, it is clear that for such proteins arginylation involves preceding cleavage by proteases or multiple aminopeptidases, or occurs on the side chains, where chemically possible. It has been previously shown that Glu residue can be arginylated on the side chain [2], which suggests that Asp—which has a similar side chain—can also be arginylated by the same mechanism. Stretching this analogy further, it is also possible to suggest that Asn and Gln can be arginylated with or without deamidation, that lysine (Lys) can accept Arg onto its side chain amino group, and that serine, threonine, and Cys can be arginylated on the side chains after oxidation.

**Figure 7:**
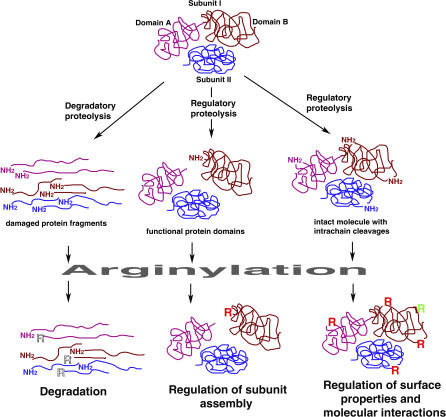
Protein Regulation by Arginylation Top: schematic representation of a protein containing two subunits (I, magenta and brown, and II, blue), whose subunit I is divided into two structurally and functionally distinct subdomains (A and B) within the same polypeptide chain. Middle: proteolytic products of this protein produced by degradation (left), interdomain cleavage (middle), or hypothetical intrachain proteolysis that occurs without the loss of the tertiary structure (right). Bottom: arginylation of the N-termini exposed during these proteolytic events could either produce denatured arginylated peptides (left), modulate the surface properties of active protein fragments (middle), or modulate the surface properties of the intact molecule (right). The last case also includes side chain arginylation, where Arg could be added onto the side chain of Asp or Glu (or possibly other residues) without proteolytic cleavage. Letters R in different colors indicate Arg residues added onto the damaged protein products (white), functional sites on the surface of proteins or domains (red), or side chains exposed on the protein surface (green).

The finding that a large subset of arginylation sites are either exposed on the molecule surface or located at a clear boundary between two protein domains suggests an interesting possibility of protein regulation by arginylation (Figure 7). While it is possible that in some cases arginylation occurs after degradatory proteolysis of damaged proteins (left branch of the pathway in Figure 7), it is notable that apparently only a relatively small fraction of the peptides in vivo are arginylated, suggesting that the specificity for the arginylated sites must be fairly high. In fact, as shown here, this specificity may involve structural motifs on the protein surface and amino acids within the polypeptide located on the N-terminal side of the arginylated residues (Figure S1), suggesting that the recognition of the arginylated sites occurs prior to proteolytic cleavage. It is also notable that, based on the known three-dimensional structures, the majority of the arginylated sites are accessible for arginylation on the surface of the intact protein molecules or subunits (as seen in the cases shown in Figures 5 and 6 and observed for all other analyzed structures) or after an interdomain cleavage that would be likely to produce functional protein fragments (middle branch of the pathway in Figure 7). Based on the facts that recognition of the arginylated sites appears to precede proteolytic cleavage and that such sites in many cases are found in a limited number of surface sites, we propose a new mechanism for protein regulation that would involve limited proteolysis by “snipping” the polypeptide chain without breaking down the tertiary structure of the molecule, and the subsequent arginylation (right branch of the pathway in Figure 7). It seems possible that such proteolysis could happen in vivo without breaking down the quaternary structure of the protein molecule, held together by noncovalent interactions or covalent crosslinking of the amino acid side chains [25]. In fact, internal arginylation sites are found in proteins (shown in Tables 1 and S3) that are excised from the SDS gel spots of their expected molecular weight, suggesting that covalent crosslinking in vivo is holding them together after internal cleavage and arginylation. This possibility constitutes an exciting direction of further study.

Additional levels of regulation of protein arginylation could be imagined. First, it is possible that for those substrates that are arginylated after denaturation and cleavage, addition of N-terminal Arg causes their degradation, as has been suggested by the N-end rule studies. Such a mechanism would involve massive proteolysis in wild-type cells and accumulation of the corresponding targets in the *Ate1* ^−/−^ cells, which could account for a subset of arginylation-related changes observed in our analysis. Based on our data and the work of other groups, there is currently no solid evidence that this mechanism regulates a large number of arginylated proteins, but we cannot exclude the possibility that some of the arginylated targets are indeed destabilized, in a specific or nonspecific way.

A second possibility of arginylation regulation concerns competition with other posttranslational modifications for the free N-termini. It has been previously shown that a majority of eukaryotic proteins are acetylated in vivo [26,27]. It seems likely that acetylation and arginylation reactions, which can occur on the same alpha amino groups of the same residues, are in competition in vivo and that the fractions of acetylated versus arginylated forms of each protein are regulated by this competition. Such a situation most likely happens in the case of beta actin, which is found in vivo in both acetylated and arginylated forms [8].

Finally, regulation of arginylation is likely to involve de-arginylation enzymes that should cleave off N-terminal Arg to regulate the fraction of arginylated protein versus its non-arginylated form in important metabolic pathways. A likely candidate for such a role is aminopeptidase B [28], which selectively removes Lys and Arg from the N-terminus of certain regulatory peptides and could feasibly perform a similar function for other arginylated proteins. It is also possible that other de-arginylation enzymes with broad or narrow specificity exist in vivo and may be involved in regulation of arginylation.

The results of this study suggest that arginylation is a global form of regulation of activity, abundance, and structure of multiple proteins in vivo, reminiscent of protein phosphorylation, and that it involves its own machinery of arginyltransferases, cofactors, and de-arginylation enzymes that is responsible for controlling vital physiological processes. Like protein phosphorylation, arginylation is likely to regulate different proteins in different ways that would modulate their assembly and structure (as seen in the case of beta actin), metabolic stability (as seen with regulators of G-protein signaling RGS4, RGS5, and RGS16), or other properties that facilitate their intracellular functions and roles in cardiovascular development, angiogenesis, and other important biological processes. Studies of this modification constitute an exciting emerging field.

## Materials and Methods

### Protein fractionation and analysis.

Comparative 2D gel electrophoresis shown in Figure 1 was performed using frozen cell pellets prepared from cell monolayers of wild-type cells, wild-type cells treated with 2 mM proteasome inhibitor MG-132 (Sigma-Aldrich, http://www.sigmaaldrich.com/), and *Ate1* ^−/−^ cells, collected by scraping and centrifugation for 5′ at 1,000 rpm. Samples were lysed in 2D lysis buffer containing 30 mM Tris-HCl (pH 8.8), 8 M urea, and 4% CHAPS, and sonicated for 5 s using VirSonic 100 (VirTis, http://www.virtis.com/) at power level 4, followed by vigorous shaking at room temperature for 30 min and clarification by high-speed centrifugation for 30 min. Supernatants were transferred to fresh eppendorf tubes, and protein concentration was adjusted to 5 mg/ml for each sample with lysis buffer. For Cy dye labeling, Cy2, Cy3, and Cy5 (Amersham, http://www.amersham.com/) were diluted 1:5 with dimethyl formamide prior to use, mixed with the samples, and incubated on ice for 30 min, followed by addition of 0.7 μl of 10 mM Lys and additional incubation on ice in the dark for 15 min. For the 2D gel analysis, equal amounts of samples were mixed with the sample buffer and fractionated as described in [29]. Gels were visualized in three fluorescence channels, scanned using Typhoon Trio scanner (Amersham), and analyzed using ImageQuant software for imaging and DeCyder software for quantification (Amersham). Sample preparation from frozen cell pellets, 2D gel fractionation, and analysis of the data shown in Figure 1 were performed with the help of Applied Biomics (http://www.appliedbiomics.com/). For the bar chart in Figure 1B and Table S4, spots up- or down-regulated by 1.5-fold or more were considered.

Gels for protein identification shown in Tables S1–S3 were run and analyzed by Kendrick Laboratories (http://www.kendricklabs.com/) as described below. For manual computerized comparisons of protein abundance in the spots used for spot excision and protein identification shown in Tables S1–S3, integrated density (volume) for each spot on the high-resolution scanned images of gels or autoradiographs was expressed as a percentage of total density of all spots measured, and the difference in abundance was defined by the fold-change of the spot percentage value compared to the spot's matched counterpart in the comparison gel. Spots with altered volume percentage were defined by the ratio of the percentages in the two gels and defined as positive (for up-regulation) or negative (for down-regulation). Gels were analyzed in duplicates and Student's *t*-test values were generated by the software for the *n*-fold change averaged from two gels. For protein identification, spots up- or down-regulated 2-fold or more were considered; the most abundant of these spots were subjected to MS identification, with the results shown in Tables S1–S3.

### Posttranslational incorporation of Arg into cultured cells.

This analysis was performed as described in [9] with some modifications. Cell monolayers were placed into custom-prepared serum-free DMEM without Arg supplemented with 10 μg/ml cycloheximide and incubated for 3 h to suppress protein synthesis and induce Arg starvation. Medium was then supplemented with 5 μCi of ^14^C-Arg (Moravek Biochemicals, http://www.moravek.com/) and incubated for additional 3 h at 37 °C under normal growth conditions. After incubation, medium was removed and cells were dissolved in SDS sample buffer for 2D electrophoresis shown in Figure 2. 2D gels and autoradiography were performed by Kendrick Laboratories. For identification of proteins in the spots that showed increased Arg incorporation above the *Ate1* ^−/−^ background, duplicate gels of untreated cell extracts were run, and spots were excised from the Coomassie-stained gels superimposed on the autoradiographs shown in Figure 2. Protein identification for this experiment was performed by Midwest Bio Services (http://www.midwestbioservices.com/).

### Antibody generation and immunoaffinity chromatography.

Initial synthesis of the peptides shown in Figure 3, immunizations, and collection of antisera were performed by Sigma Genosys (http://www.sigmaaldrich.com/Brands/Sigma_Genosys.html). Crude antiserum was affinity purified on the peptides used for immunization and then immunodepleted as shown in Figure 3 using agarose beads with covalently linked peptides, and purified antibodies were covalently coupled to Aminolink agarose (Pierce). Whole lysates of mouse organs (brain, liver, kidney, spleen, heart, and lungs), embryonic day 12 embryos, and cultured fibroblasts, clarified by high-speed centrifugation, were preabsorbed on the column with coupled preimmune serum. The resulting extracts were bound to the antibody columns, and arginylated proteins were eluted with Gly (pH 2.7) or 3.5 M MgCl_2_. Protein mixtures were precipitated with trichloroacetic acid, resuspended in physiological buffer, and digested with a cocktail of proteases including various combinations of trypsin, subtilisin, elastase, and endopeptidase Lys-C. Proteins in the resulting digests were identified by MS as described below.

### Peptide synthesis.

Standard peptides for the validation of the MS data and performing immunoaffinity purifications and antibody characterization during the later stages of the project were synthesized in collaboration with the Proteomics Resource Center, Rockefeller University (http://pdtc.rockefeller.edu/). All peptides were created using a SYMPHONY multiple peptide synthesizer (Protein Technologies, http://www.pti-instruments.com/) on Wang resin (Bachem, http://www.bachem.com/) using Fmoc protected amino acids (AnaSpec, http://www.anaspec.com/) [30]. Coupling reactions were conducted using 0.3 M HBTU or HATU/HOBT and 0.4 M NMM or 0.6 M DIPEA in N-methylpyrrolidinone as the primary solvent [31]. Simultaneous resin cleavage and side chain deprotection were achieved by treatment with concentrated, sequencing-grade trifluoroacetic acid with triisopropylsilane, water, and ethanedithiol in a ratio of 95:2:2:1 for a 3- to 6-h time frame. Rotary evaporation followed by high vacuum overnight was used to remove trifluoroacetic acid from the resin-bound peptides. Peptides were then released in 8 M acetic acid and filtered from resin, the acidic mixture was evaporated, and the peptides were redissolved in HPLC-grade water for lyophilization. All crude lyophilized products were subsequently analyzed by reversed-phase (RP) HPLC (Waters, http://www.waters.com/) using a Merck (http://www.merck.com/) Chromolith Performance C18 column. Individual peptide integrity was verified by matrix-assisted laser desorption ionization (MALDI) MS using a PerSeptive/Applied Biosystems (http://www.appliedbiosystems.com/) Voyager delayed extraction spectrometer system [32].

### Single RP and multi-dimentional (MudPIT) liquid chromatography, MS, and database searching.

Protein digestion was performed in both gel and solution forms. The cut gel pieces were washed by Milli-Q water (Millipore, http://www.millipore.com/) and a 1:1 mixture of water/acetonitrile, and destained with pure acetonitrile and 100 mM ammonium bicarbonate (Sigma-Aldrich). Protein pellet was dissolved in Invitrosol LC/MS protein solubilizer following the manufacturer's protocol (Invitrogen, http://www.invitrogen.com/). The solution was reduced and alkylated with 10 mM Tris(2-carboxyethyl)phosphine hydrochloride (Roche Applied Science, http://www.roche-applied-science.com/) and 55 mM iodoacetamide (Sigma-Aldrich) in 100 mM ammonium bicarbonate. Digestion was performed in the presence of 50 mM ammonium bicarbonate and 5 mM calcium chloride (Sigma-Aldrich) using sequencing-grade soluble trypsin (Promega, http://www.promega.com/) or endopeptidase Lys-C (Roche Applied Science, http://www.roche-applied-science.com/). The resulting peptides were extracted by 5% formic acid and redissolved into buffer A (5% acetonitrile with 0.1% formic acid) prior to the liquid chromatography MS/MS analysis. Peptide mixtures were pressure-loaded onto a RP or strong cation exchange/RP MudPIT column and eluted with a linear gradient of acetonitrile or pulses of ammonium acetate salt (5%–100%). Synthetic peptides were dissolved in water and supplemented with 1% formic acid to the final concentration of 100 fmol/μl and analyzed under the same liquid chromatography MS/MS conditions as those used for the real samples.

Data-dependent MS/MS analysis was performed in a LTQ-Orbitrap mass spectrometer (Thermo Fisher Scientific, http://www.thermo.com/). Full MS spectra were acquired by the precursor ion scan using the Orbitrap analyzer with resolution set at 60,000, followed by nine MS/MS events in the linear ion trap (LTQ), sequentially generated on the first-, second-, and third-most intense ions selected from the full MS spectrum. MS scan functions and HPLC solvent gradients were controlled by the Xcalibur data system (Thermo Fisher Scientific). Tandem mass spectra were searched against European Bioinformatics Institute International Protein Index mouse protein database (version 3.06, release date 10 May 2005; http://www.ebi.ac.uk/IPI/) that was concatenated with reversed sequences to estimate false positive rate using the ProLuCID [33] protein database search algorithm with peptide mass tolerance of ±3 amu, fragment ion mass tolerance of ±0.4 amu, and a static modification of 57.0215 on Cys due to carboxyamidomethylation. This program has been modified to add the capability of performing N- and C-terminal differential modification searches. In order to identify N-terminal arginylated peptides, differential N-terminal modification of 156.1011 (the monoisotopic mass of an Arg residue) searches were performed with no enzymatic cleavage conditions imposed on the database search. For each spectrum, ProLuCID searches the protein database twice. On the first round it assumes no modification (mass shift) on any candidate peptides, and on the second round it assumes that each peptide has a mass shift of 156.1011 at the N-terminus of the peptides. It is worth noting that users do not need to specify what residues can have the modification. An N-terminal modification is considered for each peptide within the given mass tolerance, regardless of what residue is located at the N-terminus. The program computes a binomial probability score for each candidate peptide based on the number of theoretical peaks that match experimental peaks and the complexity of the spectra. Like SEQUEST [20,21], for each spectrum, ProLuCID selects 500 peptides that are least likely to be random hits based on the binomial probability score and applies an improved cross-correlation algorithm to compute a cross-correlation score (XCorr) and a normalized difference in cross-correlation score (DeltaCN) for each of these 500 peptides. The final results are sorted by XCorr.

ProLuCID search results were then filtered with DTASelect 2.0 [34] using XCorr and DeltaCN at a false positive rate of 0.1% (–fp 0.001) estimated by number of reverse hits. Only half or fully tryptic (–y 1) peptides with N-terminal arginylation (–m 0) and a DeltaMass ≤ 5 ppm (–DM 5) were accepted; the minimum number of peptides to identify a protein was set to 1(–p 1). The final proteins with arginylation modifications that passed these filtering criteria and manual validation are listed in Table 1.

## Supporting Information

Dataset S1 MS/MS Spectra of the N-Terminally Arginylated Peptides for Proteins Shown in Table 1
Peptide sequence, accession number, and protein name are listed on top of each spectrum. N-terminally arginylated Ile, Gln, and Arg were not found. One protein was found to be arginylated on initiator Met (Figure 19), and two more cases of arginylation on internal Met have been found (Figures 5 and 20).
(485 KB PDF)

Dataset S2 MS/MS Spectra for the Standard Peptides Used to Differentiate N-Terminal Arg (Figure 1) and Val–Gly (Figure 2) Residues in the Peptides While precursor masses of the two peptides are similar, the arginylated peptide shows prominent peaks for b ions, while the spectrum for the peptide with N-terminal Val–Gly is abundant in y ions only.
(34 KB PDF)

Figure S1 Frequencies of Occurrence of Different Amino Acids in the Arginylated Position and 20 Flanking Positions on Each Side of the Arginylated Residue Calculated with the Online WebLogo Tool Letter size corresponds to the frequency of the residue occurrence in each position adjusted to the total frequency of its occurrence in vivo.
(23 KB PDF)

Table S1 Proteins Up- or Down-Regulated in Response to *Ate1* Knockout (128 KB DOC)

Table S2 Proteins Up-Regulated in Response to Proteasome Inhibition (46 KB DOC)

Table S3 Proteins Identified in the Spots That Posttranslationally Incorporate Arg in Cultured Fibroblasts (162 KB DOC)

Table S4 2D Gel Protein Spot Comparison between Wild-Type Cells, Wild-Type Cells Treated with Proteasome Inhibitor, and *Ate1* ^−/−^ Cells WT, wild-type cells; WTPI, wild-type cells treated with proteasome inhibitor.
(33 KB DOC)

Text S1 Detailed Discussion of Figure 6: Possible Ways of Regulation of Actin, Tubulin, Hemoglobin, and GAPDH by Arginylation (56 KB DOC)

### Accession Numbers

The Mouse Genome Informatics (http://www.informatics.jax.org/) IDs for proteins discussed in this paper are actin (87904), aminopeptidase B (2384902), ARP3 (2661120), beta actin (87904), talin (1099832), and vimentin (98932).

## Abbreviations

2D: two-dimensional
MS: mass spectrometry
MS/MS: tandem mass spectrometry
RP: reversed-phase
