# Supplementary material for: Global Analysis of Posttranslational Protein Arginylation
Source: PLoS Biol. 2007 Sep 25;5(10):e258. doi: 10.1371/journal.pbio.0050258 (PMC1988855; doi:10.1371/journal.pbio.0050258)
Supplement: Supplementary file 1 [file pbio.0050258.sd001.pdf]

Fig. 1-44. MS/MS spectra of the N-terminally arginylated peptides for proteins shown in Table 1 of the main text. Peptide sequence, accession number, and protein name are listed on top of each spectrum. N-terminally arginylated I, Q, and R were not found. One protein was found to be arginylated on initiator Met (Figure 19) and two more cases of arginylation on internal Met have been found (Figures 5 and 20).

Figure 1

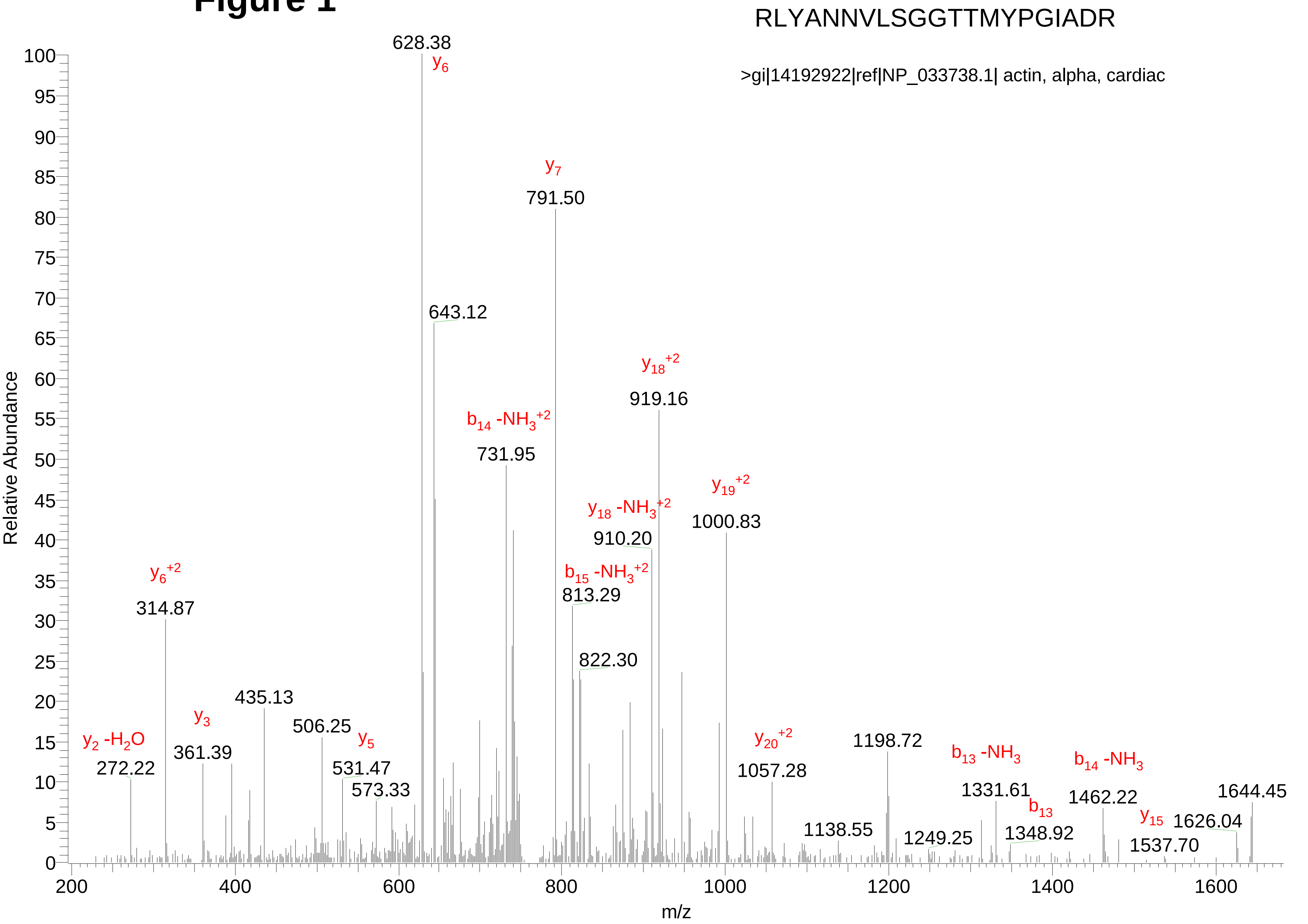

RLYANNVLSGGTTMYPGIADR
>gi|14192922|ref|NP_033738.1| actin, alpha, cardiac
Relative Abundance
m/z
628.38
y6
y7
791.50
643.12
y18+2
919.16
b14 -NH3+2
731.95
y19+2
1000.83
y18 -NH3+2
910.20
b15 -NH3+2
813.29
y6+2
314.87
822.30
435.13
y3
506.25
y5
y2 -H2O
361.39
272.22
531.47
573.33
y20+2
1057.28
1198.72
b13 -NH3
b14 -NH3
1331.61
1462.22
1644.45
b13
y15
1626.04
1138.55
1348.92
1249.25
1537.70

Figure 2

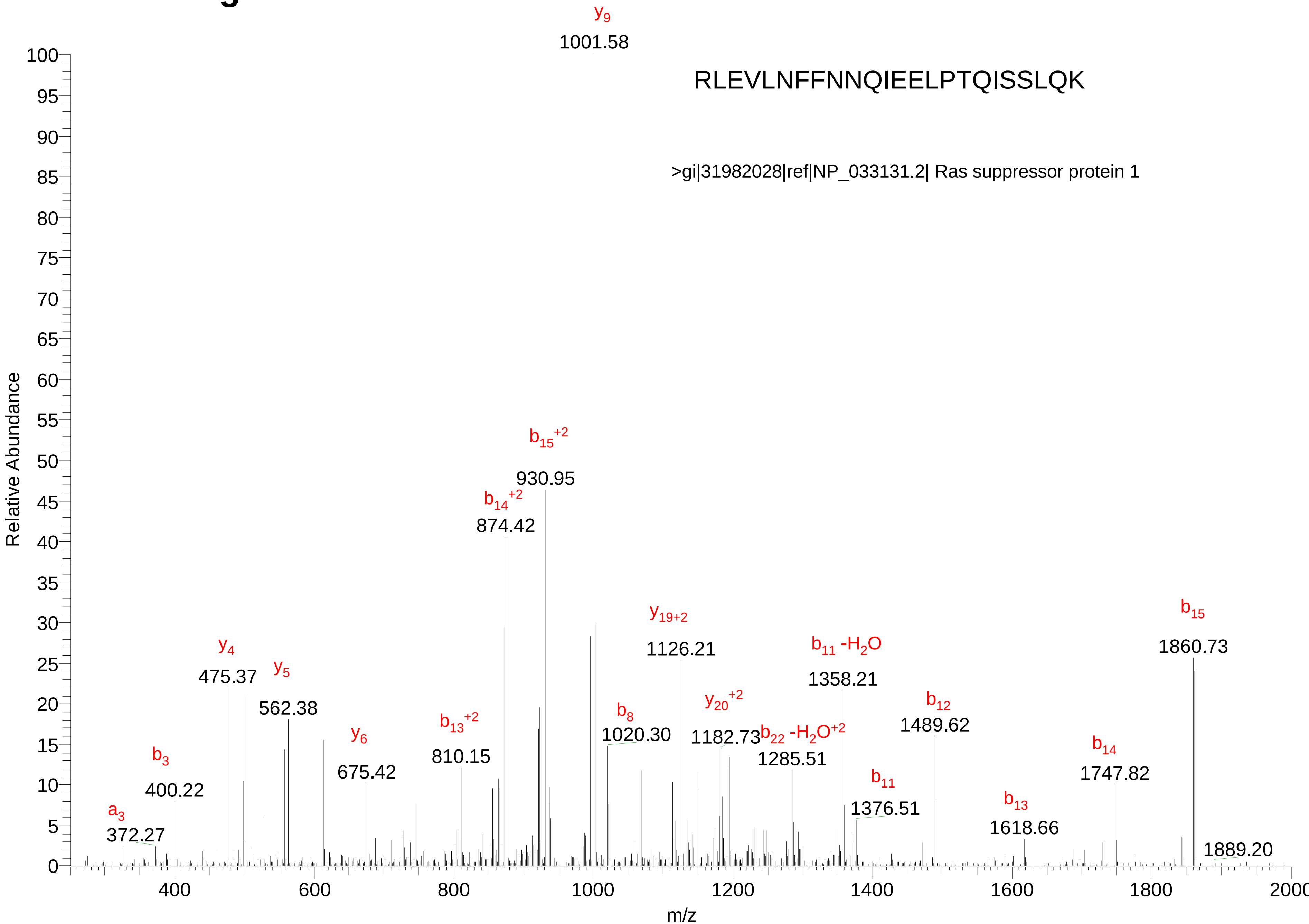
RLEVLNFFNNQIEELPTQISSLQK
>gi|31982028|ref|NP_033131.2| Ras suppressor protein 1
Relative Abundance
m/z
y9 1001.58
b15+2 930.95
b14+2 874.42
y19+2 1126.21
b15 1860.73
b11 -H2O 1358.21
y4 475.37
y5 562.38
b8 1020.30
y20+2 1182.73
b22 -H2O+2 1285.51
b12 1489.62
b13+2 810.15
y6 675.42
b3 400.22
a3 372.27
b11 1376.51
b14 1747.82
b13 1618.66
1889.20

Figure 3

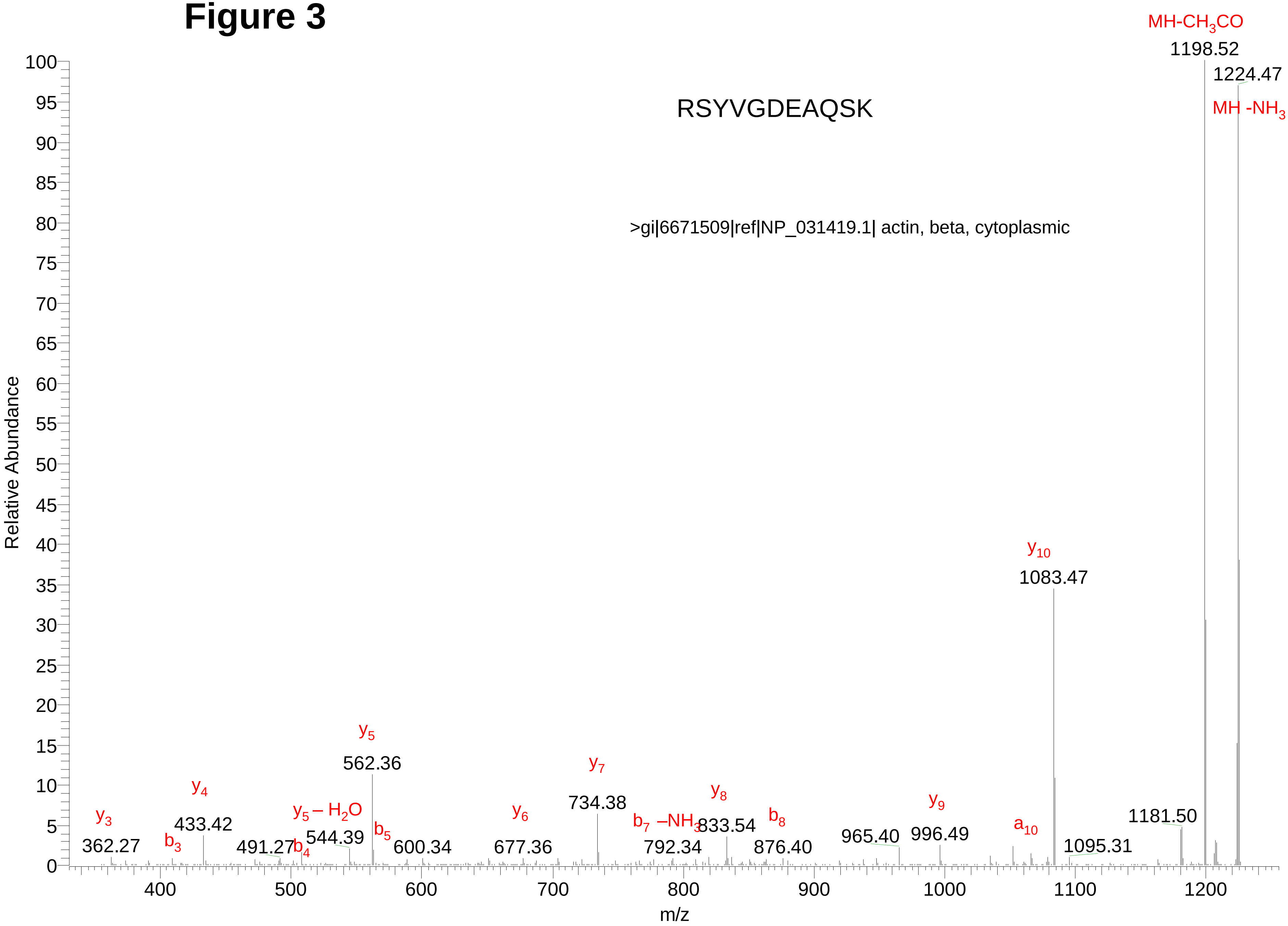

RSYVGDEAQSK
>gi|6671509|ref|NP_031419.1| actin, beta, cytoplasmic
MH-CH3CO
1198.52
1224.47
MH -NH3
y10
1083.47
y5
562.36
y7
734.38
y3
362.27
y4
433.42
b3
491.27
b4
y5 – H2O
544.39
b5
600.34
y6
677.36
b7 –NH3
792.34
y8
833.54
b8
876.40
965.40
y9
996.49
a10
1095.31
1181.50
Relative Abundance
m/z

Figure 4

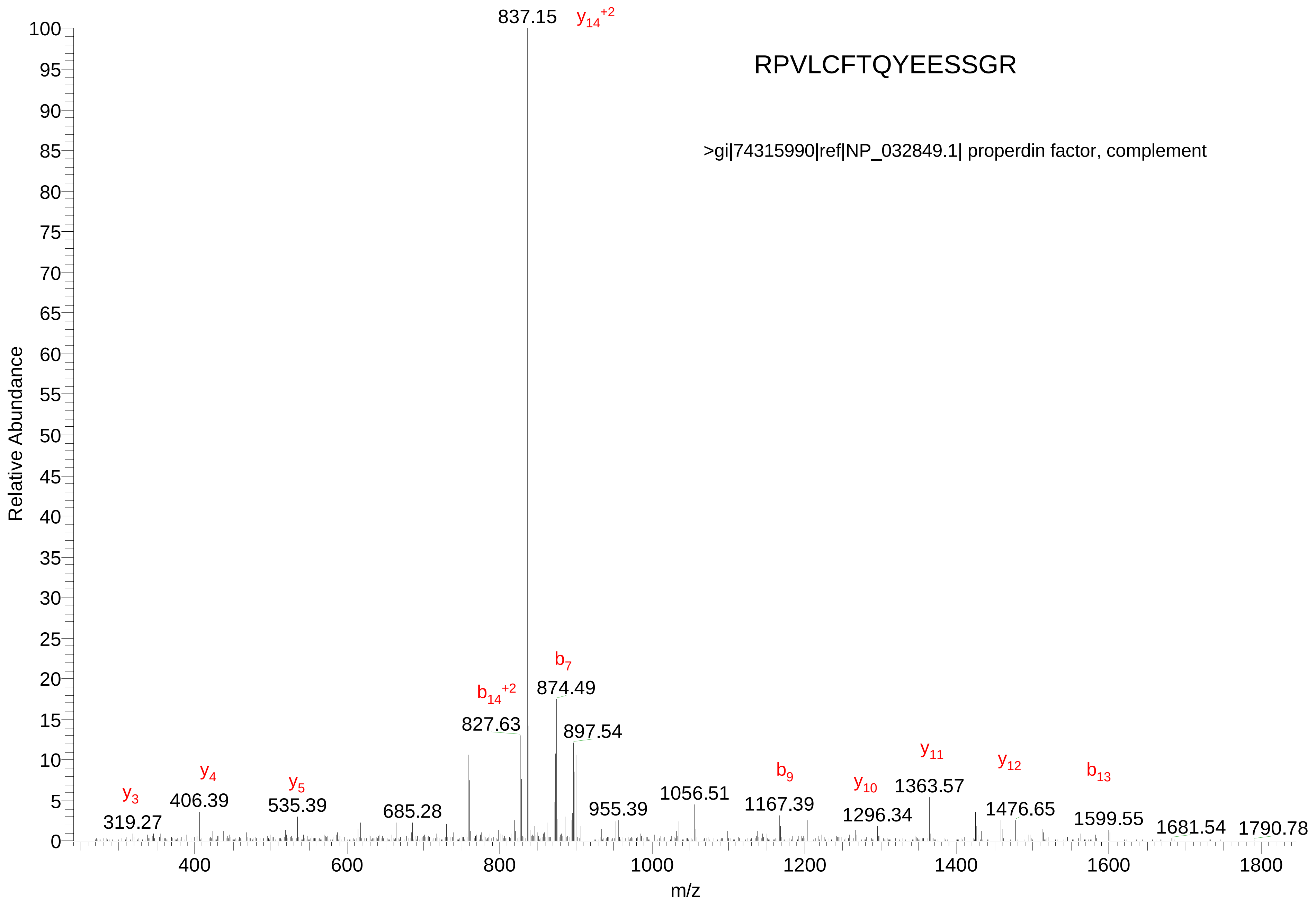

837.15
y_{14}^{+2}
RPVLCFTQYEESSGR
>gi|74315990|ref|NP_032849.1| properdin factor, complement
Relative Abundance
m/z
b_7
874.49
b_{14}^{+2}
827.63
897.54
y_3
319.27
y_4
406.39
y_5
535.39
685.28
955.39
1056.51
b_9
1167.39
y_{10}
1296.34
y_{11}
1363.57
y_{12}
1476.65
b_{13}
1599.55
1681.54
1790.78

Figure 5

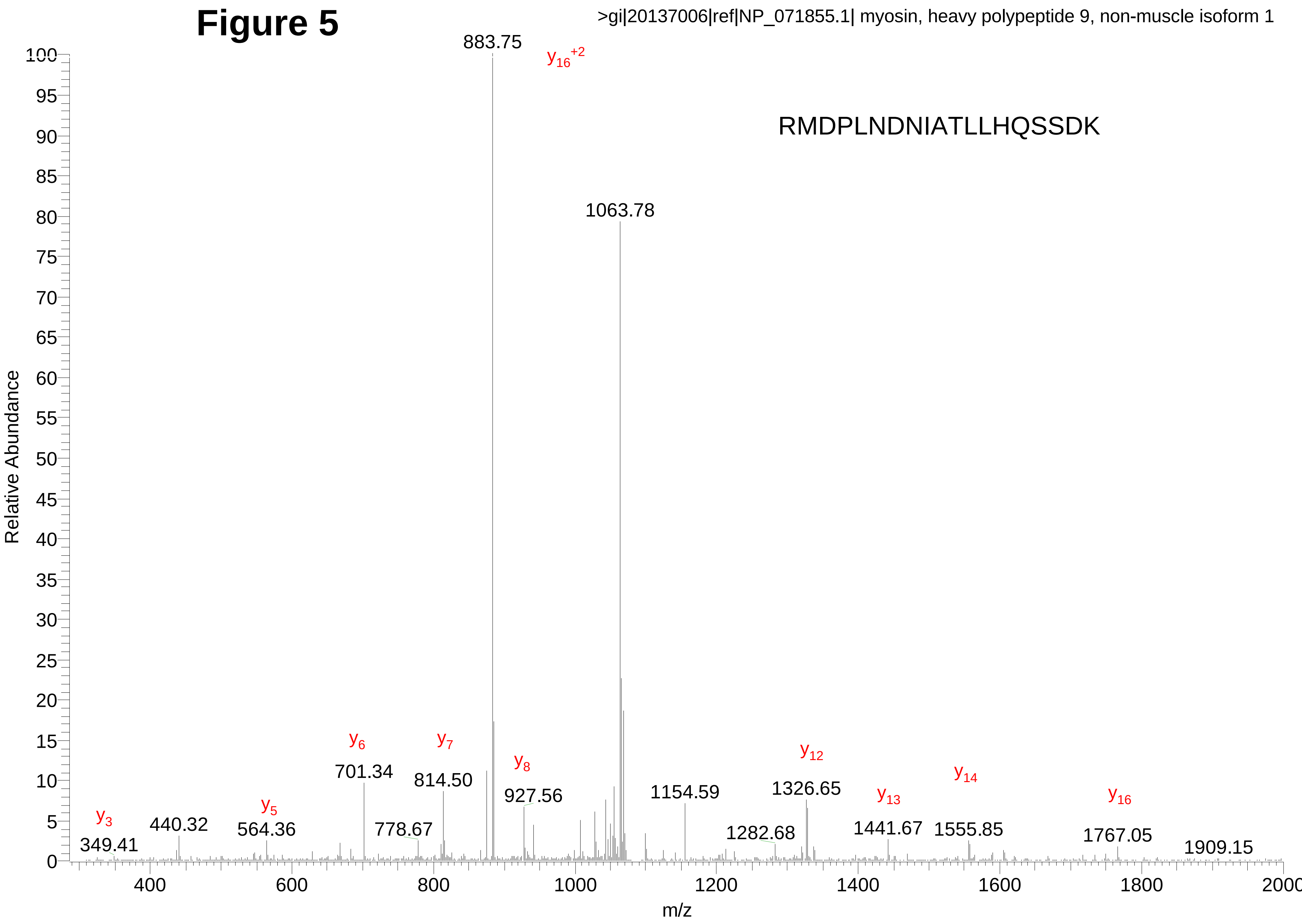
>gi|20137006|ref|NP_071855.1| myosin, heavy polypeptide 9, non-muscle isoform 1
RMDPLNDNIATLLHQSSDK
883.75
y16+2
1063.78
y3
349.41
440.32
y5
564.36
y6
701.34
778.67
y7
814.50
y8
927.56
1154.59
1282.68
y12
1326.65
y13
1441.67
y14
1555.85
y16
1767.05
1909.15
Relative Abundance
m/z
0
5
10
15
20
25
30
35
40
45
50
55
60
65
70
75
80
85
90
95
100
400
600
800
1000
1200
1400
1600
1800
2000

Figure 6

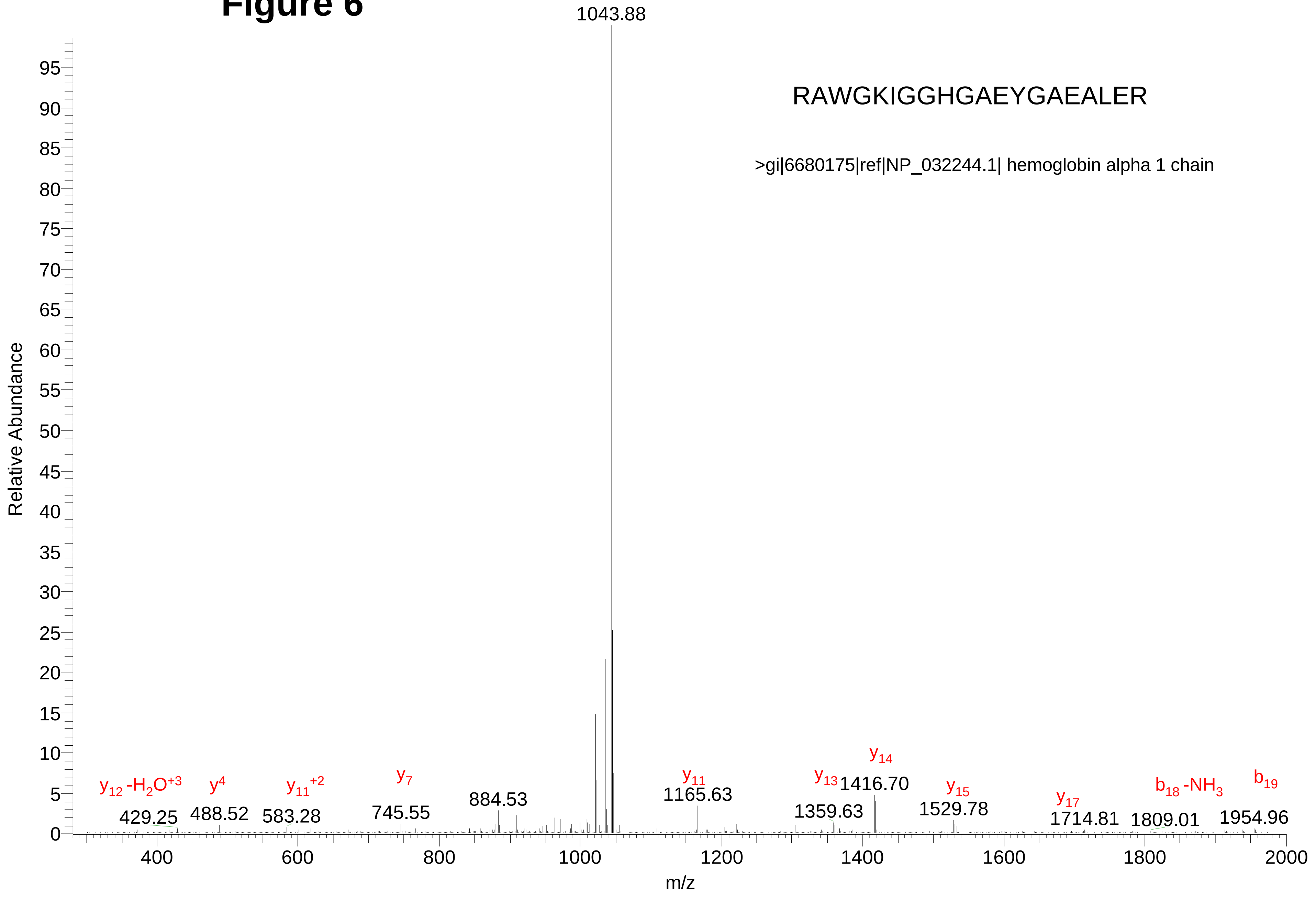

1043.88
RAWGKIGGHGAEYGAEALER
>gi|6680175|ref|NP_032244.1| hemoglobin alpha 1 chain
Relative Abundance
m/z
y12 -H2O+3
429.25
y4
488.52
y11+2
583.28
y7
745.55
884.53
y11
1165.63
y13
1359.63
y14
1416.70
y15
1529.78
y17
1714.81
b18 -NH3
1809.01
b19
1954.96

Figure 7

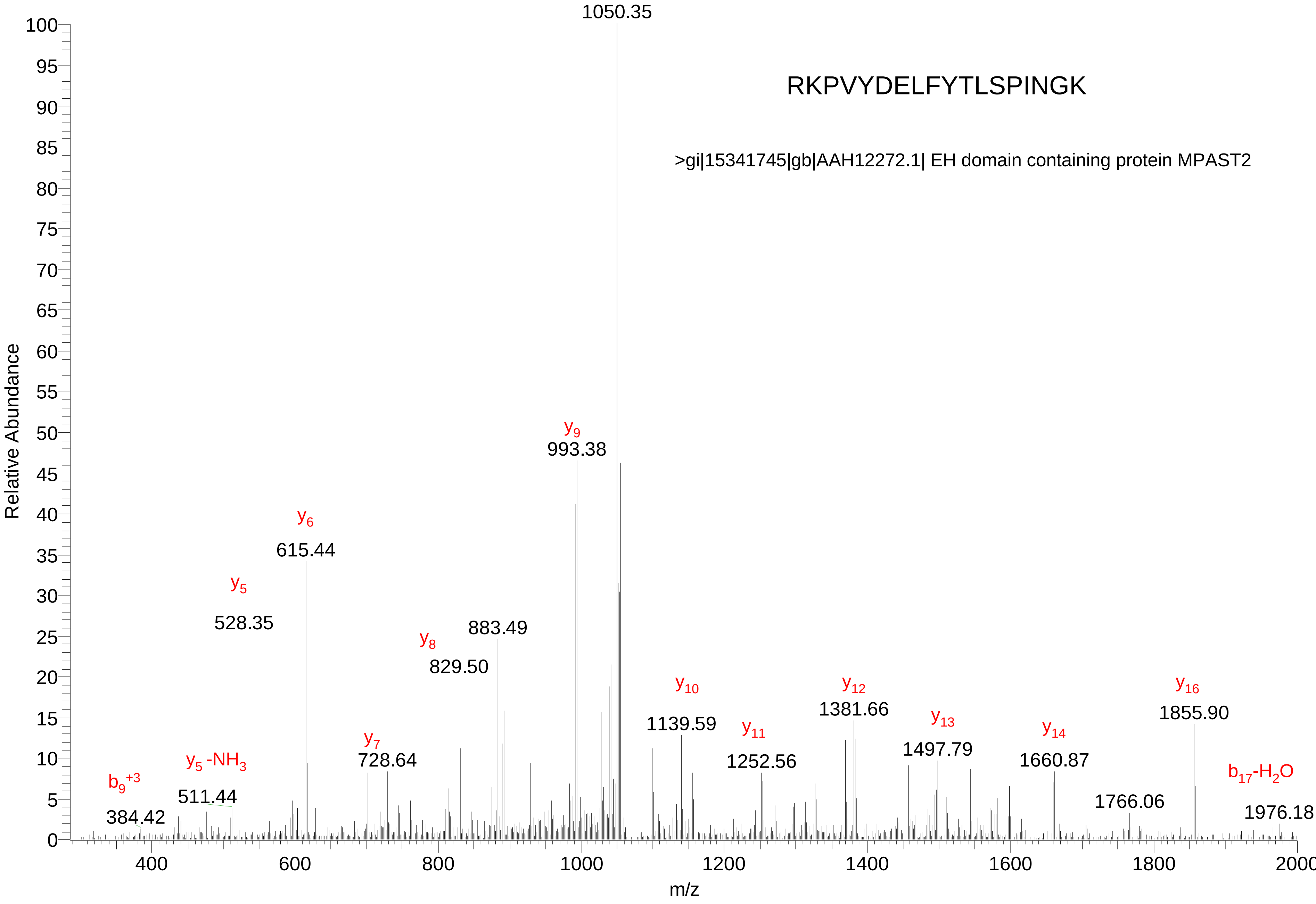

1050.35
RKPVYDELFYTLSPINGK
>gi|15341745|gb|AAH12272.1| EH domain containing protein MPAST2
y9
993.38
y6
615.44
y5
528.35
y8
829.50
883.49
y10
1139.59
y11
1252.56
y12
1381.66
y13
1497.79
y14
1660.87
y16
1855.90
y7
728.64
y5 -NH3
511.44
b9+3
384.42
1766.06
b17-H2O
1976.18
Relative Abundance
m/z

Figure 8

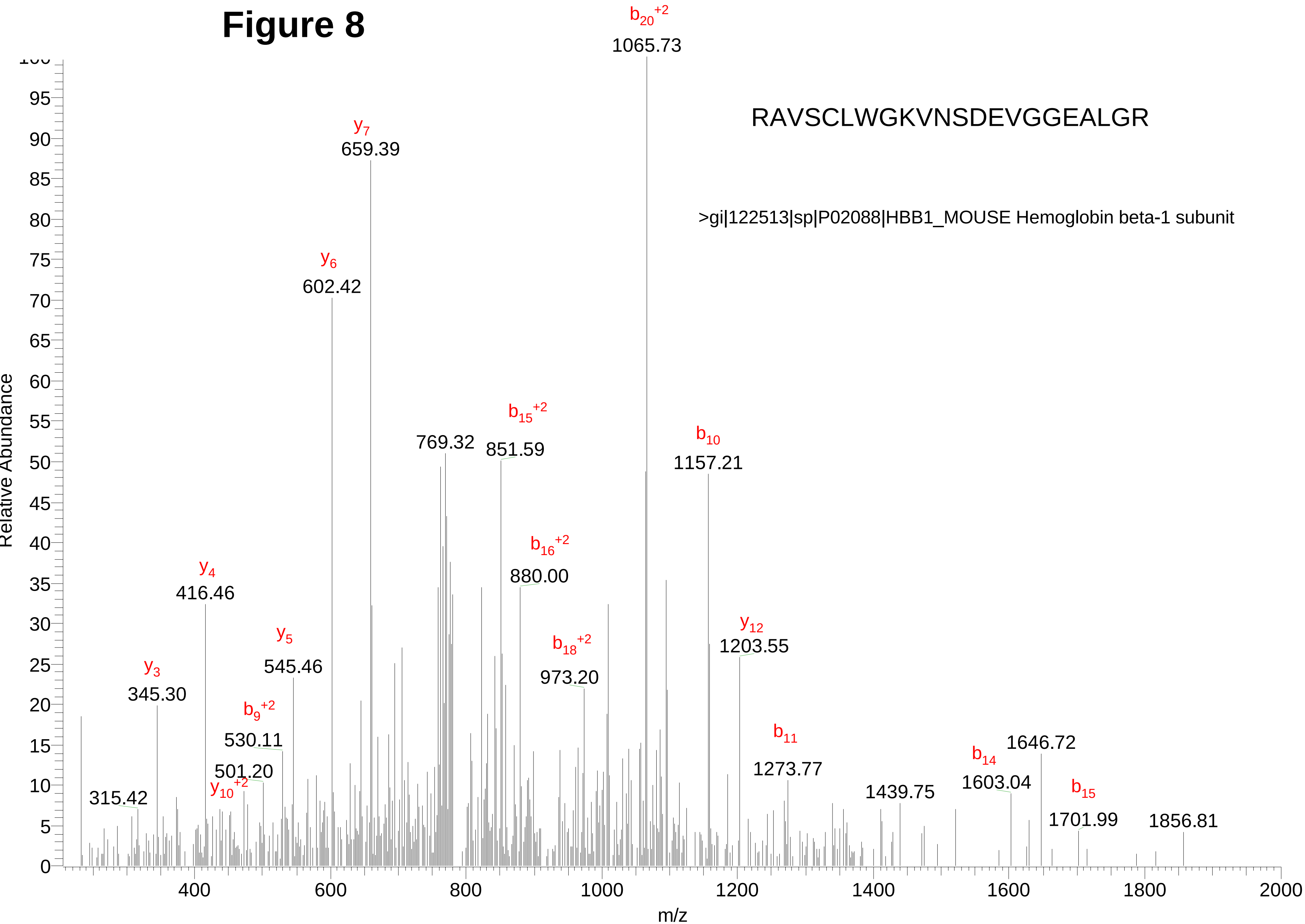
RAVSCLWGKVNSDEVGGEALGR
>gi|122513|sp|P02088|HBB1_MOUSE Hemoglobin beta-1 subunit
Relative Abundance
m/z
b20+2
1065.73
y7
659.39
y6
602.42
769.32
b15+2
851.59
b10
1157.21
b16+2
880.00
y4
416.46
y5
545.46
b18+2
973.20
y12
1203.55
y3
345.30
b9+2
530.11
501.20
y10+2
315.42
b11
1273.77
b14
1603.04
1646.72
1439.75
b15
1701.99
1856.81

>gi|94408118|ref|XP_979290.1| PREDICTED: similar to glyceraldehyde-3-phosphate dehydrogenase

Figure 9

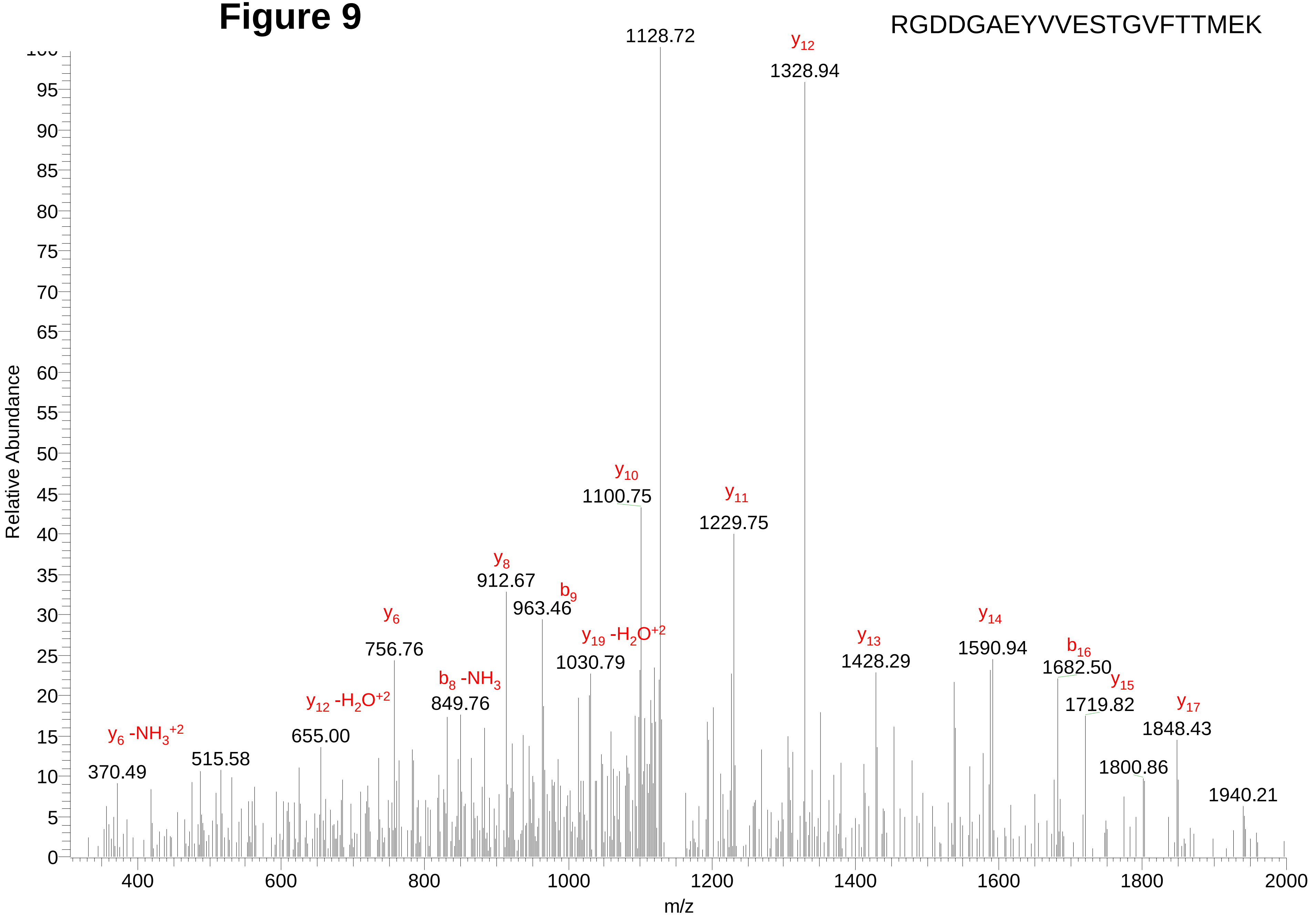

Figure 10

>gi|6755943|ref|NP_035808.1| ubiquitin specific protease 4

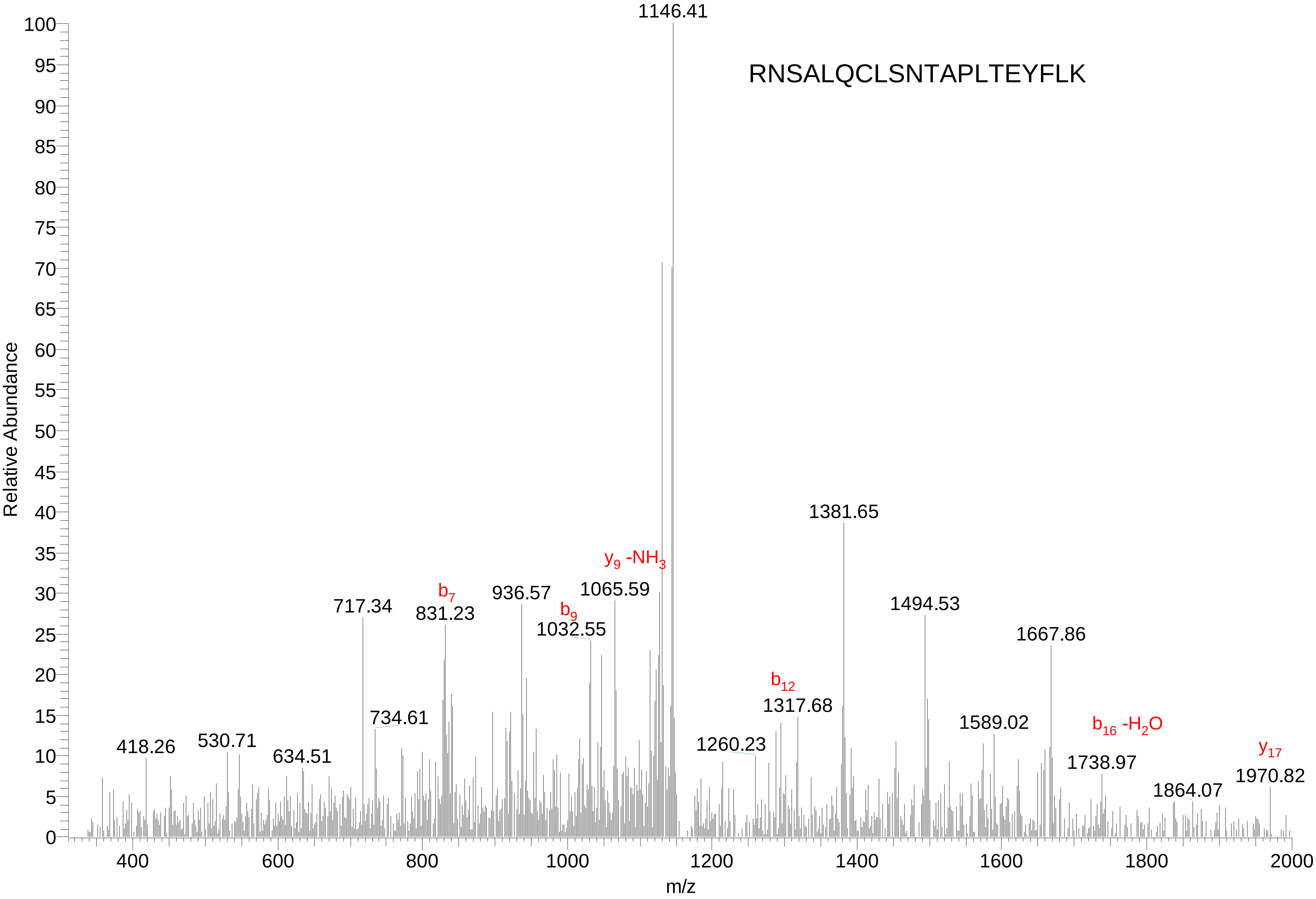

Figure 11

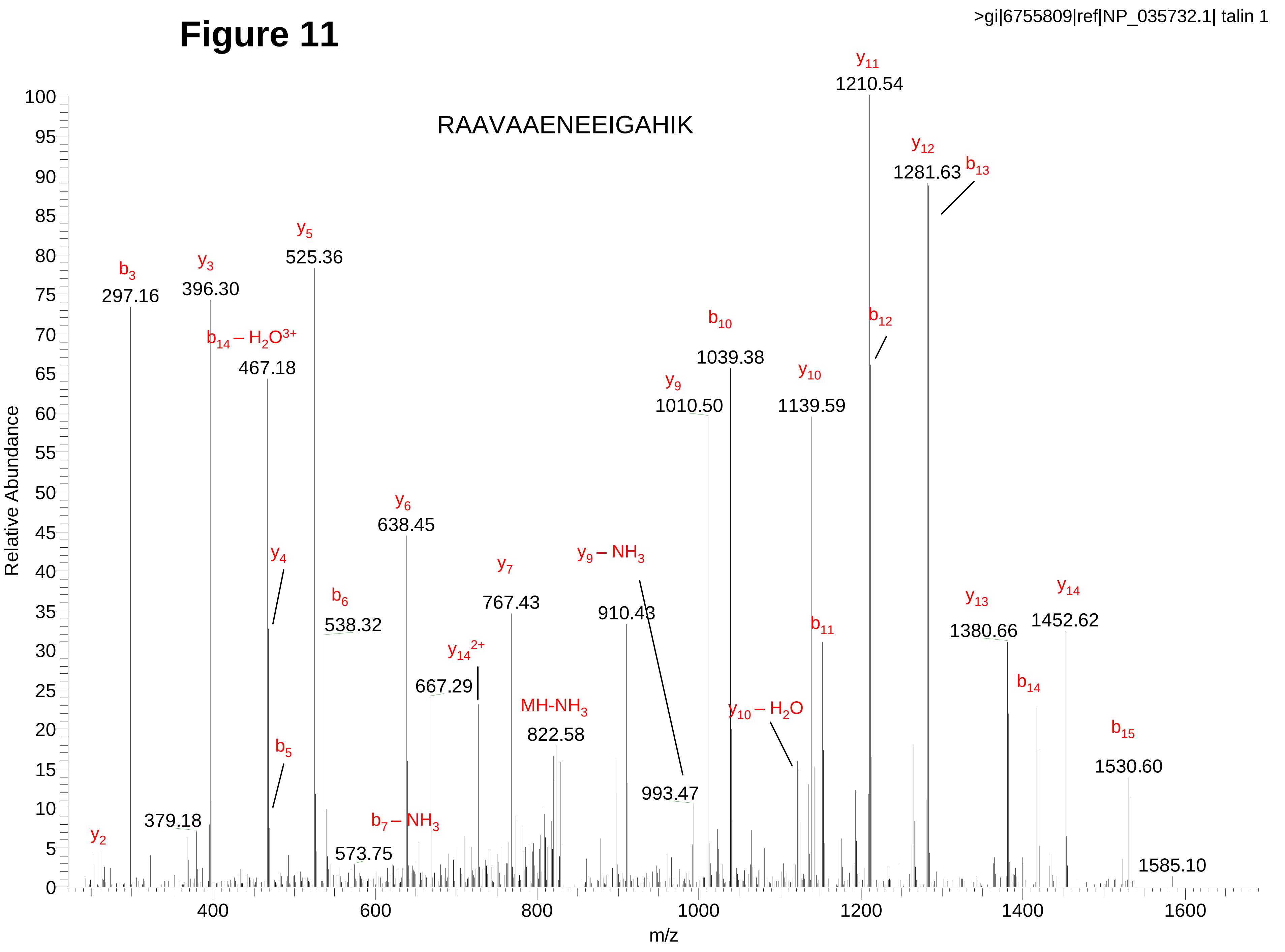

>gi|6755809|ref|NP_035732.1| talin 1
RAAVAAENEEIGAHIK
Relative Abundance
m/z
y11 1210.54
y12 1281.63
b13
y5 525.36
b3 297.16
y3 396.30
b14 – H2O3+ 467.18
b10 1039.38
b12
y9 1010.50
y10 1139.59
y6 638.45
y4
y7 767.43
y9 – NH3 910.43
b6 538.32
b11
y13 1380.66
y14 1452.62
y14 2+ 667.29
MH-NH3 822.58
y10 – H2O
b14
b15 1530.60
b5
993.47
y2
379.18
b7 – NH3
573.75
1585.10

>gi|55408|emb|CAA39807.1| vimentin

Figure 12

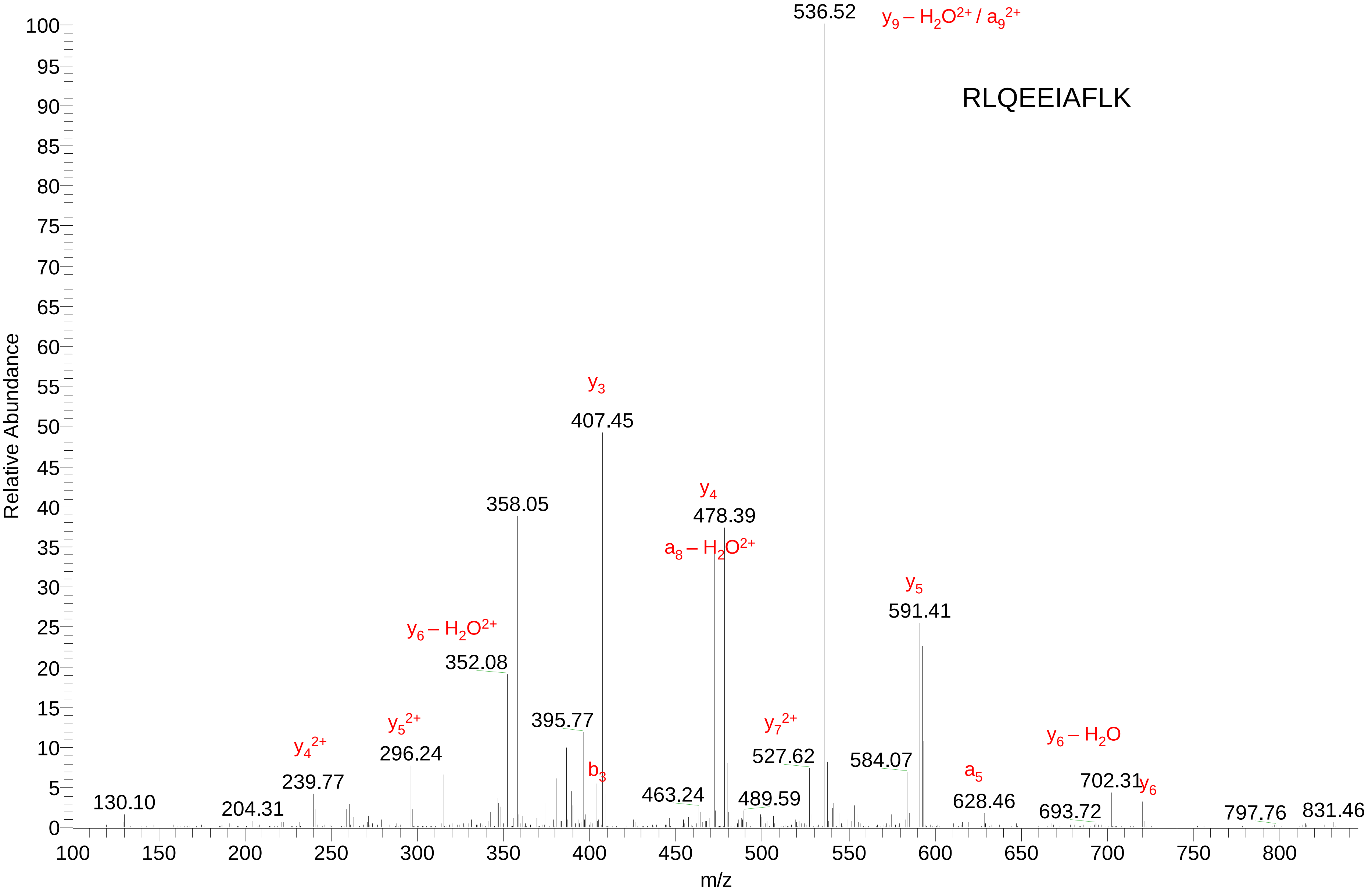

Figure 13

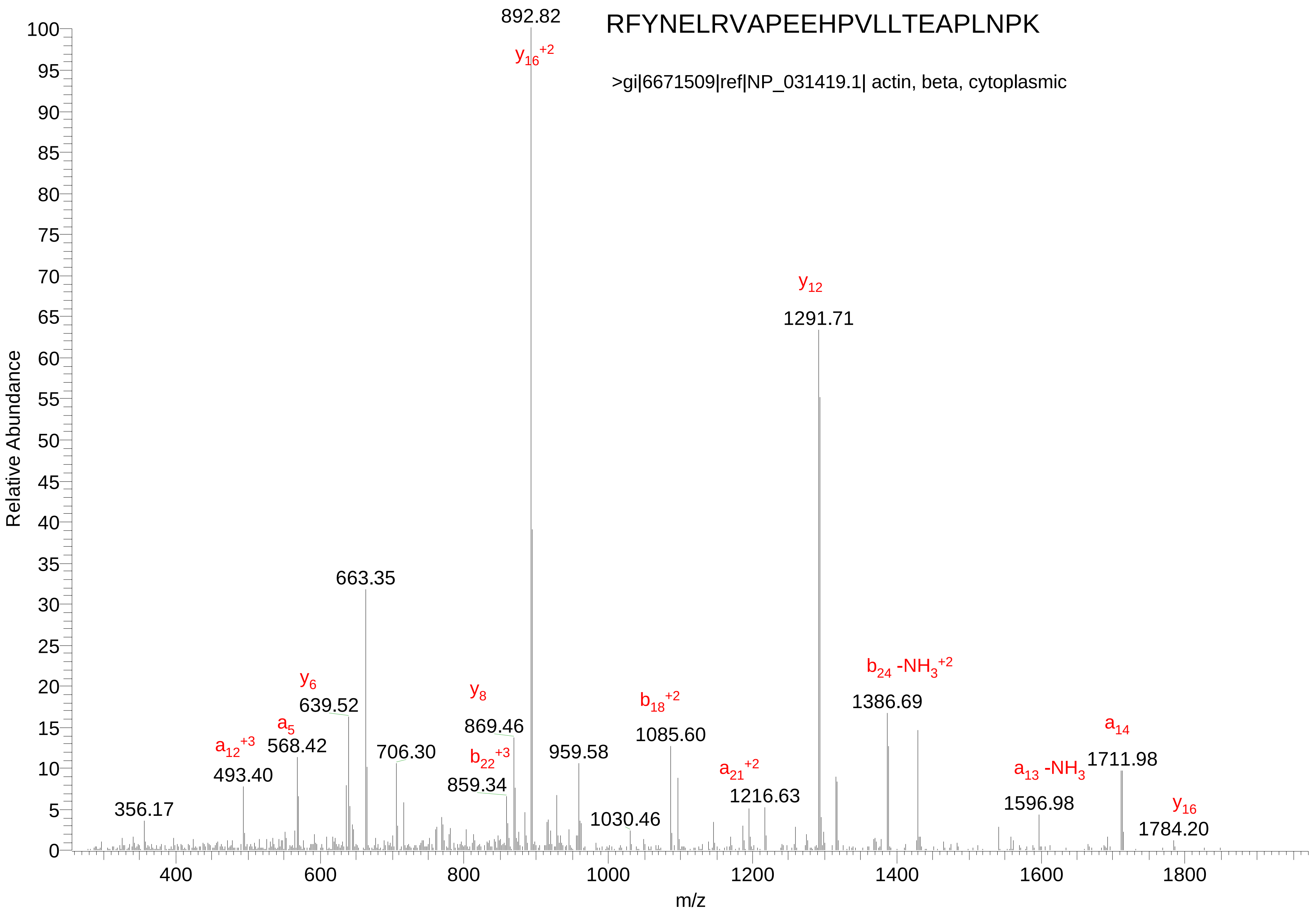
RFYNELRVAPEEHPVLLTEAPLNPK
>gi|6671509|ref|NP_031419.1| actin, beta, cytoplasmic
892.82
y_{16}^{+2}
y_{12}
1291.71
663.35
y_6
639.52
a_5
568.42
a_{12}^{+3}
493.40
356.17
706.30
y_8
869.46
b_{22}^{+3}
859.34
959.58
1030.46
b_{18}^{+2}
1085.60
a_{21}^{+2}
1216.63
b_{24} -NH_3^{+2}
1386.69
a_{13} -NH_3
1596.98
a_{14}
1711.98
y_{16}
1784.20
Relative Abundance
m/z

Figure 14

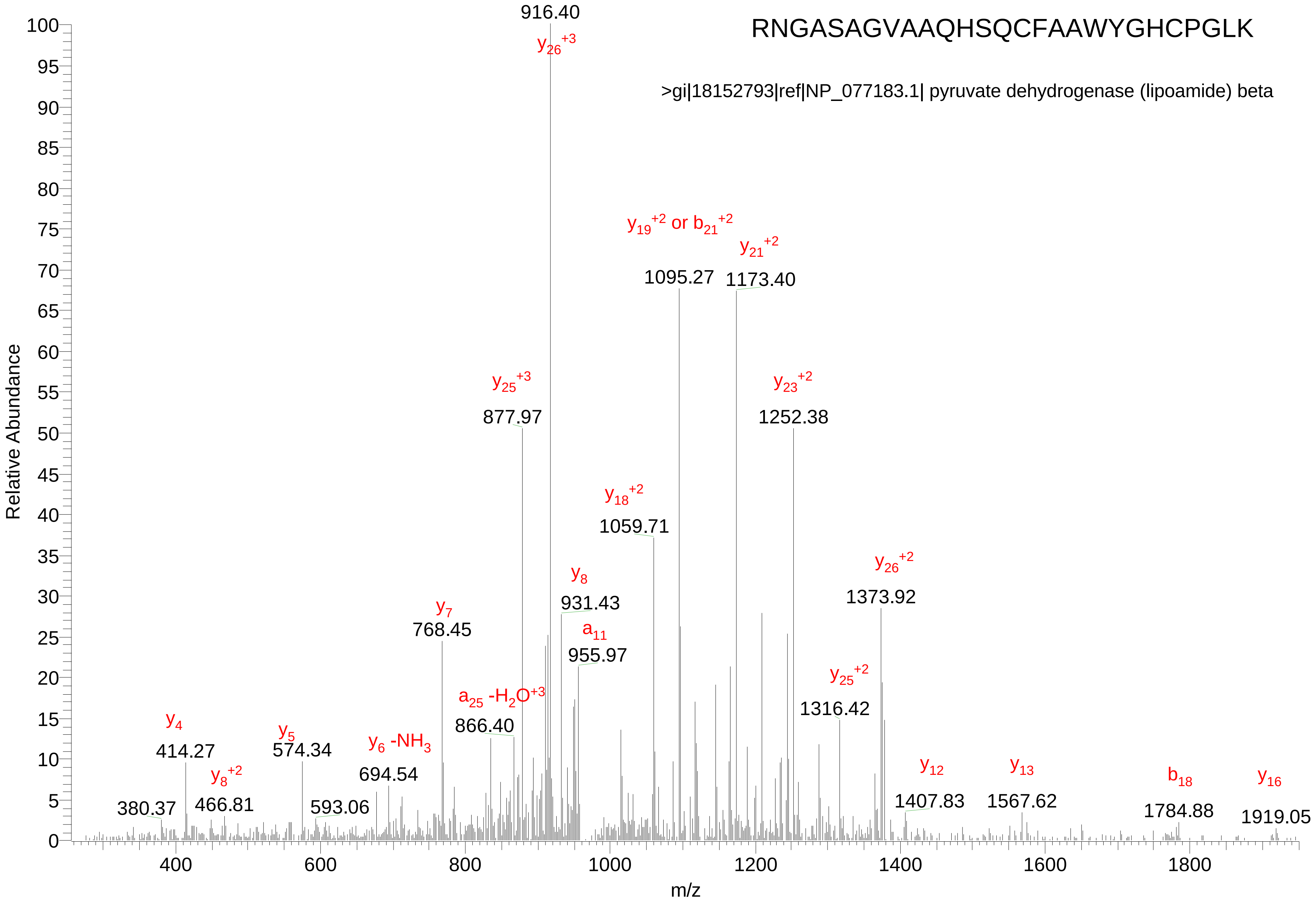

RNGASAGVAAQHSQCFAAWYGHCPGLK
>gi|18152793|ref|NP_077183.1| pyruvate dehydrogenase (lipoamide) beta
Relative Abundance
m/z
916.40
y26+3
y19+2 or b21+2
y21+2
1095.27
1173.40
y25+3
877.97
y23+2
1252.38
y18+2
1059.71
y26+2
1373.92
y8
931.43
y7
768.45
a11
955.97
a25 -H2O+3
866.40
y25+2
1316.42
y4
414.27
y5
574.34
y6 -NH3
694.54
y8+2
466.81
380.37
593.06
y12
1407.83
y13
1567.62
b18
1784.88
y16
1919.05

Figure 15

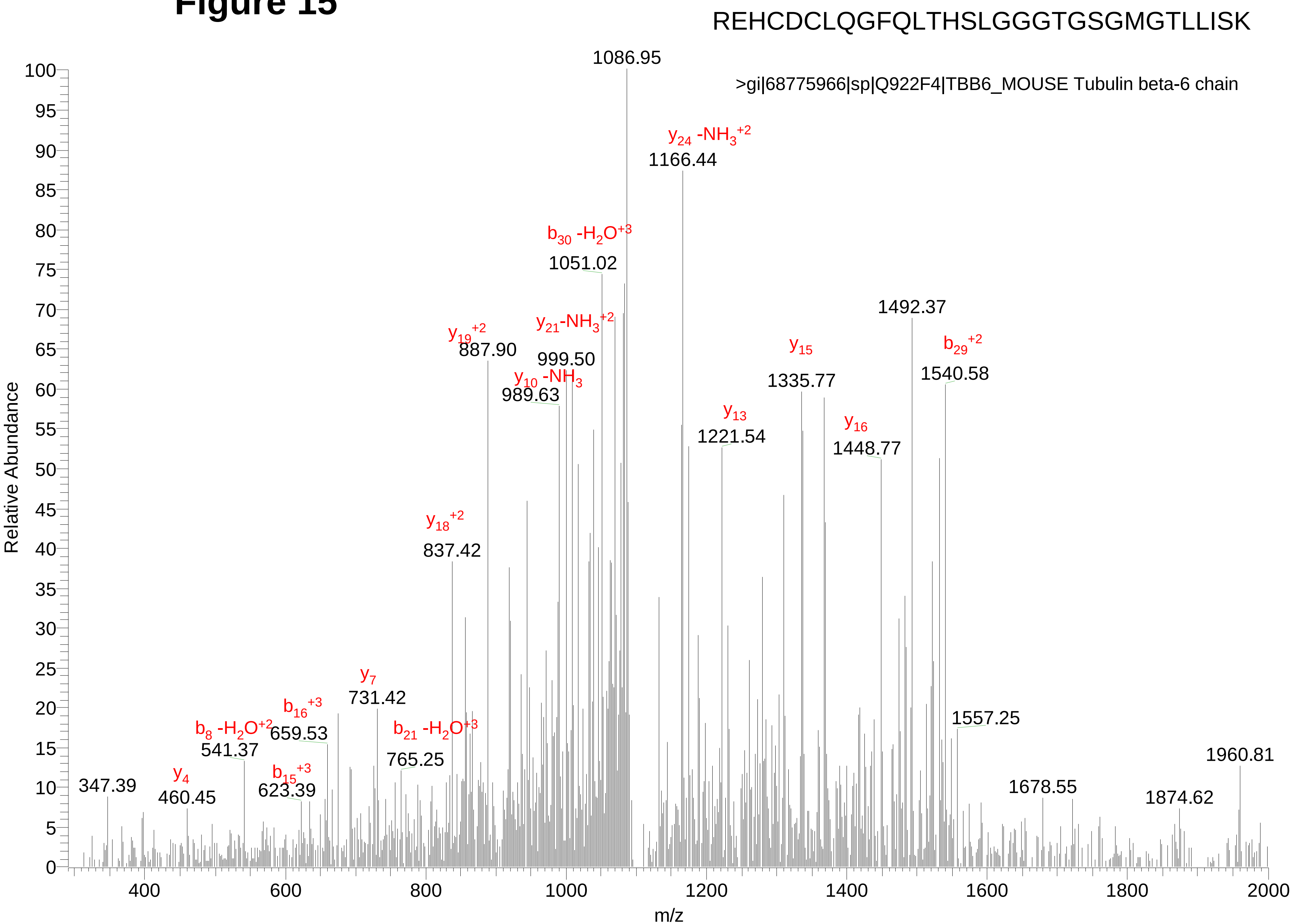

REHCDCLQGFQLTHSLGGGTGSGMGTLLISK
>gi|68775966|sp|Q922F4|TBB6_MOUSE Tubulin beta-6 chain
Relative Abundance
m/z
1086.95
y24 -NH3+2
1166.44
b30 -H2O+3
1051.02
y21-NH3+2
999.50
y19+2
887.90
y10 -NH3
989.63
1492.37
b29+2
1540.58
y15
1335.77
y13
1221.54
y16
1448.77
y18+2
837.42
y7
731.42
b16+3
659.53
b8 -H2O+2
541.37
b21 -H2O+3
765.25
1557.25
1960.81
b15+3
623.39
y4
460.45
347.39
1678.55
1874.62

Figure 16

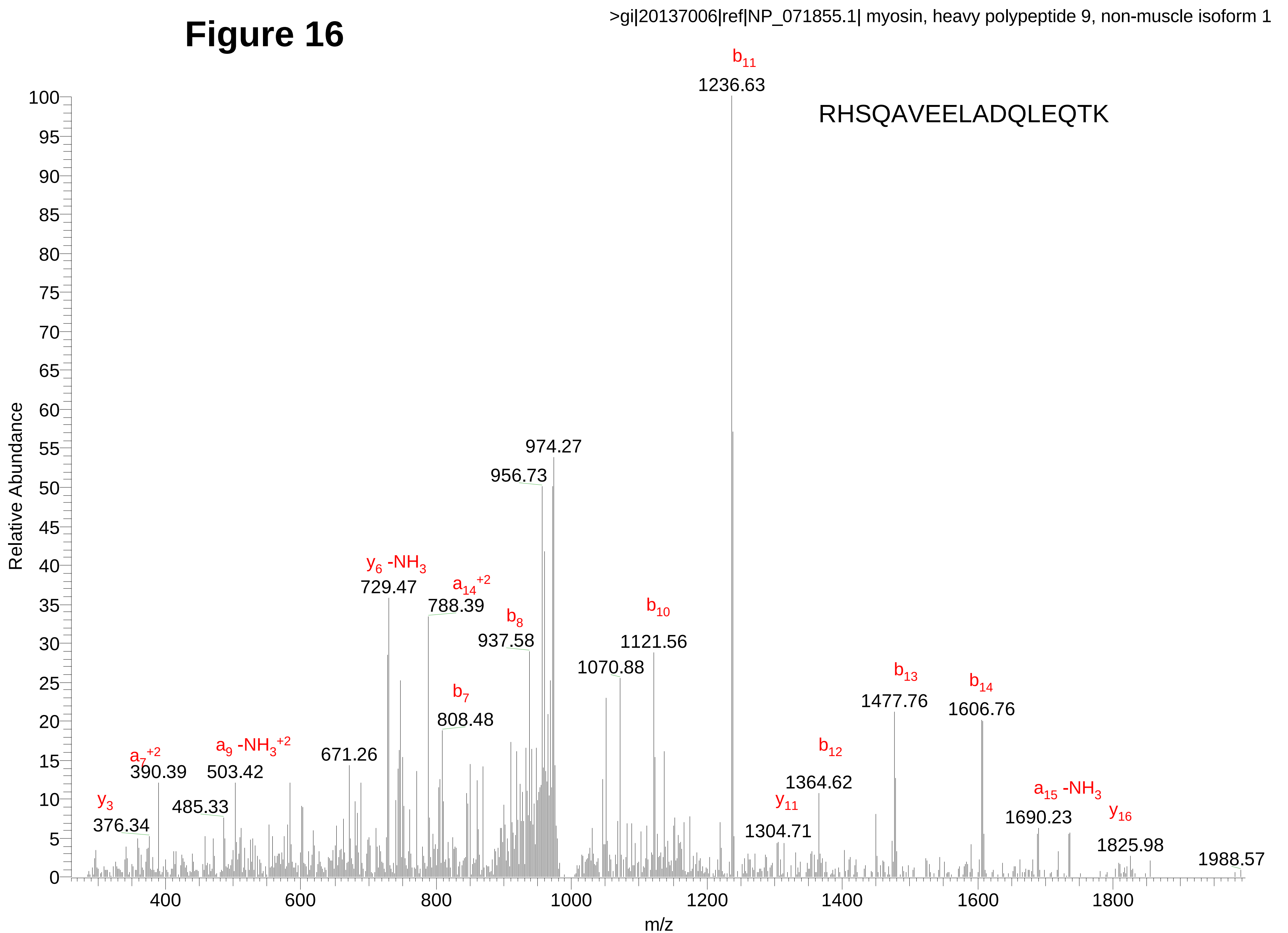

>gi|20137006|ref|NP_071855.1| myosin, heavy polypeptide 9, non-muscle isoform 1
RHSQAVEELADQLEQTK
Relative Abundance
m/z
b11
1236.63
974.27
956.73
y6 -NH3
729.47
a14+2
788.39
b8
937.58
b10
1121.56
1070.88
b13
1477.76
b14
1606.76
b7
808.48
a7+2
390.39
a9 -NH3+2
503.42
671.26
b12
1364.62
y3
376.34
485.33
y11
1304.71
a15 -NH3
1690.23
y16
1825.98
1988.57

Figure 17

>gi|20137006|ref|NP_071855.1| myosin, heavy polypeptide 9, non-muscle isoform 1

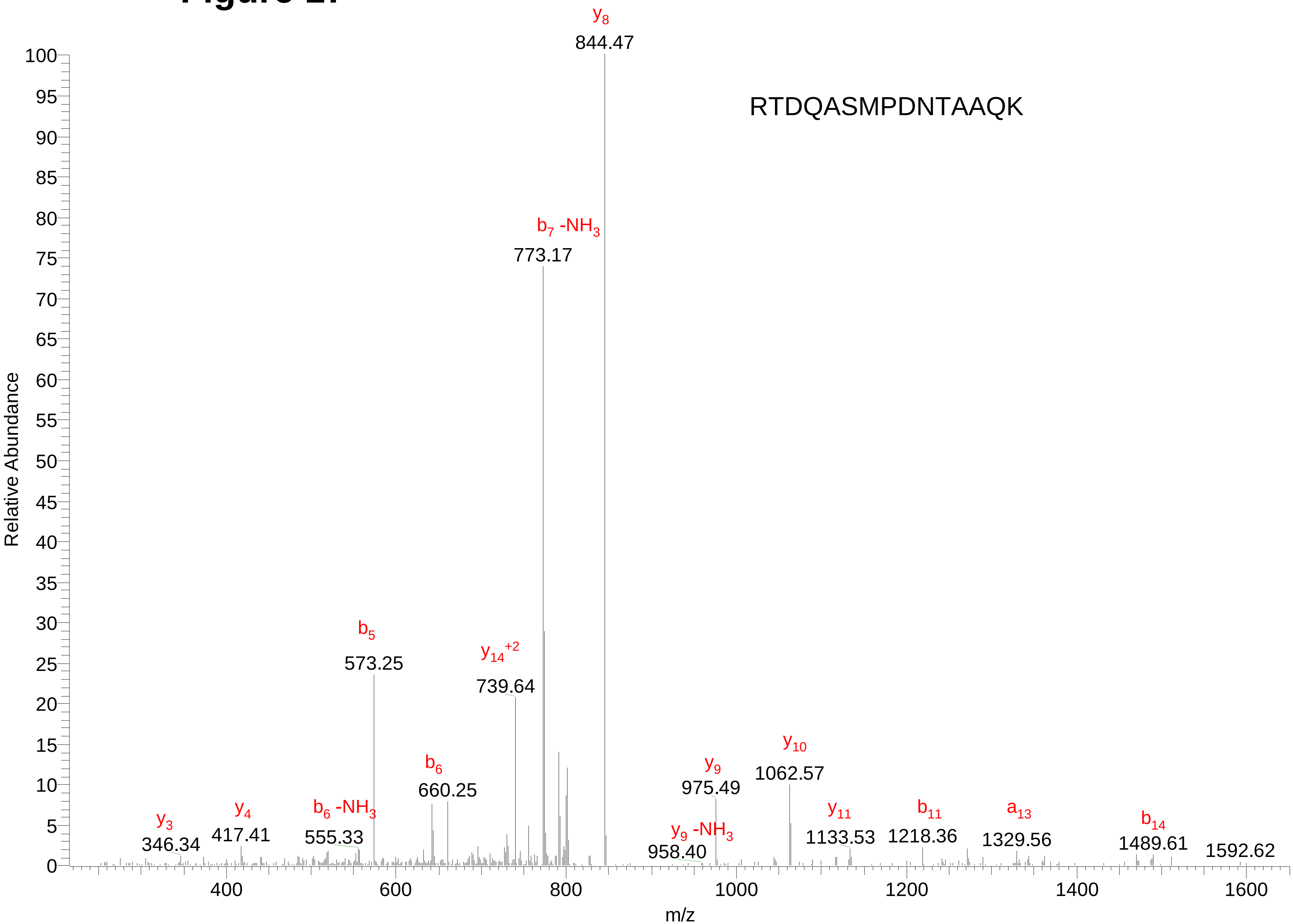

Figure 18

>gi|52345394|ref|NP_001004365.1| ARP3 actin-related protein 3 homolog B

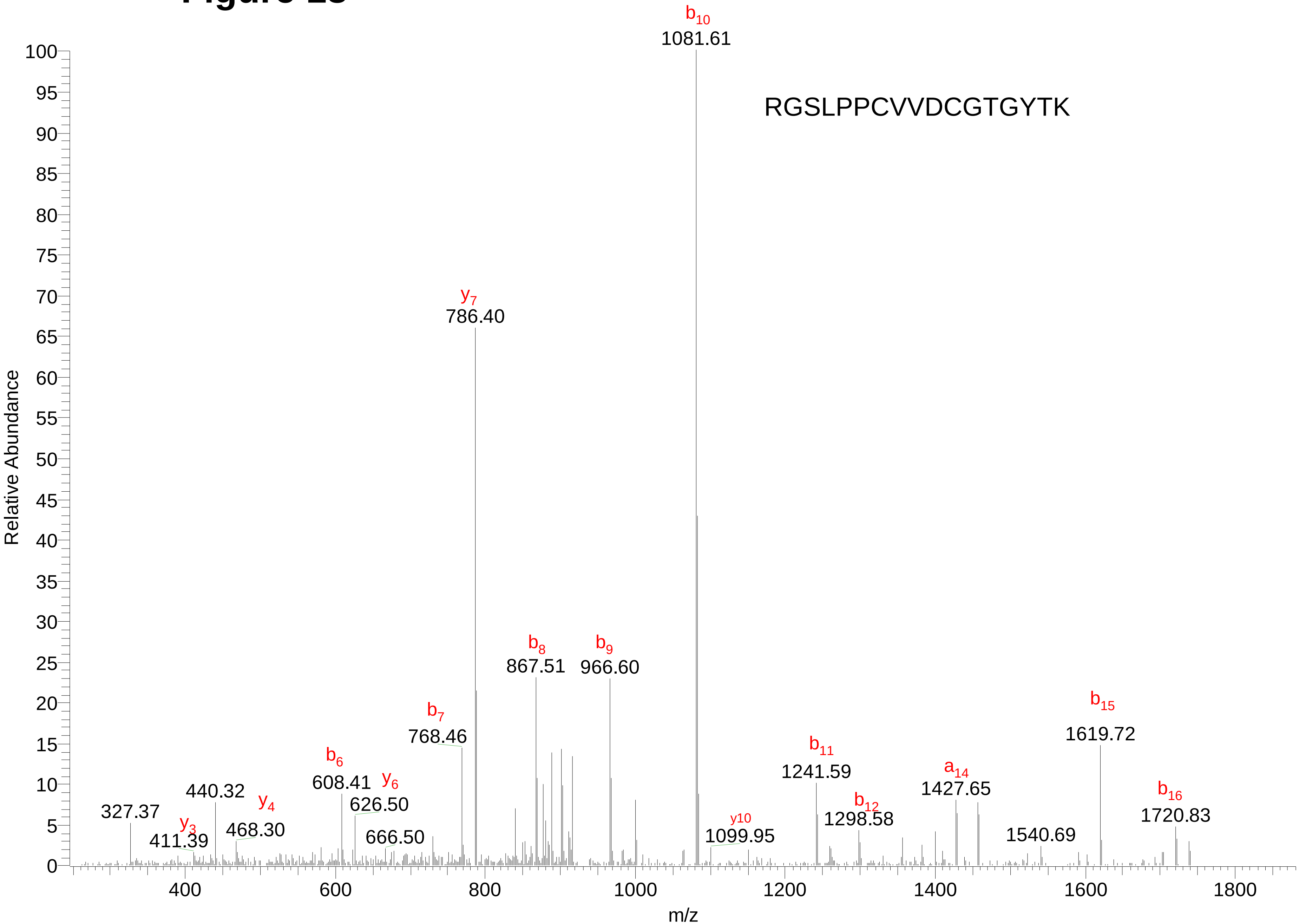

Figure 19

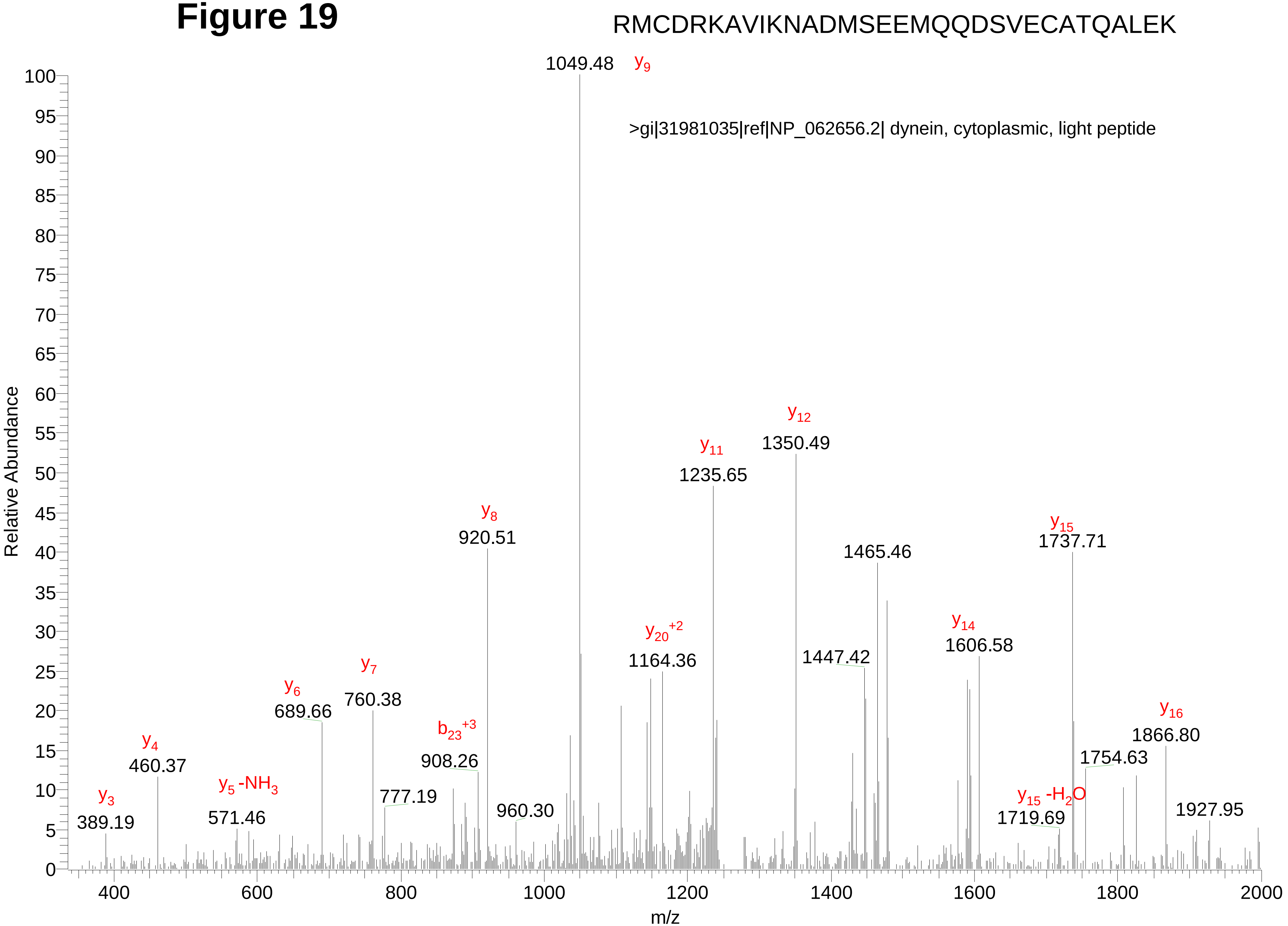
RMCDRKAVIKNADMSEEMQQDSVECATQALEK
>gi|31981035|ref|NP_062656.2| dynein, cytoplasmic, light peptide
1049.48 y9
y12 1350.49
y11 1235.65
y8 920.51
1465.46
y15 1737.71
y20+2 1164.36
1447.42
y14 1606.58
y7 760.38
y6 689.66
b23+3 908.26
y16 1866.80
1754.63
y4 460.37
y5 -NH3 571.46
777.19
960.30
y15 -H2O 1719.69
1927.95
y3 389.19
Relative Abundance
m/z

Figure 20

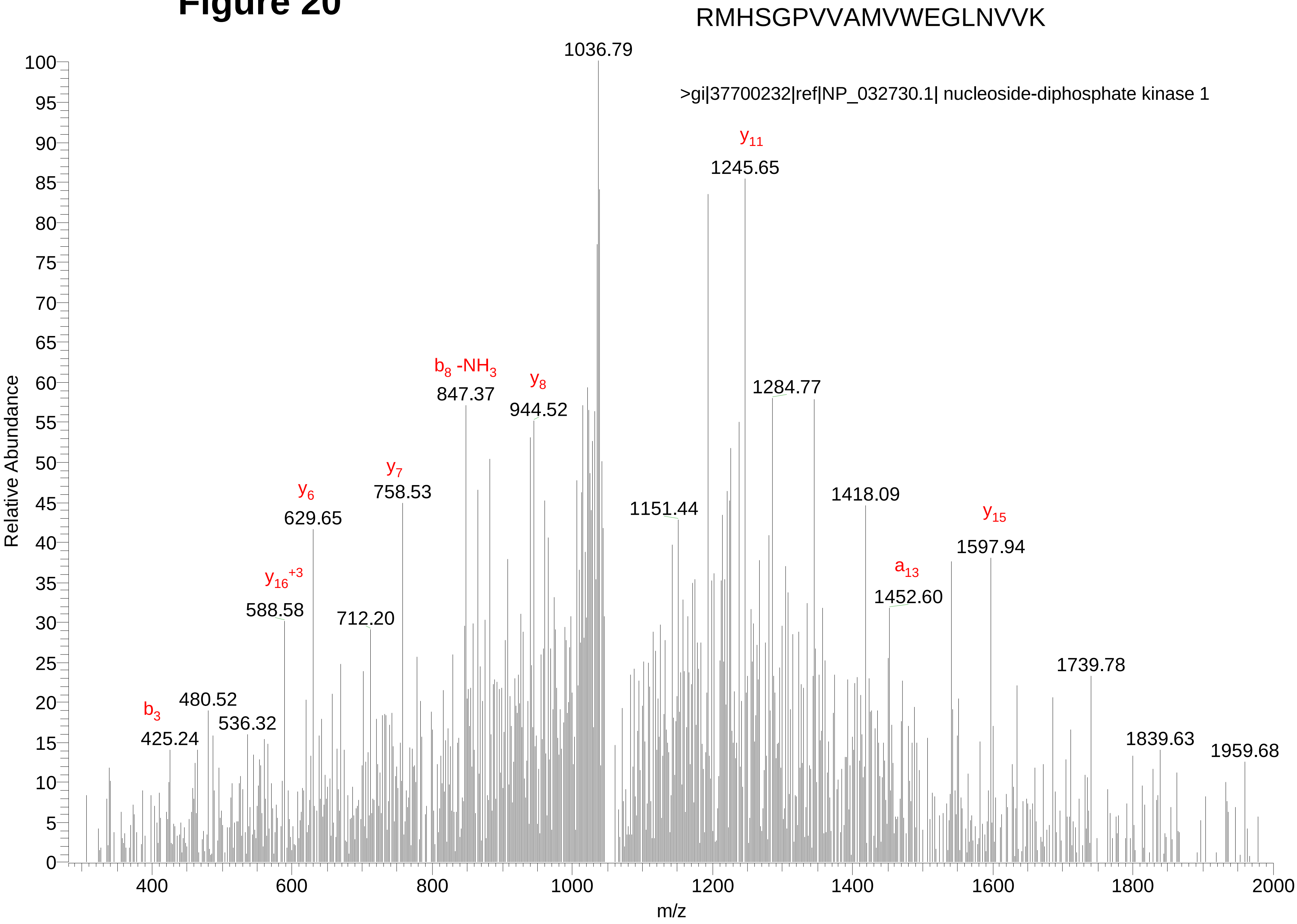
RMHSGPVVAMVWEGLNVVK
>gi|37700232|ref|NP_032730.1| nucleoside-diphosphate kinase 1
1036.79
y11
1245.65
b8 -NH3
847.37
y8
944.52
1284.77
y7
758.53
y6
629.65
1151.44
1418.09
y15
1597.94
y16+3
588.58
712.20
a13
1452.60
1739.78
b3
425.24
480.52
536.32
1839.63
1959.68
Relative Abundance
m/z
100
95
90
85
80
75
70
65
60
55
50
45
40
35
30
25
20
15
10
5
0
400
600
800
1000
1200
1400
1600
1800
2000

Figure 21

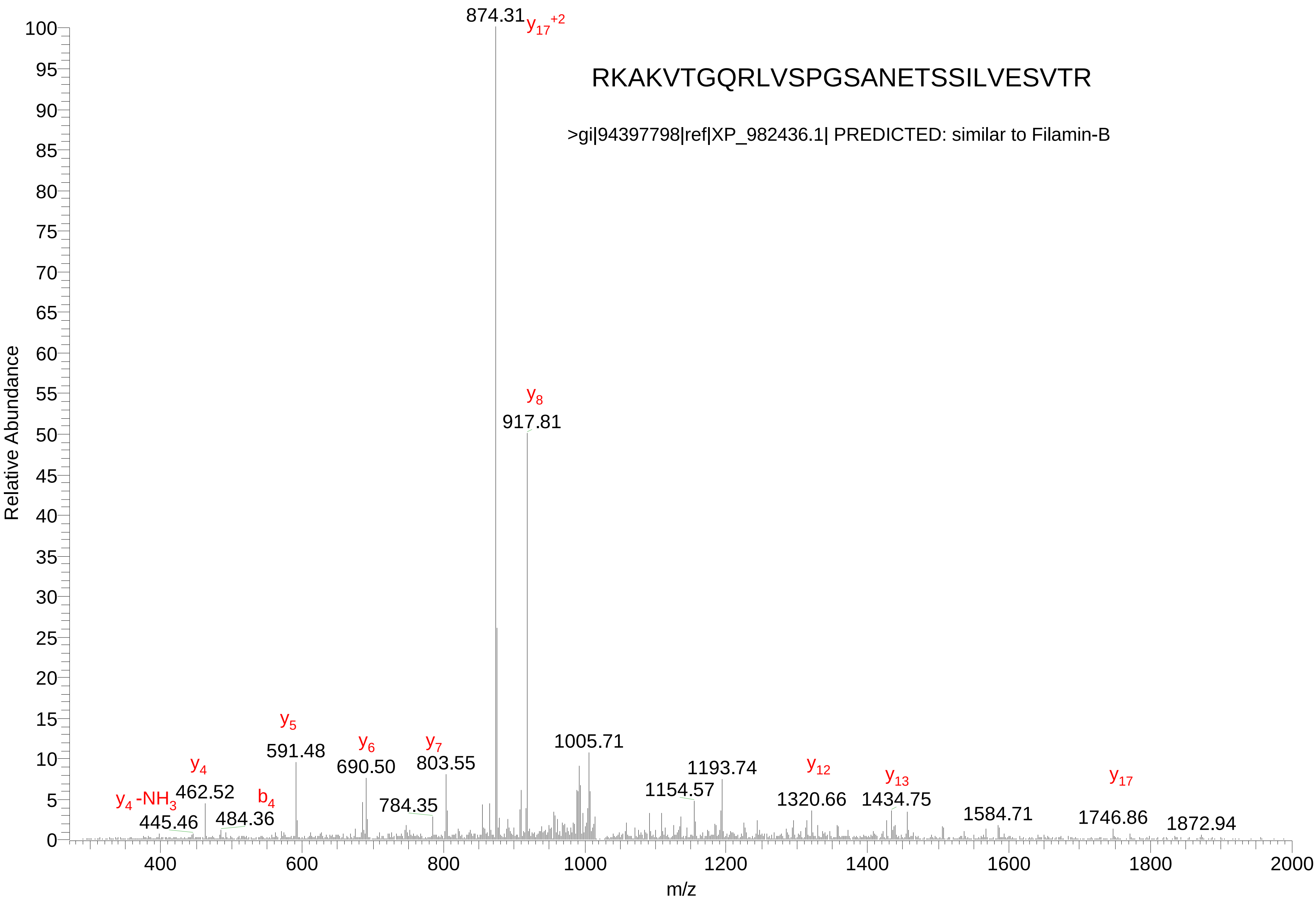

874.31
y17 +2
RKAKVTGQRLVSPGSANETSSILVESVTR
>gi|94397798|ref|XP_982436.1| PREDICTED: similar to Filamin-B
y8
917.81
y5
591.48
y6
690.50
y7
803.55
1005.71
y4
462.52
y4 -NH3
445.46
b4
484.36
784.35
1193.74
1154.57
y12
1320.66
y13
1434.75
1584.71
y17
1746.86
1872.94
Relative Abundance
m/z

Figure 22

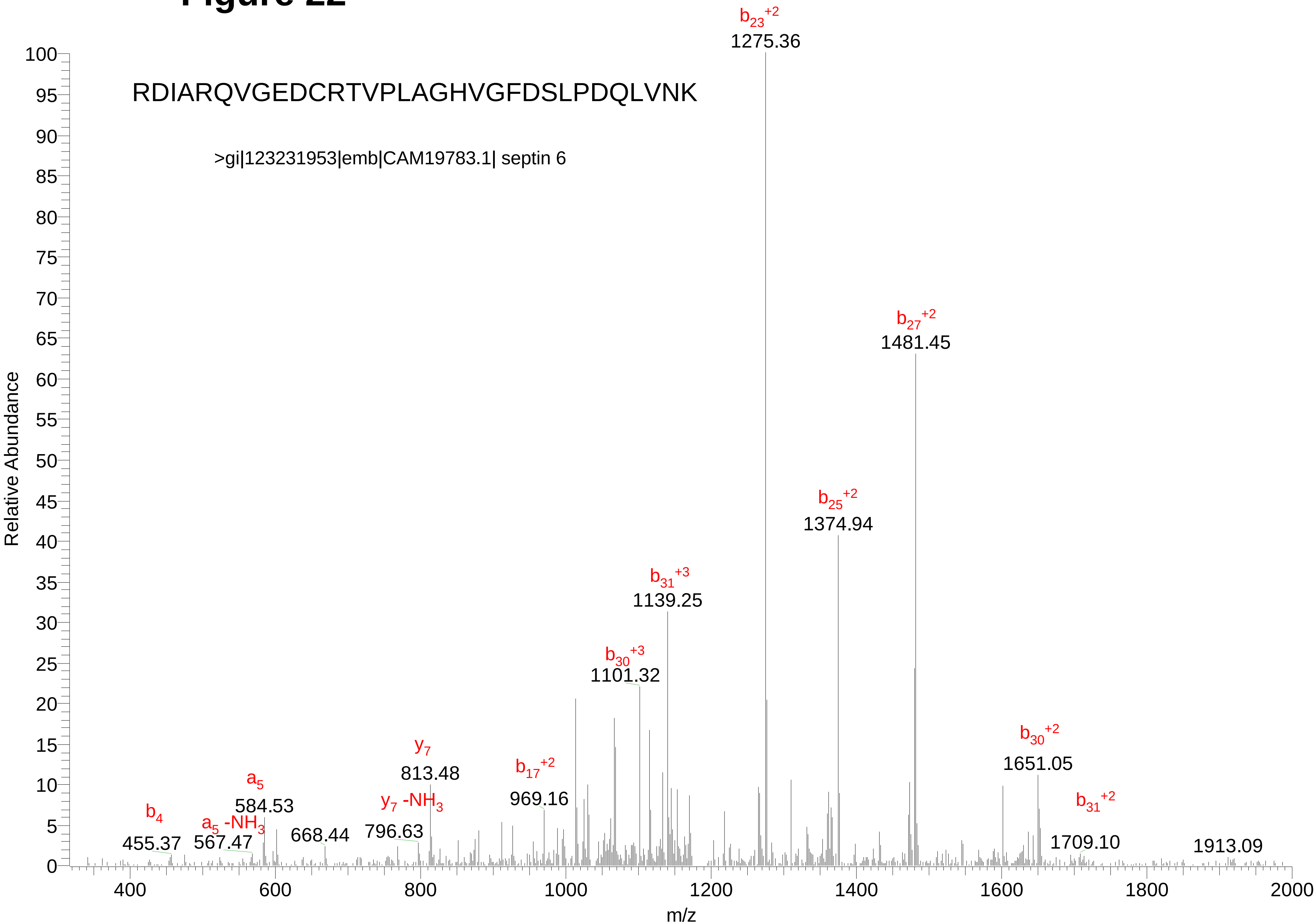

RDIARQVGEDCRTVPLAGHVGFDSLPDQLVNK
>gi|123231953|emb|CAM19783.1| septin 6
b23 +2
1275.36
b27 +2
1481.45
b25 +2
1374.94
b31 +3
1139.25
b30 +3
1101.32
b30 +2
1651.05
b31 +2
1709.10
1913.09
b17 +2
969.16
y7
813.48
y7 -NH3
796.63
668.44
a5
584.53
a5 -NH3
567.47
b4
455.37
Relative Abundance
m/z
0
5
10
15
20
25
30
35
40
45
50
55
60
65
70
75
80
85
90
95
100
400
600
800
1000
1200
1400
1600
1800
2000

Figure 23

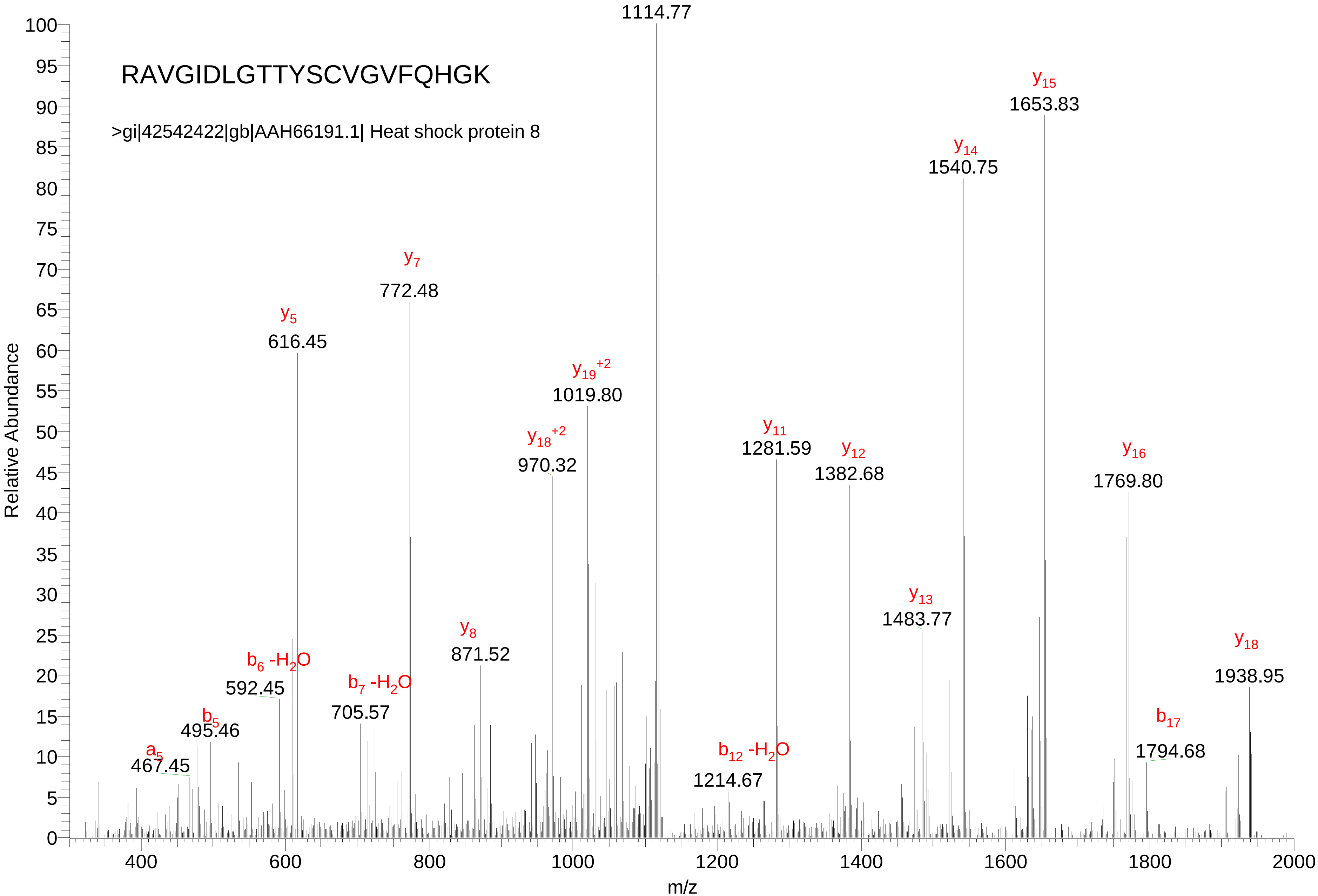

RAVGIDLGTTYSCVGVFQHGK
>gi|42542422|gb|AAH66191.1| Heat shock protein 8
1114.77
y15
1653.83
y14
1540.75
y7
772.48
y5
616.45
y19+2
1019.80
y18+2
970.32
y11
1281.59
y12
1382.68
y16
1769.80
y13
1483.77
y8
871.52
y18
1938.95
b6 -H2O
592.45
b7 -H2O
705.57
b5
495.46
a5
467.45
b12 -H2O
1214.67
b17
1794.68
Relative Abundance
m/z

Figure 24

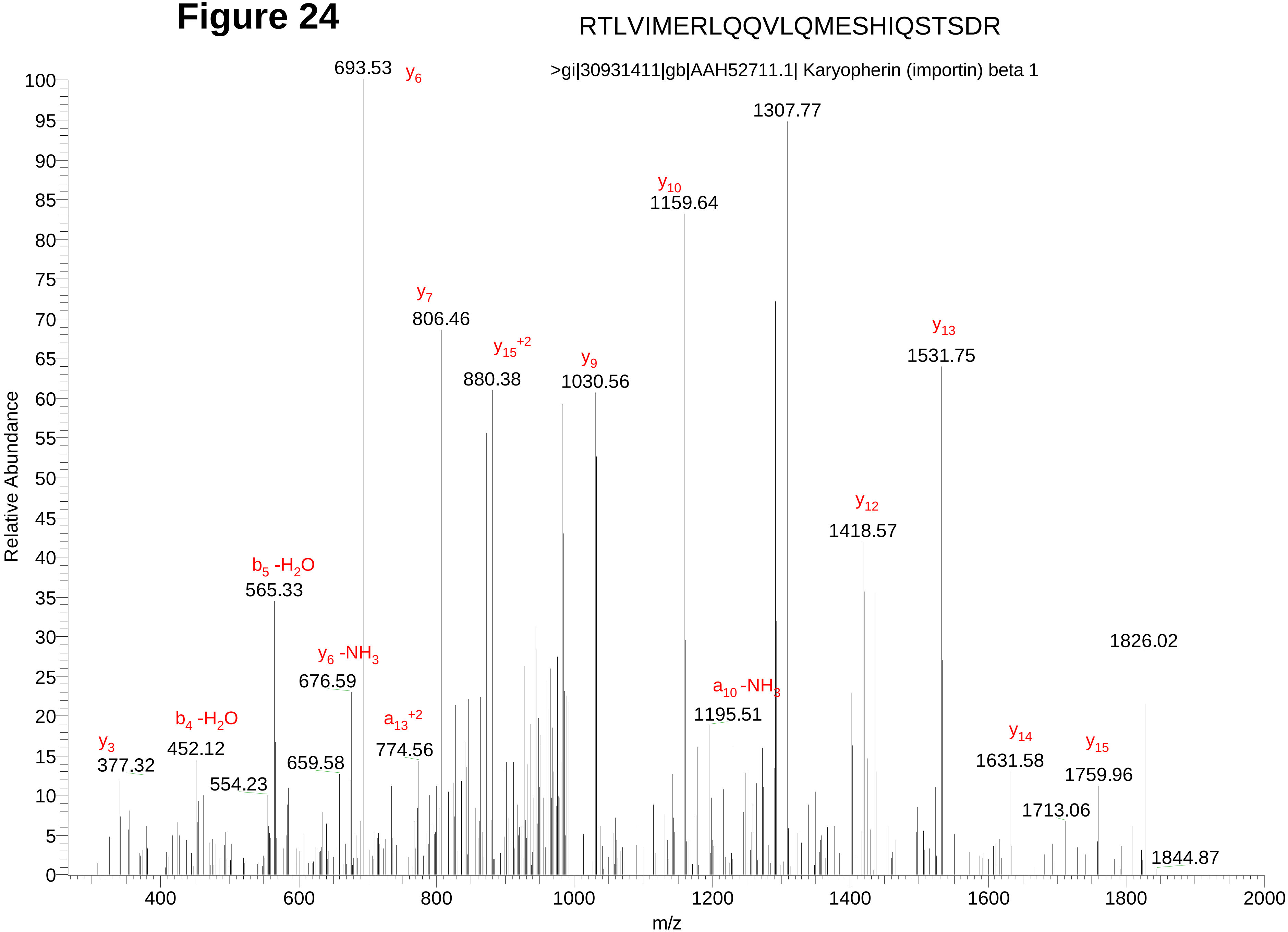
RTLVIMERLQQVLQMESHIQSTSDR
>gi|30931411|gb|AAH52711.1| Karyopherin (importin) beta 1
Relative Abundance
m/z
693.53 y6
1307.77
y10 1159.64
y7 806.46
y15+2 880.38
y9 1030.56
y13 1531.75
y12 1418.57
b5 -H2O 565.33
y6 -NH3 676.59
1826.02
a10 -NH3 1195.51
y3 377.32
b4 -H2O 452.12
554.23
659.58
a13+2 774.56
y14 1631.58
y15 1759.96
1713.06
1844.87

Figure 25

>gi|10946854|ref|NP_067438.1| phosphoribosyl pyrophosphate synthetase 1

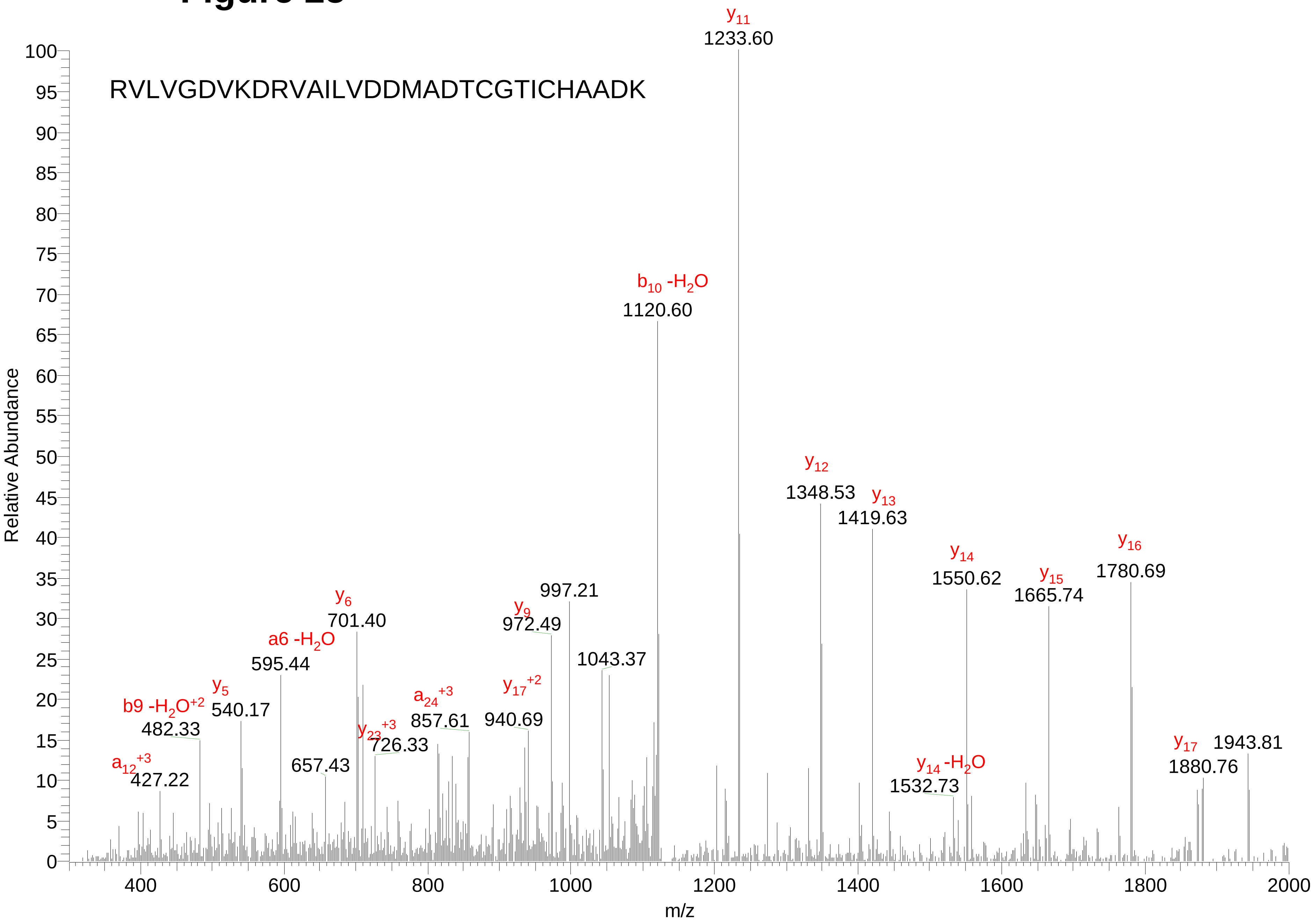

Figure 26

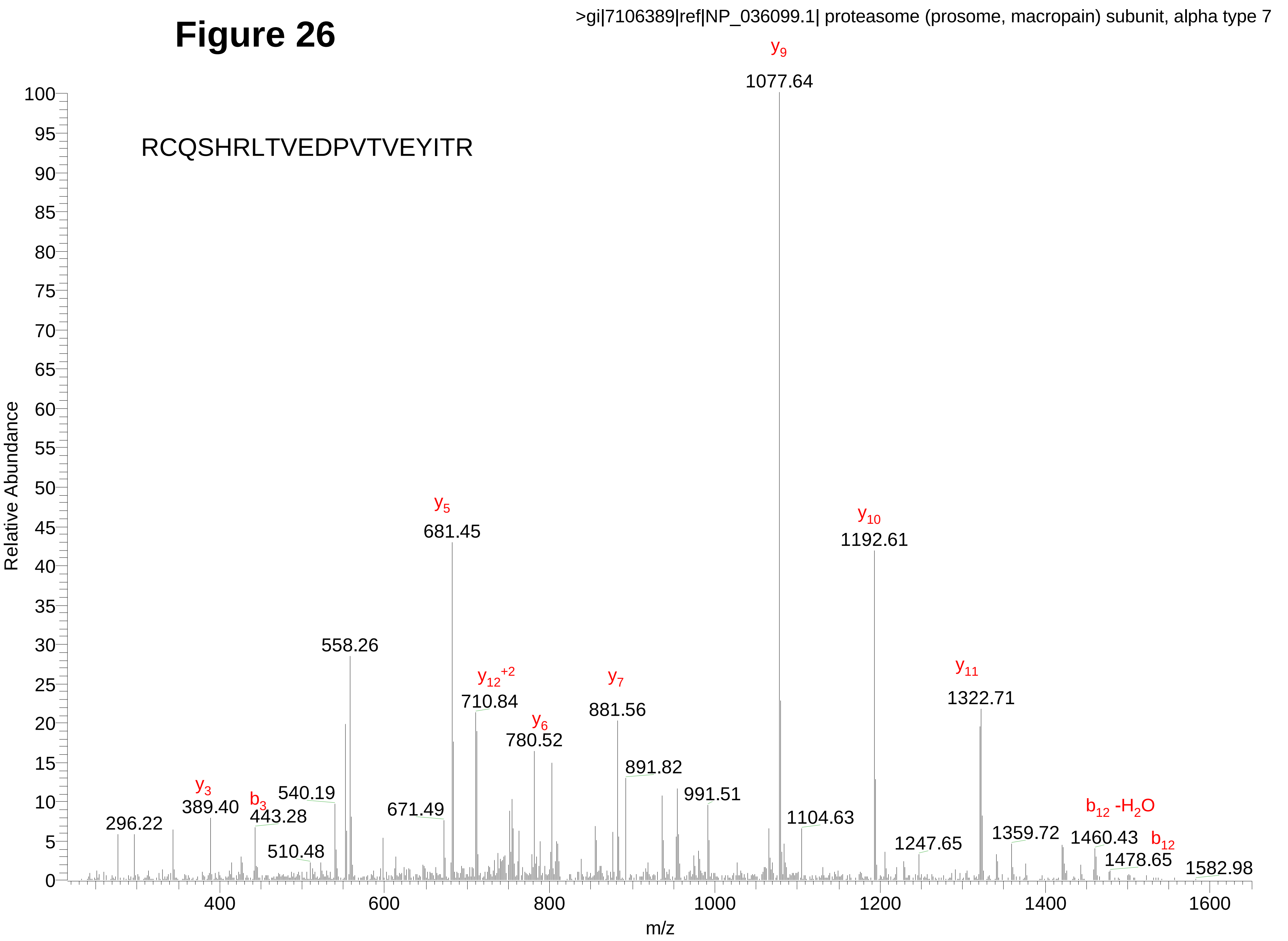

>gi|7106389|ref|NP_036099.1| proteasome (prosome, macropain) subunit, alpha type 7
RCQSHRLTVEDPVTVEYITR
Relative Abundance
m/z
y9
1077.64
y5
681.45
y10
1192.61
558.26
y12+2
710.84
y7
881.56
y11
1322.71
y6
780.52
891.82
991.51
y3
389.40
b3
443.28
540.19
671.49
296.22
510.48
1104.63
1247.65
1359.72
1460.43
b12 -H2O
b12
1478.65
1582.98

Figure 27

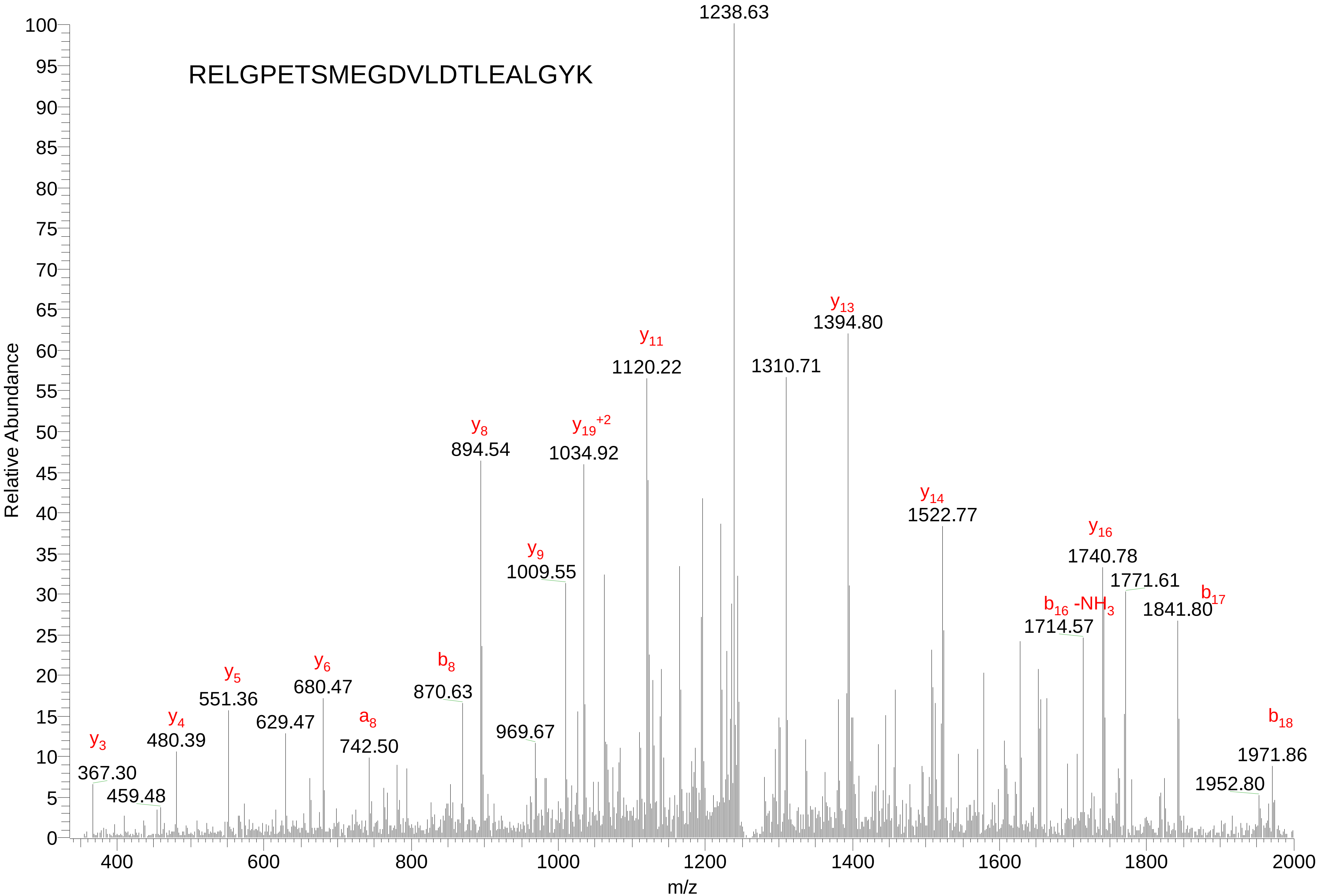

>gi|61098124|ref|NP_080896.2| family with sequence similarity 98, member B
RELGPETSMEGDVLDTLEALGYK
Relative Abundance
m/z
1238.63
y13 1394.80
y11 1120.22
1310.71
y8 894.54
y19+2 1034.92
y14 1522.77
y16 1740.78
y9 1009.55
1771.61
b17 1841.80
b16 -NH3 1714.57
y5 551.36
y6 680.47
b8 870.63
y4 480.39
629.47
a8 742.50
969.67
b18 1971.86
y3 367.30
459.48
1952.80

Figure 28

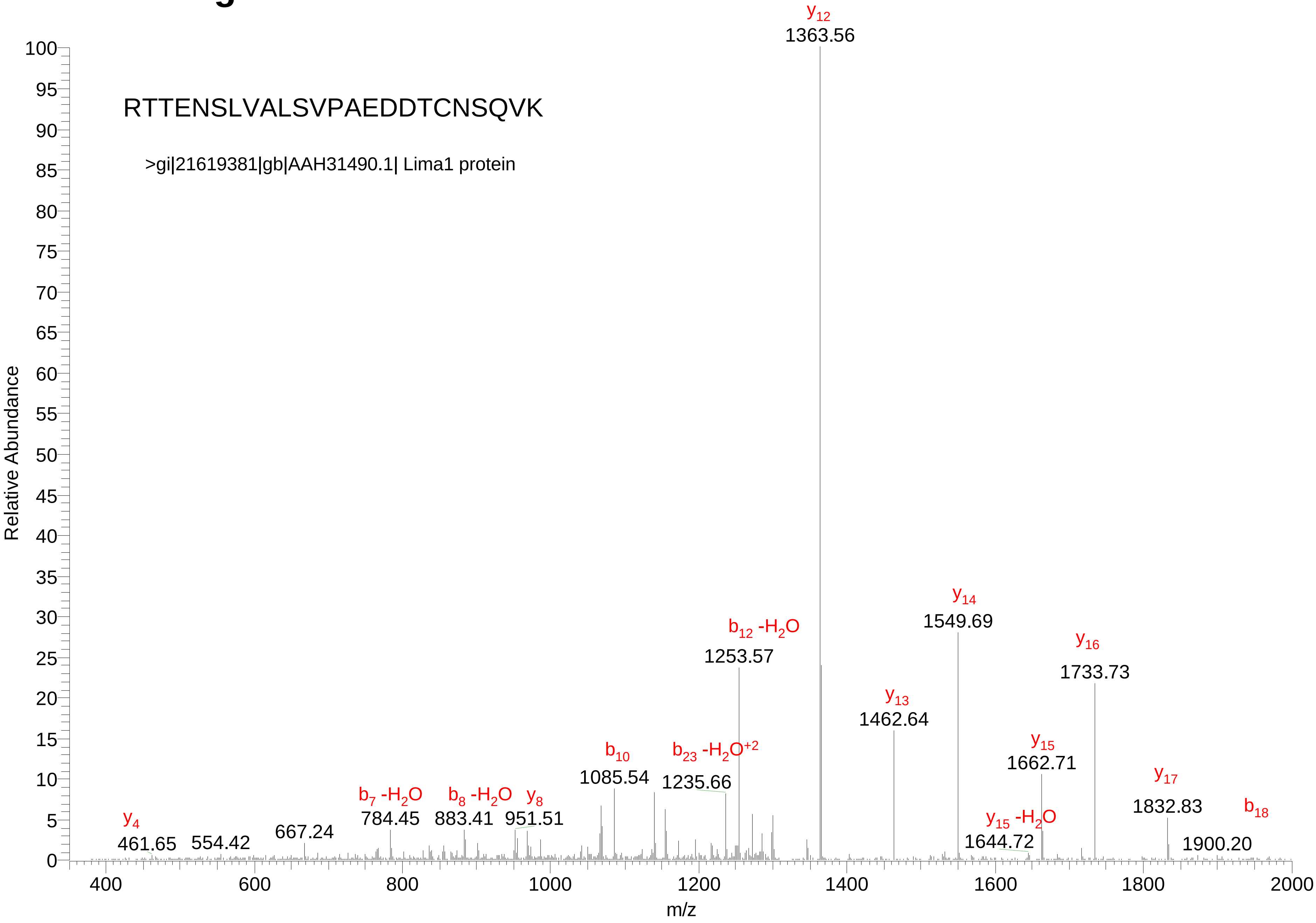
RTTENSLVALSVPAEDDTCNSQVK
>gi|21619381|gb|AAH31490.1| Lima1 protein
Relative Abundance
m/z
y12
1363.56
y14
1549.69
b12 -H2O
1253.57
y16
1733.73
y13
1462.64
y15
1662.71
b10
1085.54
b23 -H2O+2
1235.66
y17
1832.83
b18
1900.20
y15 -H2O
1644.72
b7 -H2O
784.45
b8 -H2O
883.41
y8
951.51
667.24
y4
461.65
554.42

Figure 29

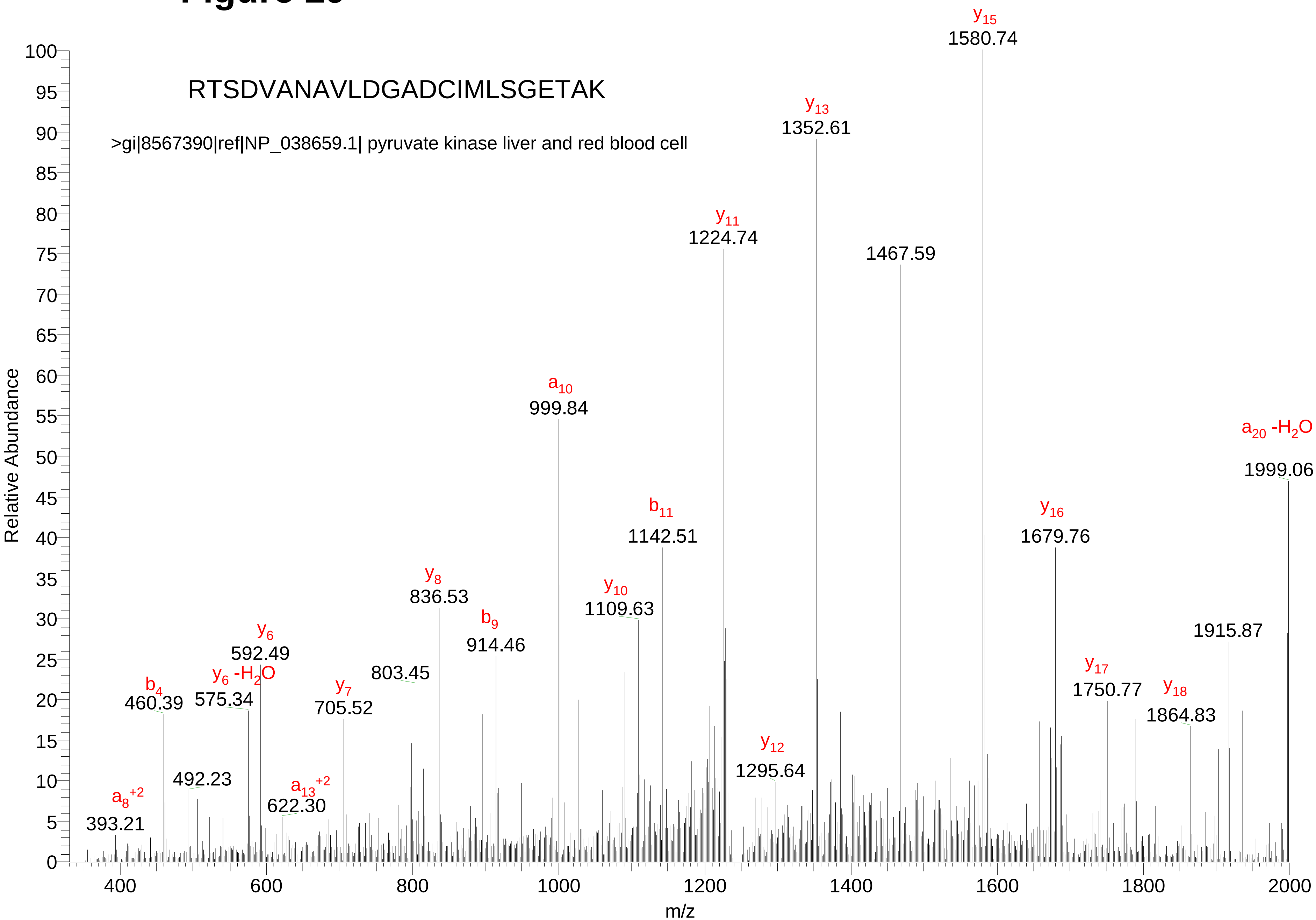
RTSDVANAVLDGADCIMLSGETAK
>gi|8567390|ref|NP_038659.1| pyruvate kinase liver and red blood cell
Relative Abundance
m/z
y15 1580.74
y13 1352.61
y11 1224.74
1467.59
a10 999.84
a20 -H2O 1999.06
b11 1142.51
y16 1679.76
y8 836.53
y10 1109.63
b9 914.46
1915.87
y6 592.49
y6 -H2O 575.34
b4 460.39
803.45
y7 705.52
y17 1750.77
y18 1864.83
y12 1295.64
492.23
a13 +2 622.30
a8 +2 393.21

Figure 30

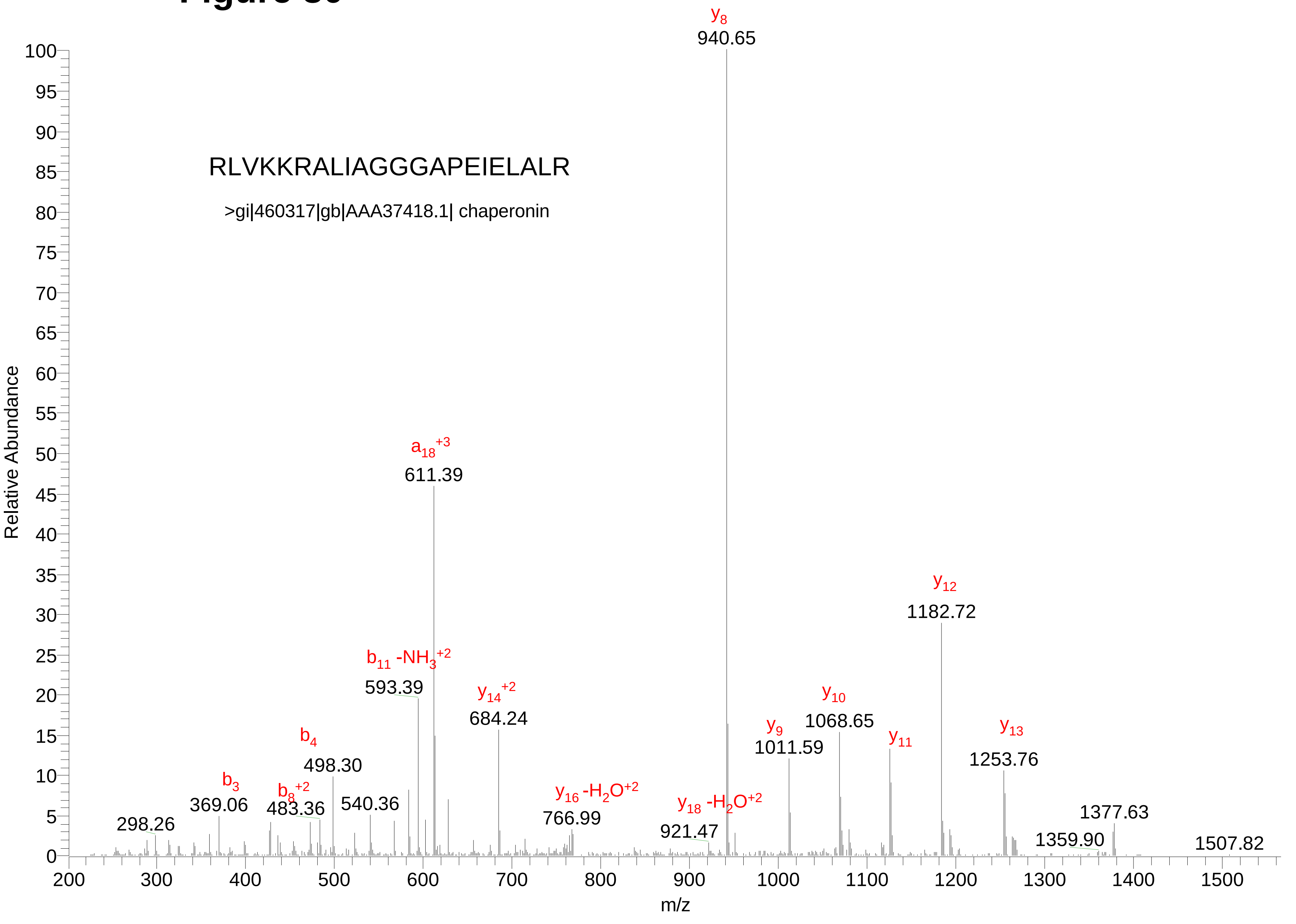

RLVKKRALIAGGGAPEIELALR
>gi|460317|gb|AAA37418.1| chaperonin
Relative Abundance
m/z
y8
940.65
a18 +3
611.39
y12
1182.72
b11 -NH3 +2
593.39
y14 +2
684.24
y10
1068.65
y9
1011.59
y11
y13
1253.76
b4
498.30
b3
369.06
b8 +2
483.36
540.36
y16 -H2O+2
766.99
y18 -H2O+2
921.47
1377.63
1359.90
1507.82
298.26

Figure 31

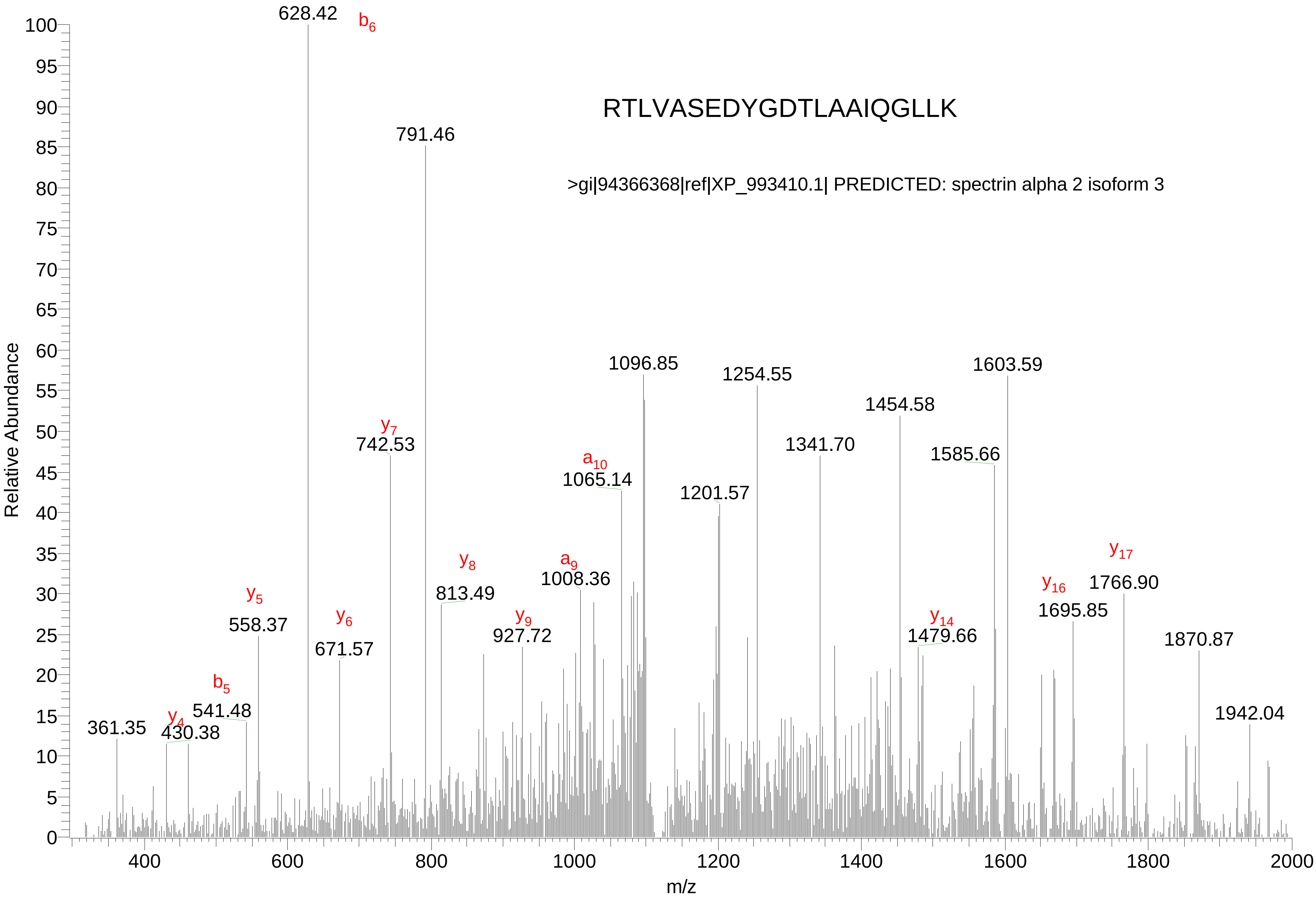

RTLVASEDYGDTLAAIQGLLK
>gi|94366368|ref|XP_993410.1| PREDICTED: spectrin alpha 2 isoform 3
Relative Abundance
m/z
628.42
b6
791.46
y7
742.53
a10
1065.14
1096.85
1254.55
1201.57
1341.70
1454.58
1603.59
1585.66
y8
813.49
a9
1008.36
y9
927.72
y5
558.37
y6
671.57
b5
541.48
y4
430.38
361.35
y14
1479.66
y16
1695.85
y17
1766.90
1870.87
1942.04

Figure 32

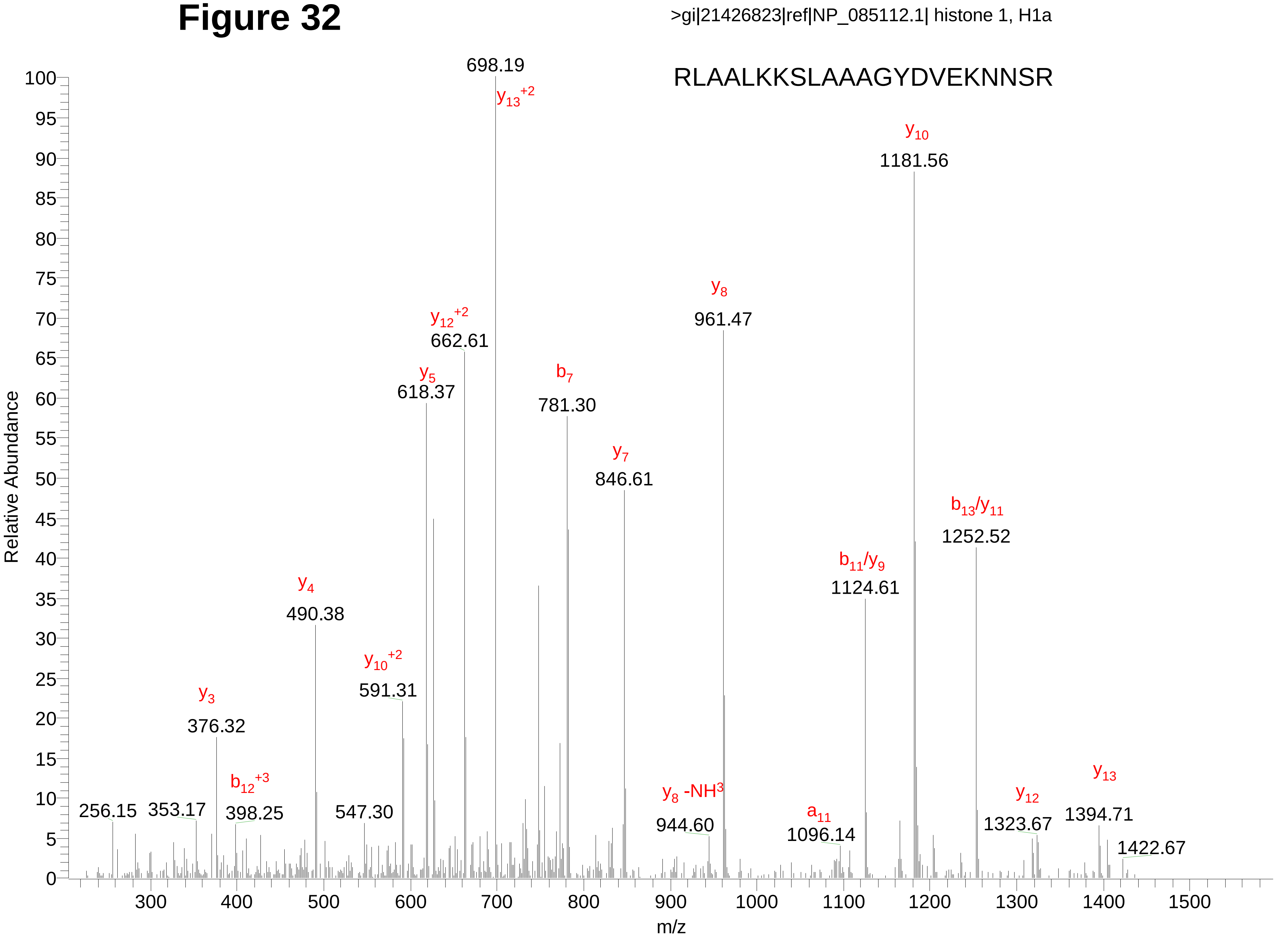
>gi|21426823|ref|NP_085112.1| histone 1, H1a
RLAALKKSLAAAGYDVEKNNSR
Relative Abundance
m/z
698.19
y13+2
y10
1181.56
y8
961.47
y12+2
662.61
y5
618.37
b7
781.30
y7
846.61
b13/y11
1252.52
b11/y9
1124.61
y4
490.38
y10+2
591.31
y3
376.32
b12+3
256.15
353.17
398.25
547.30
y8 -NH3
944.60
a11
1096.14
y12
1323.67
y13
1394.71
1422.67

Figure 33

>gi|111038118|ref|NP_032238.2| L-3-hydroxyacyl-Coenzyme A dehydrogenase

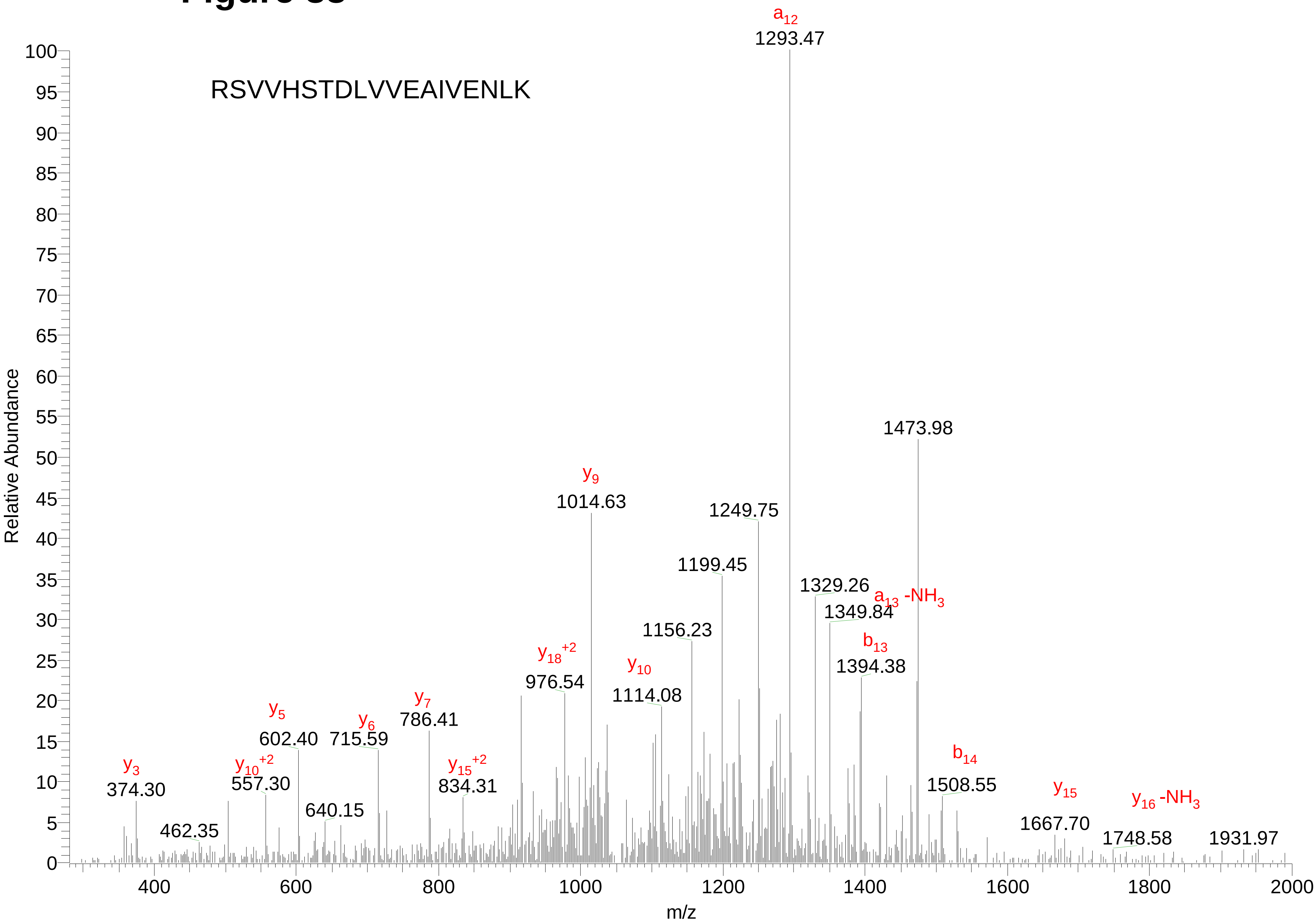

Figure 34

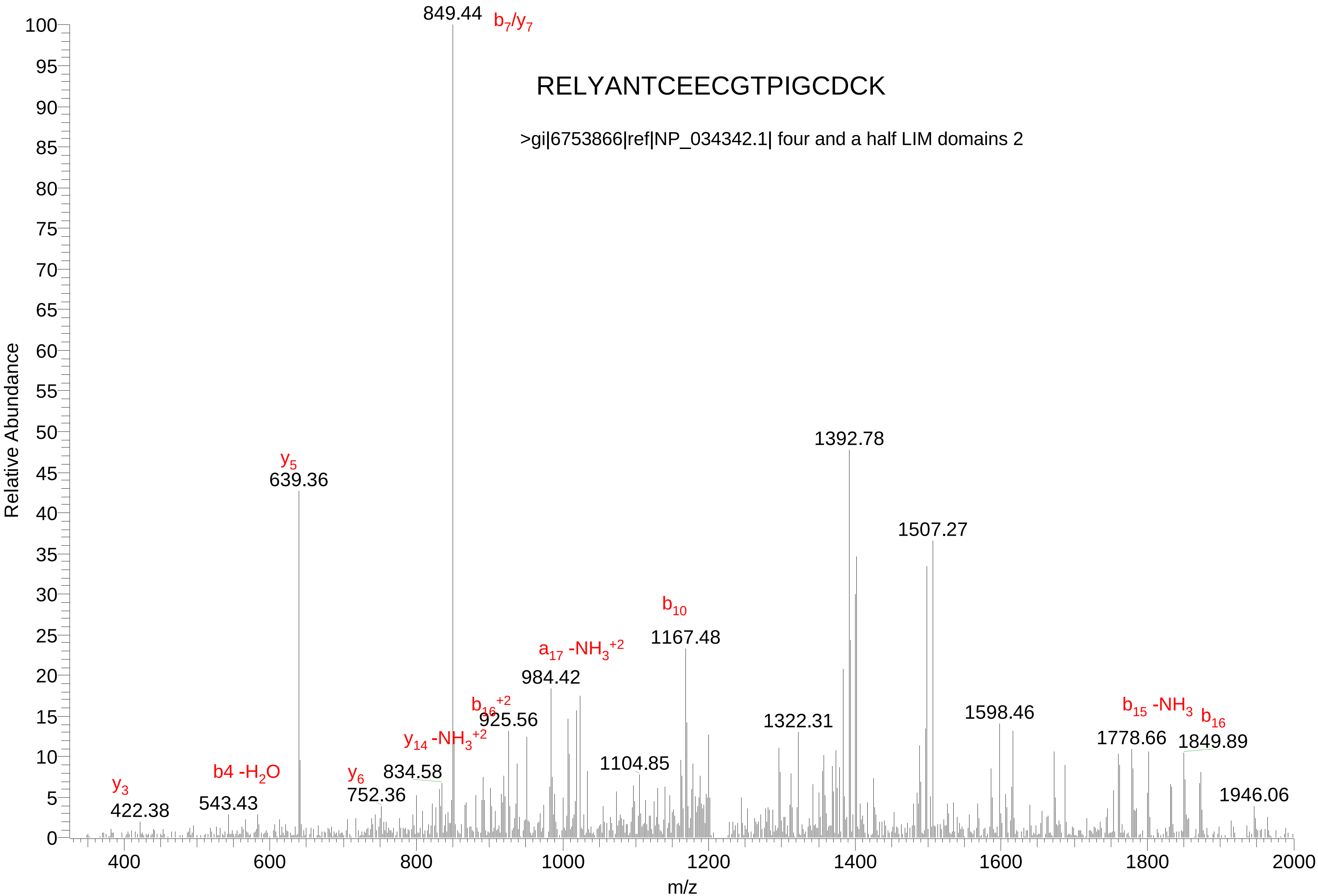

RELYANTCEECGTPIGCDCK
>gi|6753866|ref|NP_034342.1| four and a half LIM domains 2
Relative Abundance
m/z
849.44
b7/y7
y5
639.36
1392.78
1507.27
b10
1167.48
a17 -NH3+2
984.42
b16+2
925.56
y14 -NH3+2
834.58
y6
752.36
b4 -H2O
543.43
y3
422.38
1104.85
1322.31
1598.46
b15 -NH3
b16
1778.66
1849.89
1946.06

Figure 35

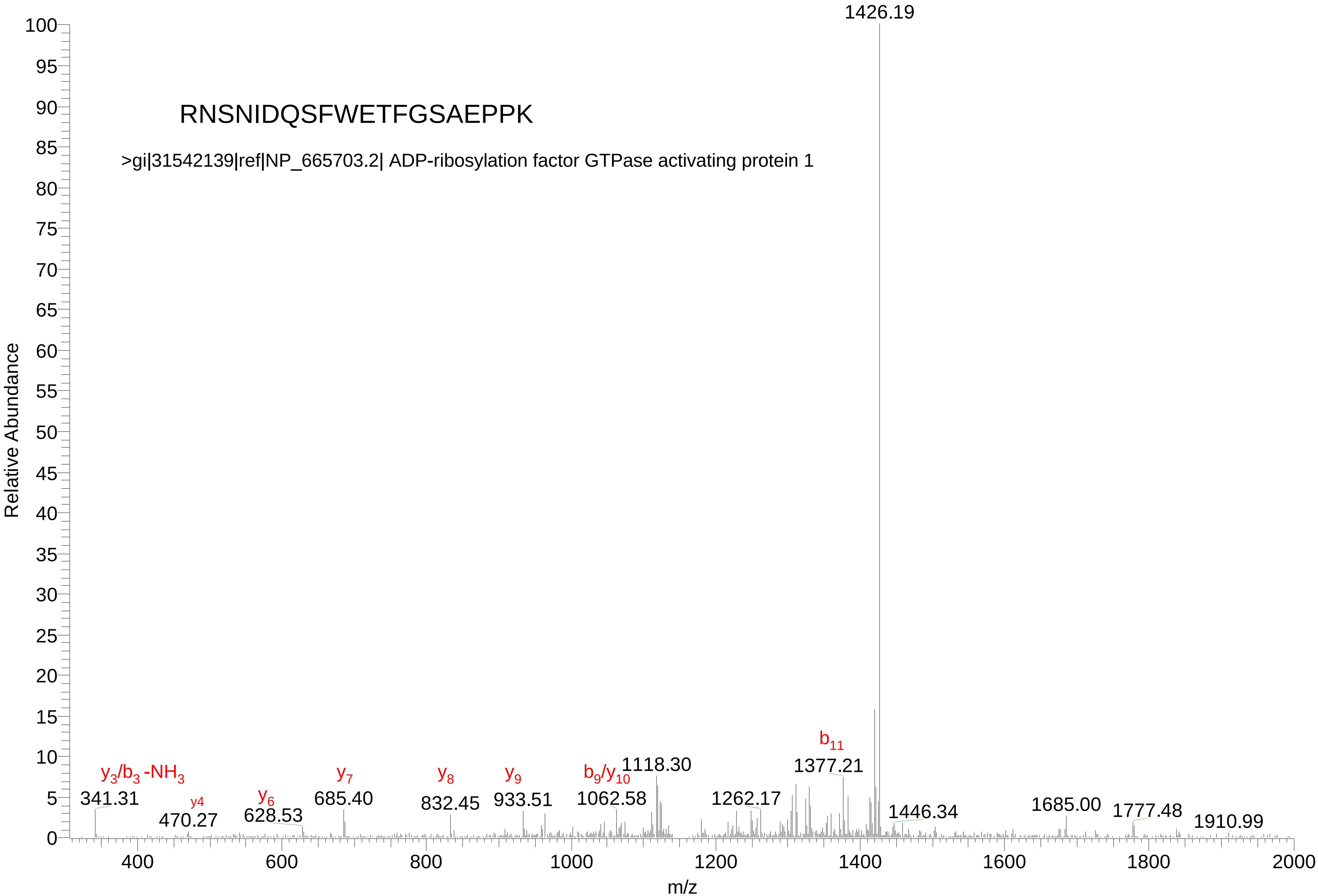

RNSNIDQSFWETFGSAEPPK
>gi|31542139|ref|NP_665703.2| ADP-ribosylation factor GTPase activating protein 1
Relative Abundance
m/z
1426.19
y3/b3 -NH3
341.31
y4
470.27
y6
628.53
y7
685.40
y8
832.45
y9
933.51
b9/y10
1062.58
1118.30
1262.17
b11
1377.21
1446.34
1685.00
1777.48
1910.99

Figure 36

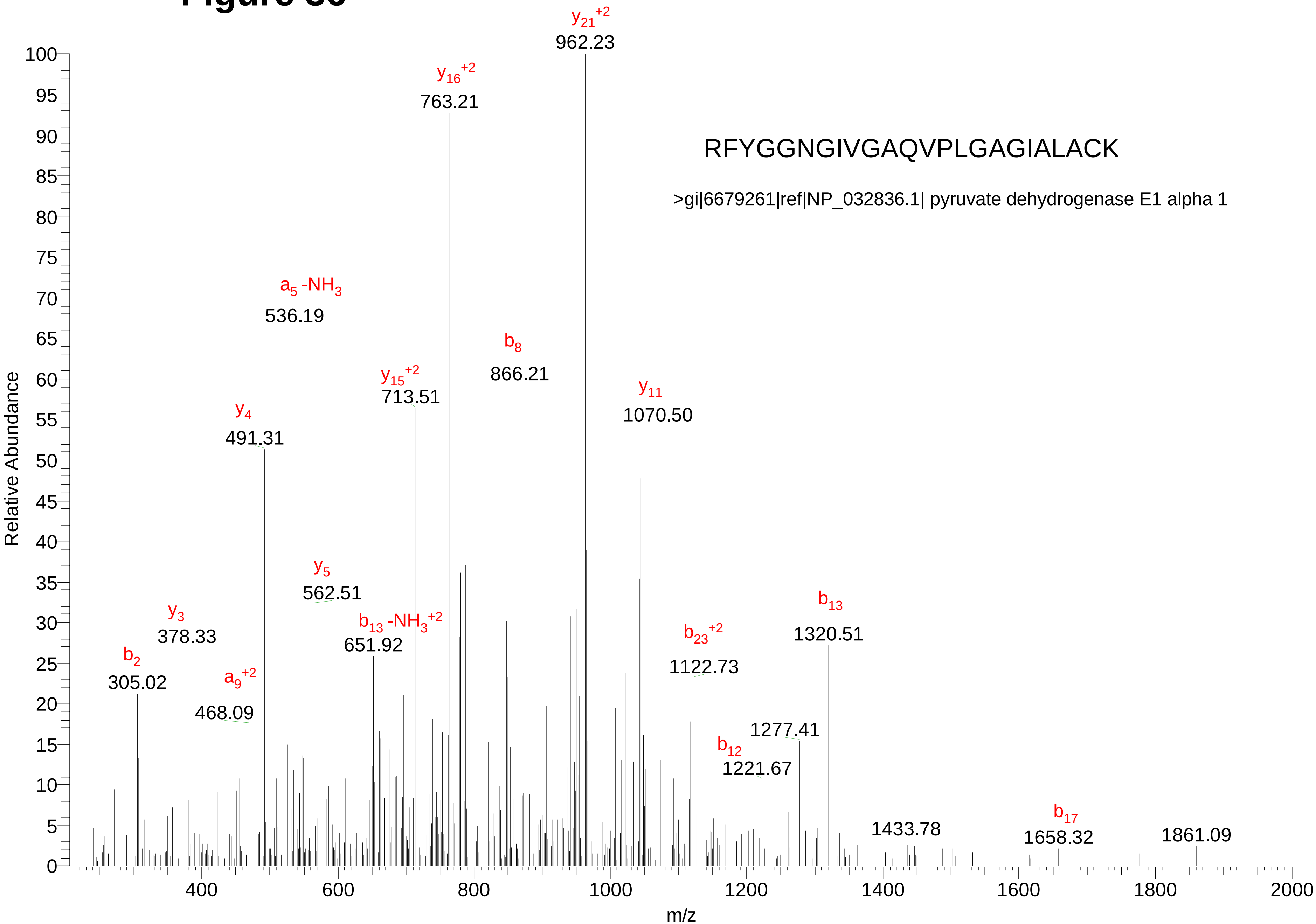
RFYGGNGIVGAQVPLGAGIALACK
>gi|6679261|ref|NP_032836.1| pyruvate dehydrogenase E1 alpha 1
Relative Abundance
m/z
y21+2 962.23
y16+2 763.21
a5 -NH3 536.19
b8 866.21
y15+2 713.51
y11 1070.50
y4 491.31
y5 562.51
b13 -NH3+2 651.92
y3 378.33
b2 305.02
a9+2 468.09
b23+2 1122.73
b13 1320.51
1277.41
b12 1221.67
1433.78
b17 1658.32
1861.09

Figure 37

>gi|20137006|ref|NP_071855.1| myosin, heavy polypeptide 9, non-muscle isoform 1

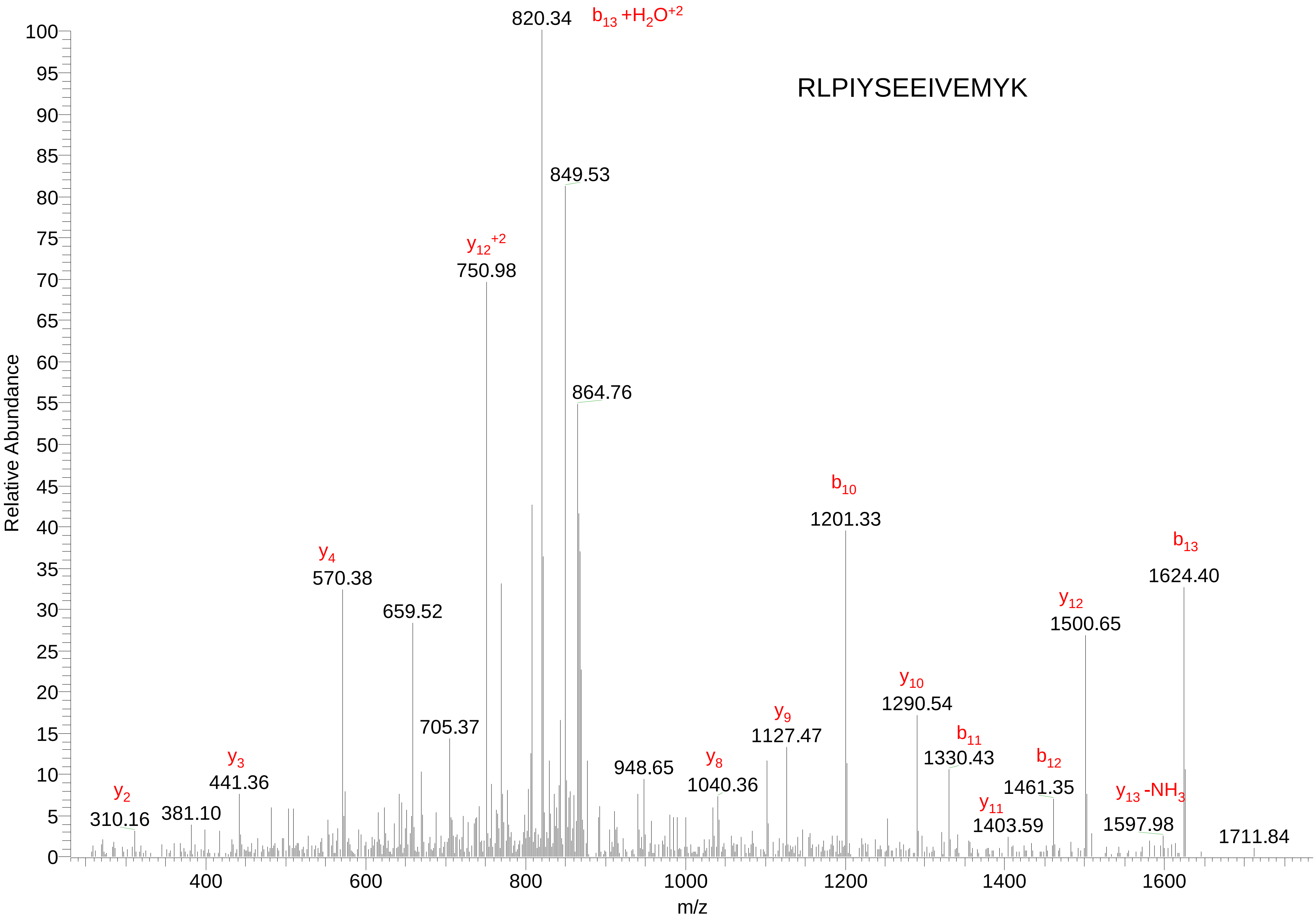

Figure 38

>gi|6677833|ref|NP_033138.1| S100 calcium binding protein A10 (calpactin)

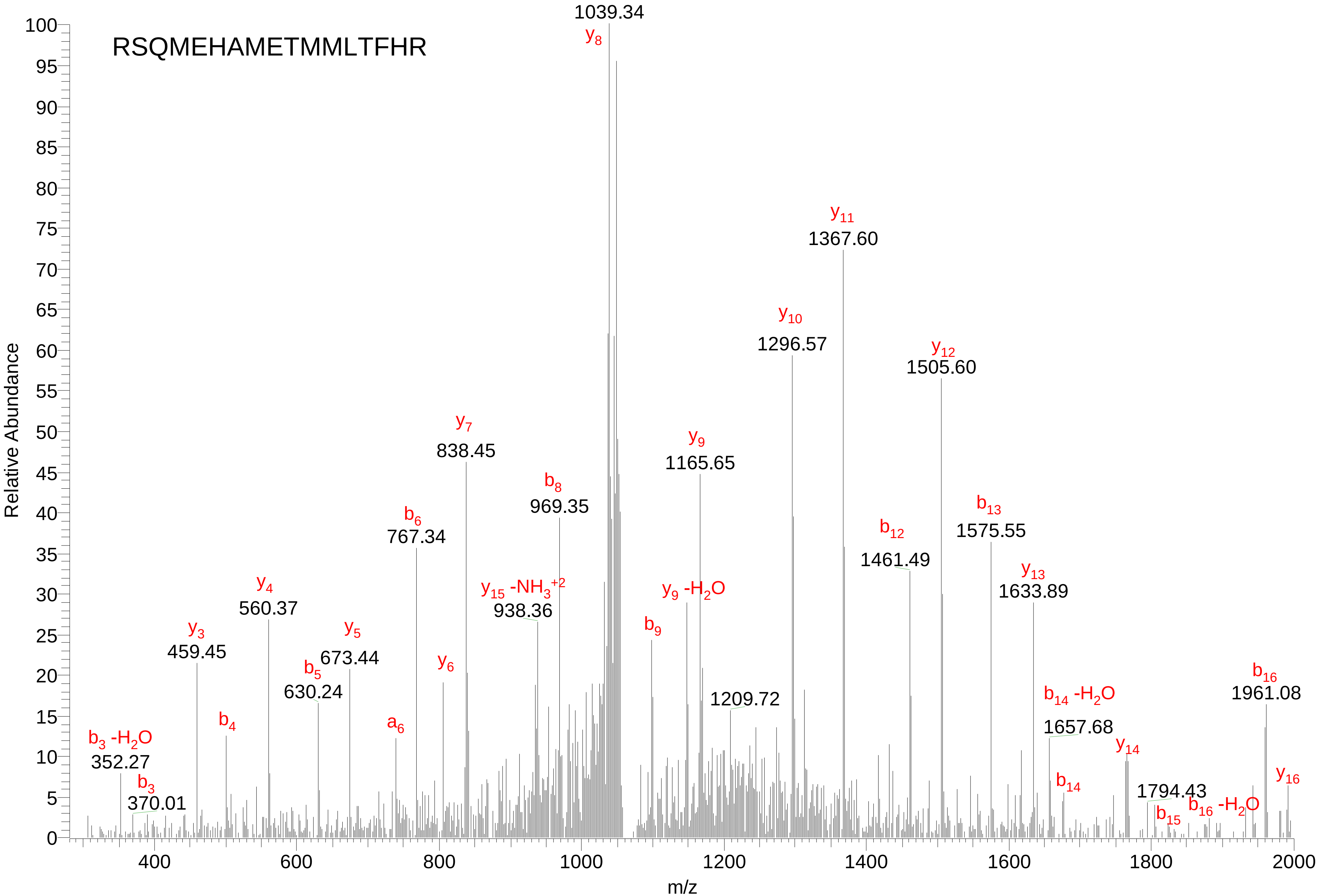

Figure 39

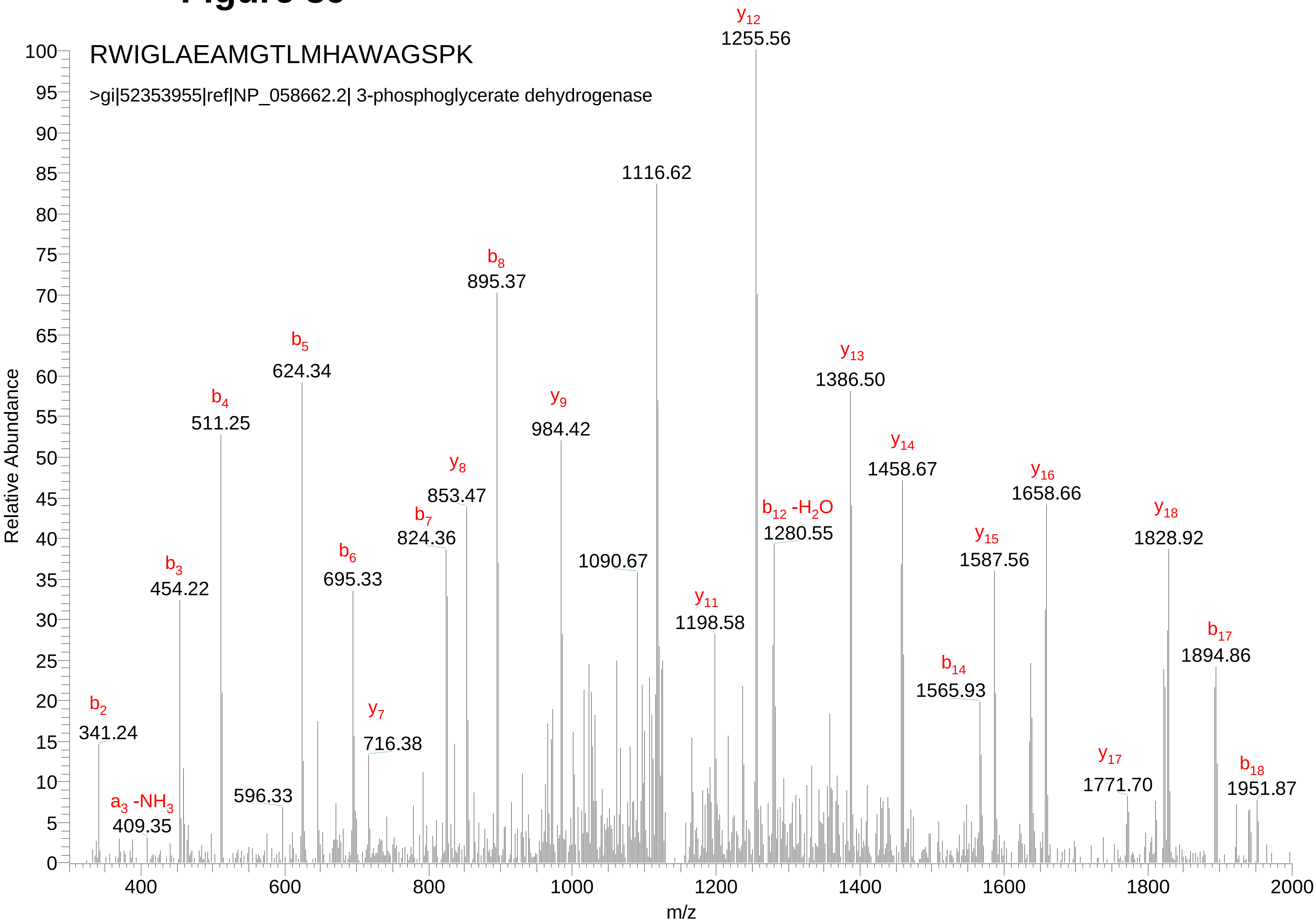

RWIGLAEAMGTLMHAWAGSPK
>gi|52353955|ref|NP_058662.2| 3-phosphoglycerate dehydrogenase
Relative Abundance
m/z
y12 1255.56
1116.62
b8 895.37
b5 624.34
y13 1386.50
b4 511.25
y9 984.42
y14 1458.67
y8 853.47
y16 1658.66
b7 824.36
b12 -H2O 1280.55
y18 1828.92
b6 695.33
1090.67
y15 1587.56
b3 454.22
y11 1198.58
b17 1894.86
b14 1565.93
b2 341.24
y7 716.38
y17 1771.70
b18 1951.87
a3 -NH3 409.35
596.33

Figure 40

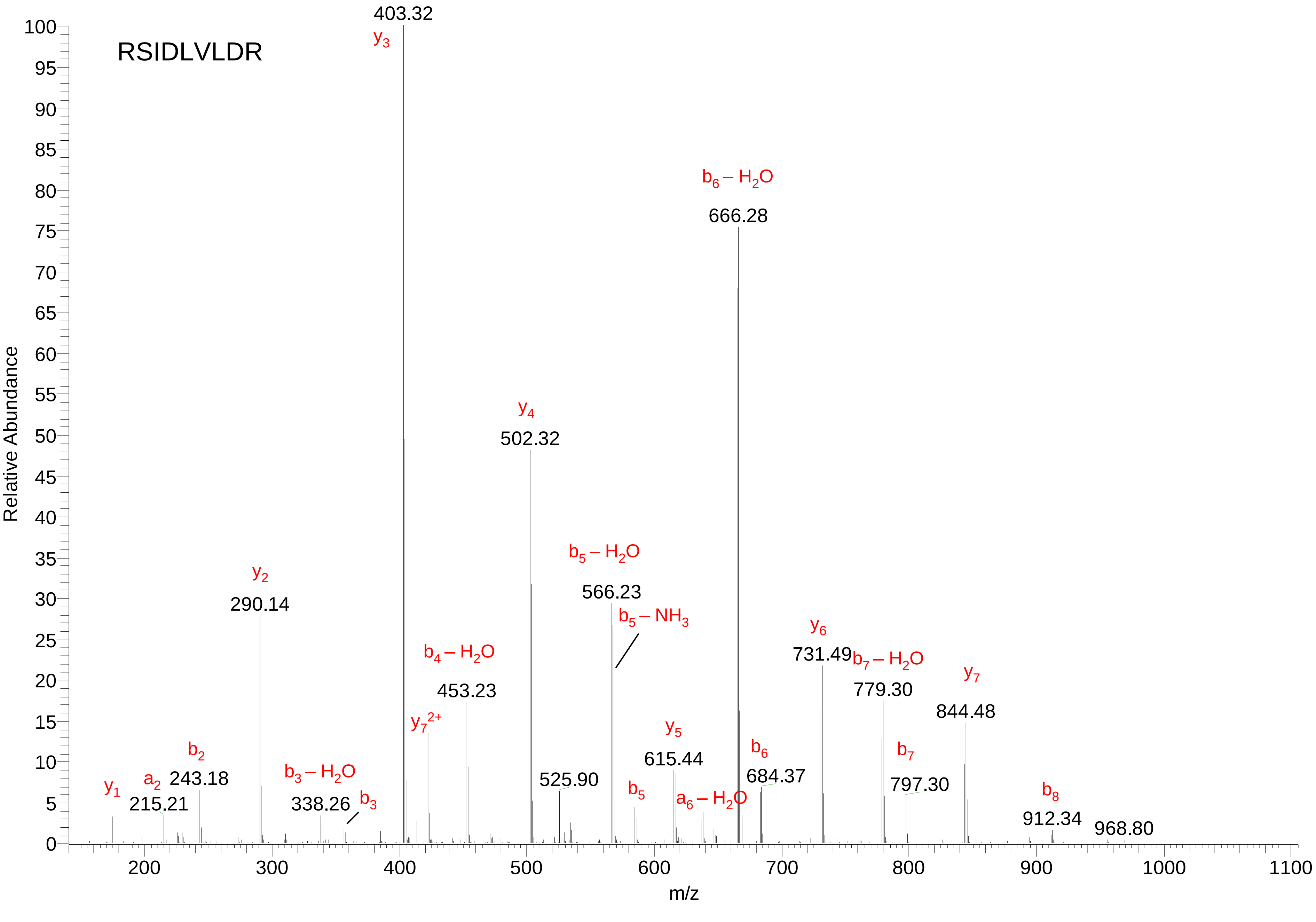

>gi|12585372|sp|Q9JJZ2|TBA8_MOUSE Tubulin alpha-8 chain
RSIDLVLDR
Relative Abundance
m/z
403.32
y3
666.28
b6 – H2O
502.32
y4
566.23
b5 – H2O
b5 – NH3
290.14
y2
731.49
y6
779.30
b7 – H2O
844.48
y7
453.23
b4 – H2O
y7 2+
615.44
y5
684.37
b6
797.30
b7
525.90
b5
a6 – H2O
243.18
b2
a2
215.21
y1
338.26
b3 – H2O
b3
912.34
b8
968.80

Figure 41

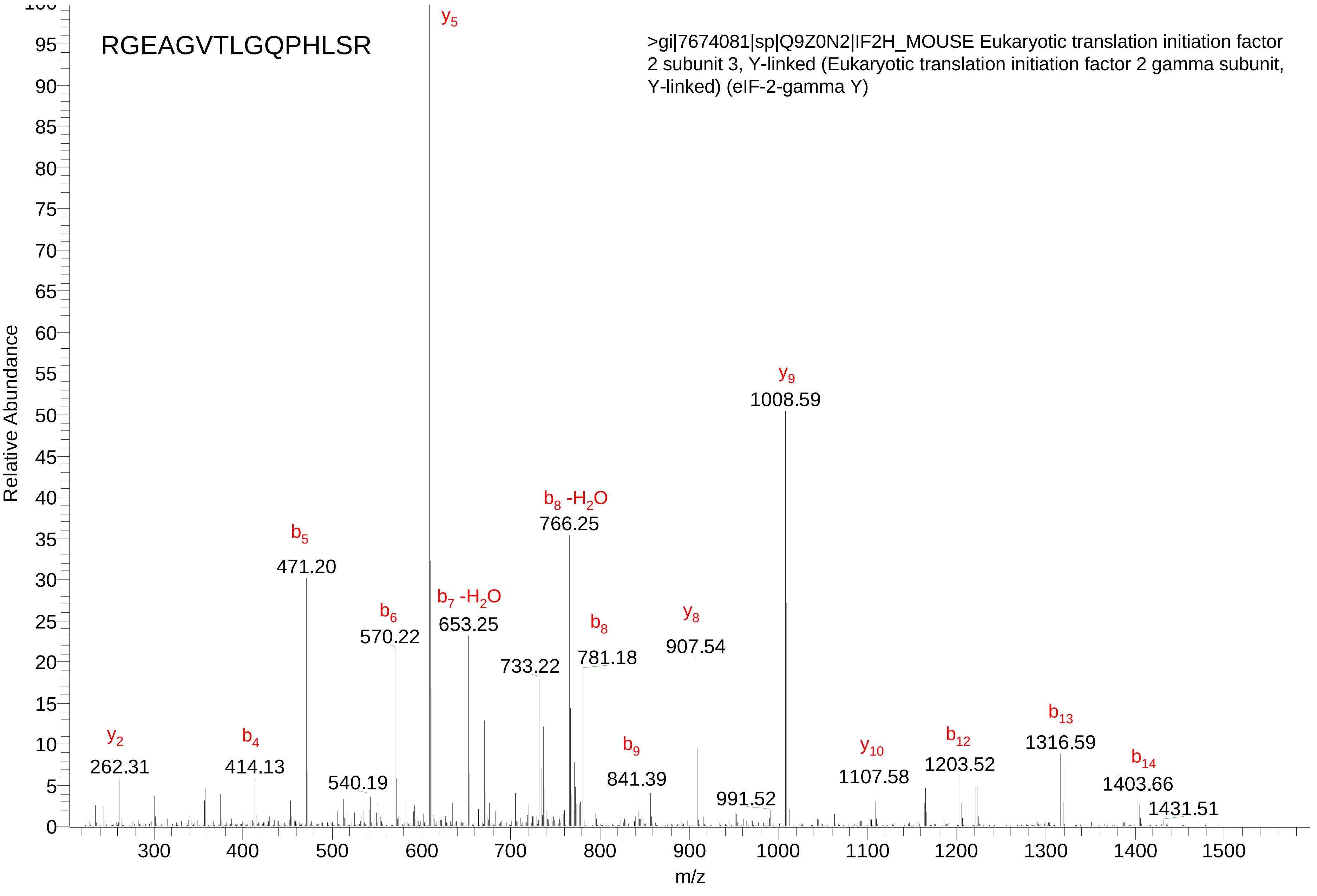

RGEAGVTLGQPHLSR
>gi|7674081|sp|Q9Z0N2|IF2H_MOUSE Eukaryotic translation initiation factor 2 subunit 3, Y-linked (Eukaryotic translation initiation factor 2 gamma subunit, Y-linked) (eIF-2-gamma Y)
Relative Abundance
m/z
y5
y9
1008.59
b8 -H2O
766.25
b5
471.20
b6
570.22
b7 -H2O
653.25
733.22
b8
781.18
y8
907.54
y2
262.31
b4
414.13
540.19
b9
841.39
991.52
y10
1107.58
b12
1203.52
b13
1316.59
b14
1403.66
1431.51

Figure 42

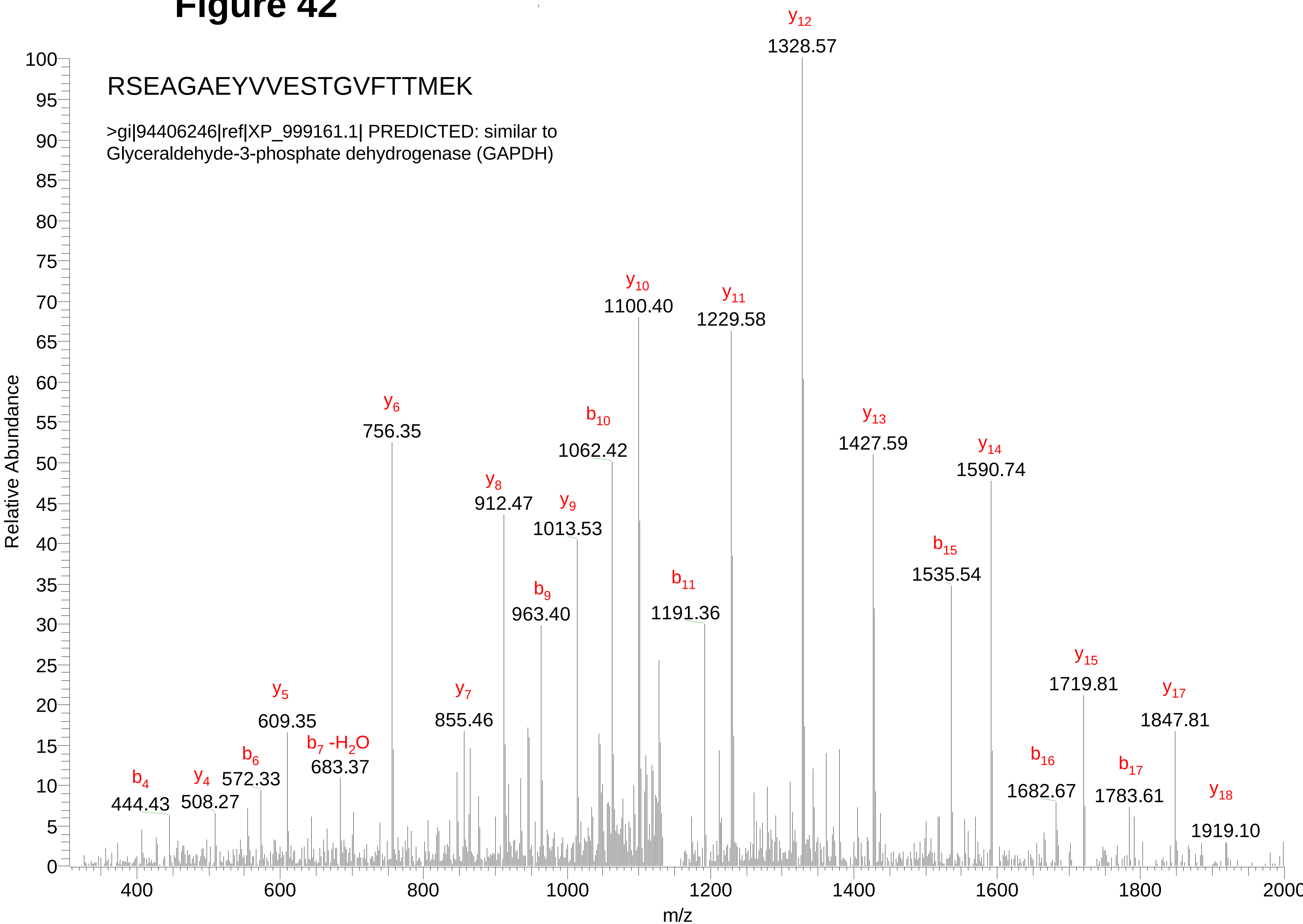
RSEAGAEYVVESTGVFTTMEK
>gi|94406246|ref|XP_999161.1| PREDICTED: similar to
Glyceraldehyde-3-phosphate dehydrogenase (GAPDH)
Relative Abundance
m/z
y12 1328.57
y10 1100.40
y11 1229.58
y6 756.35
b10 1062.42
y13 1427.59
y14 1590.74
y8 912.47
y9 1013.53
b15 1535.54
b9 963.40
b11 1191.36
y15 1719.81
y17 1847.81
y5 609.35
y7 855.46
b7 -H2O 683.37
b6 572.33
b16 1682.67
b17 1783.61
y4 508.27
b4 444.43
y18 1919.10

Figure 43

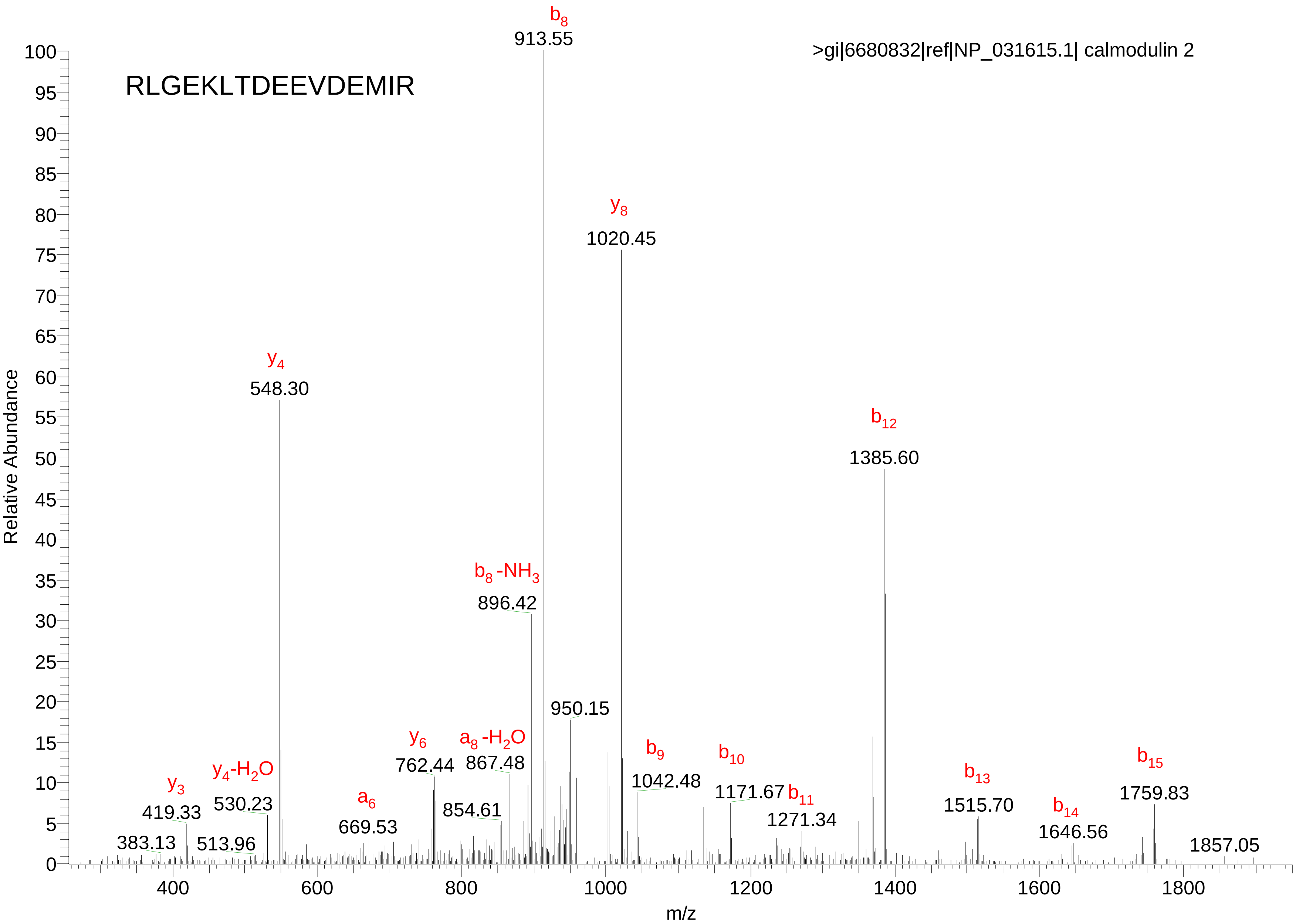

>gi|6680832|ref|NP_031615.1| calmodulin 2
RLGEKLTDEEVDEMIR
Relative Abundance
m/z
b8
913.55
y8
1020.45
y4
548.30
b12
1385.60
b8 -NH3
896.42
950.15
y6
762.44
a8 -H2O
867.48
b9
1042.48
b10
1171.67
b11
1271.34
b13
1515.70
b14
1646.56
b15
1759.83
1857.05
y3
419.33
y4-H2O
530.23
a6
669.53
854.61
383.13
513.96
0
5
10
15
20
25
30
35
40
45
50
55
60
65
70
75
80
85
90
95
100
400
600
800
1000
1200
1400
1600
1800

Figure 44

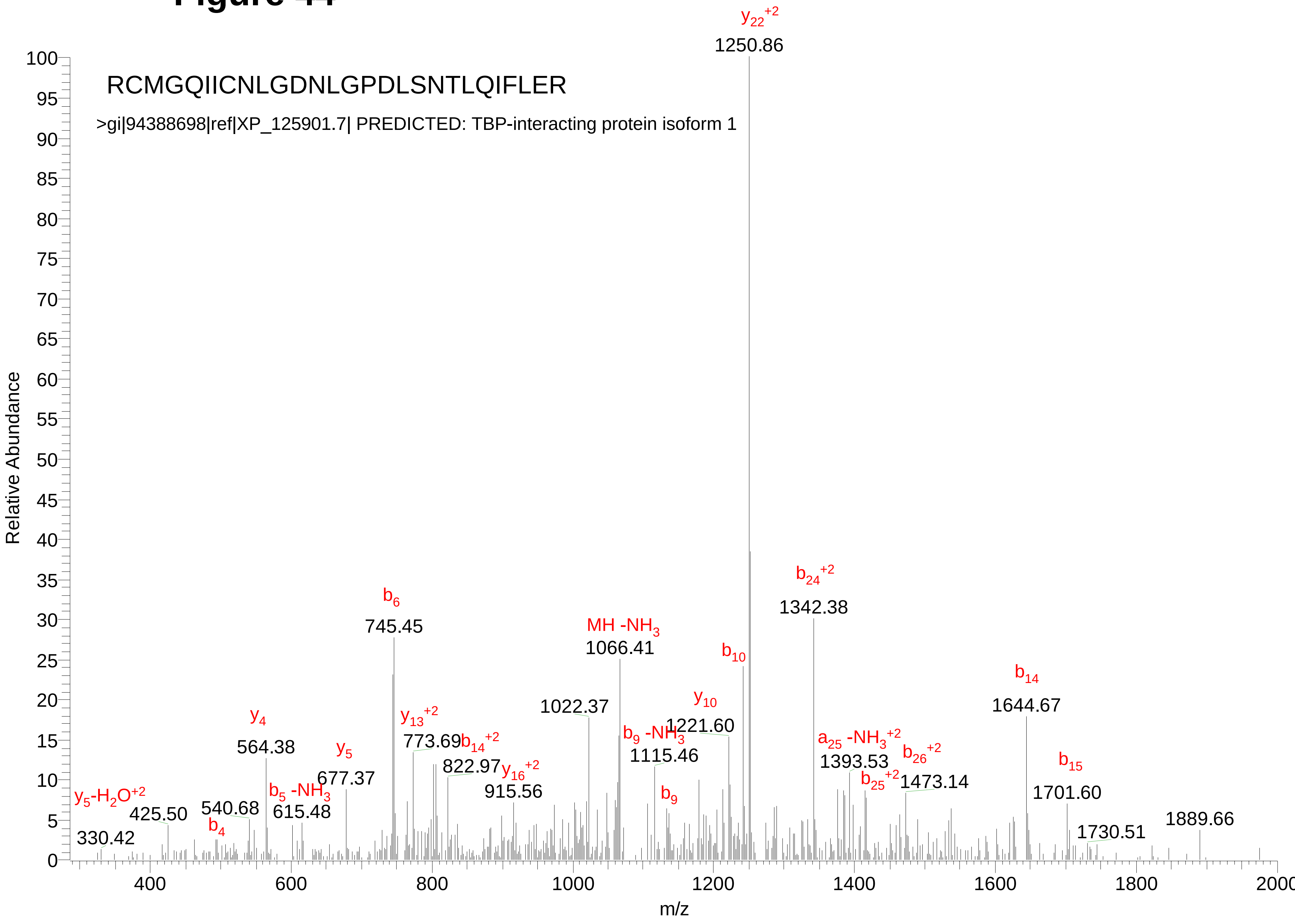
RCMGQIICNLGDNLGPDLSNTLQIFLER
>gi|94388698|ref|XP_125901.7| PREDICTED: TBP-interacting protein isoform 1
Relative Abundance
m/z
y22+2 1250.86
b24+2 1342.38
b6 745.45
MH -NH3 1066.41
b10
b14 1644.67
y10 1221.60
1022.37
b9 -NH3 1115.46
y13+2 773.69
b14+2 822.97
y16+2 915.56
y4 564.38
y5 677.37
b5 -NH3 615.48
540.68
b4
425.50
y5-H2O+2 330.42
a25 -NH3+2 1393.53
b26+2 1473.14
b25+2
b9
b15 1701.60
1730.51
1889.66
0
5
10
15
20
25
30
35
40
45
50
55
60
65
70
75
80
85
90
95
100
400
600
800
1000
1200
1400
1600
1800
2000
