# Supplementary material for: Global Analysis of Posttranslational Protein Arginylation
Source: PLoS Biol. 2007 Sep 25;5(10):e258. doi: 10.1371/journal.pbio.0050258 (PMC1988855; doi:10.1371/journal.pbio.0050258)
Supplement: Supplementary file 2 [file pbio.0050258.sd002.pdf]

Fig. 1-2. MS/MS spectra for the standard peptides used to differentiate the N-terminal R- (Figure 1) and VG- (Figure 2) in the peptides. While precursor masses of the two peptides are similar, the arginylated peptide shows prominent peaks for b ions, while the spectrum for the peptide with N-terminal VG is abundant in y ions only.

Figure 1

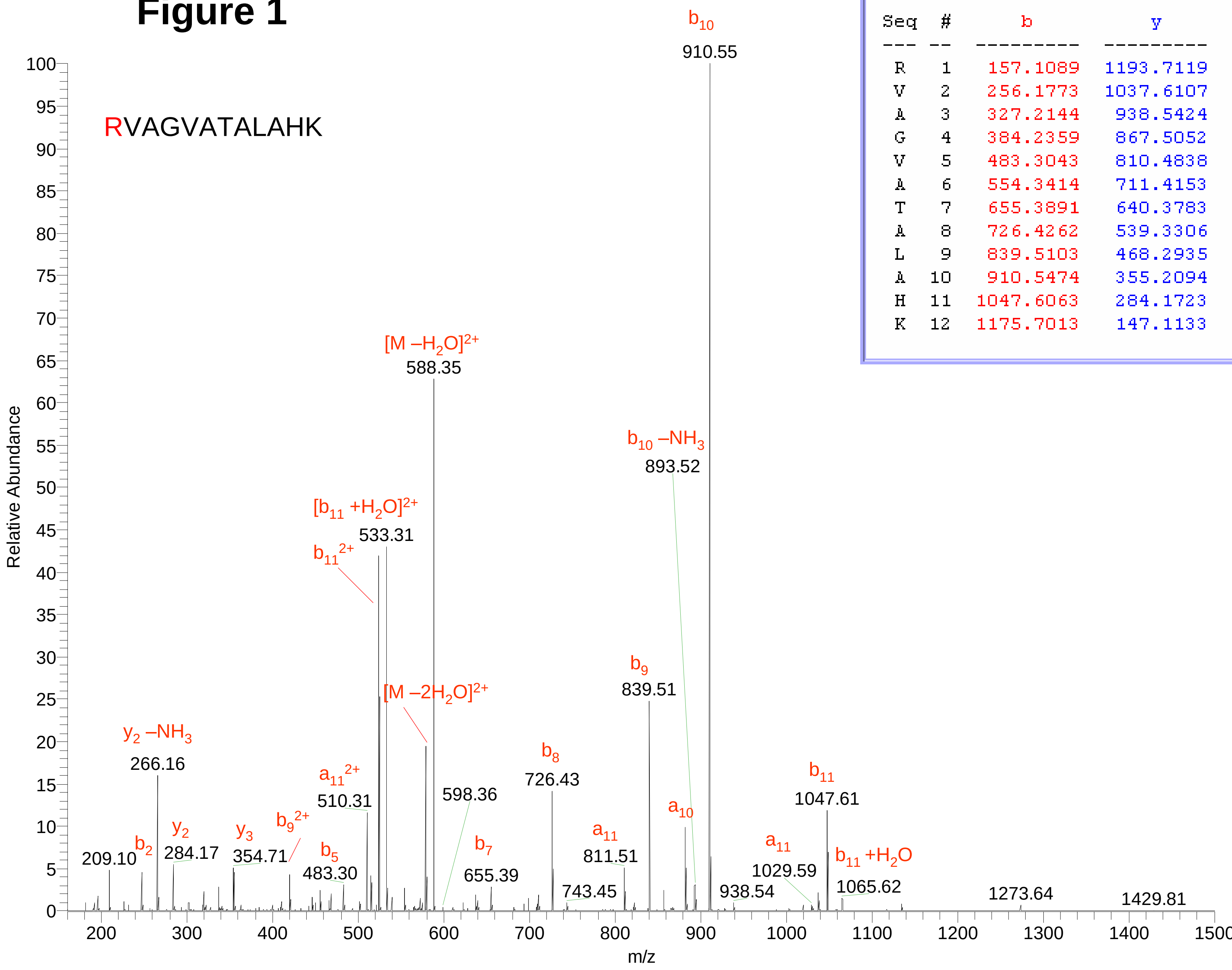

Seq	#	b	y	(+1)
R	1	157.1089	1193.7119	12
V	2	256.1773	1037.6107	11
A	3	327.2144	938.5424	10
G	4	384.2359	867.5052	9
V	5	483.3043	810.4838	8
A	6	554.3414	711.4153	7
T	7	655.3891	640.3783	6
A	8	726.4262	539.3306	5
L	9	839.5103	468.2935	4
A	10	910.5474	355.2094	3
H	11	1047.6063	284.1723	2
K	12	1175.7013	147.1133	1

Figure 2

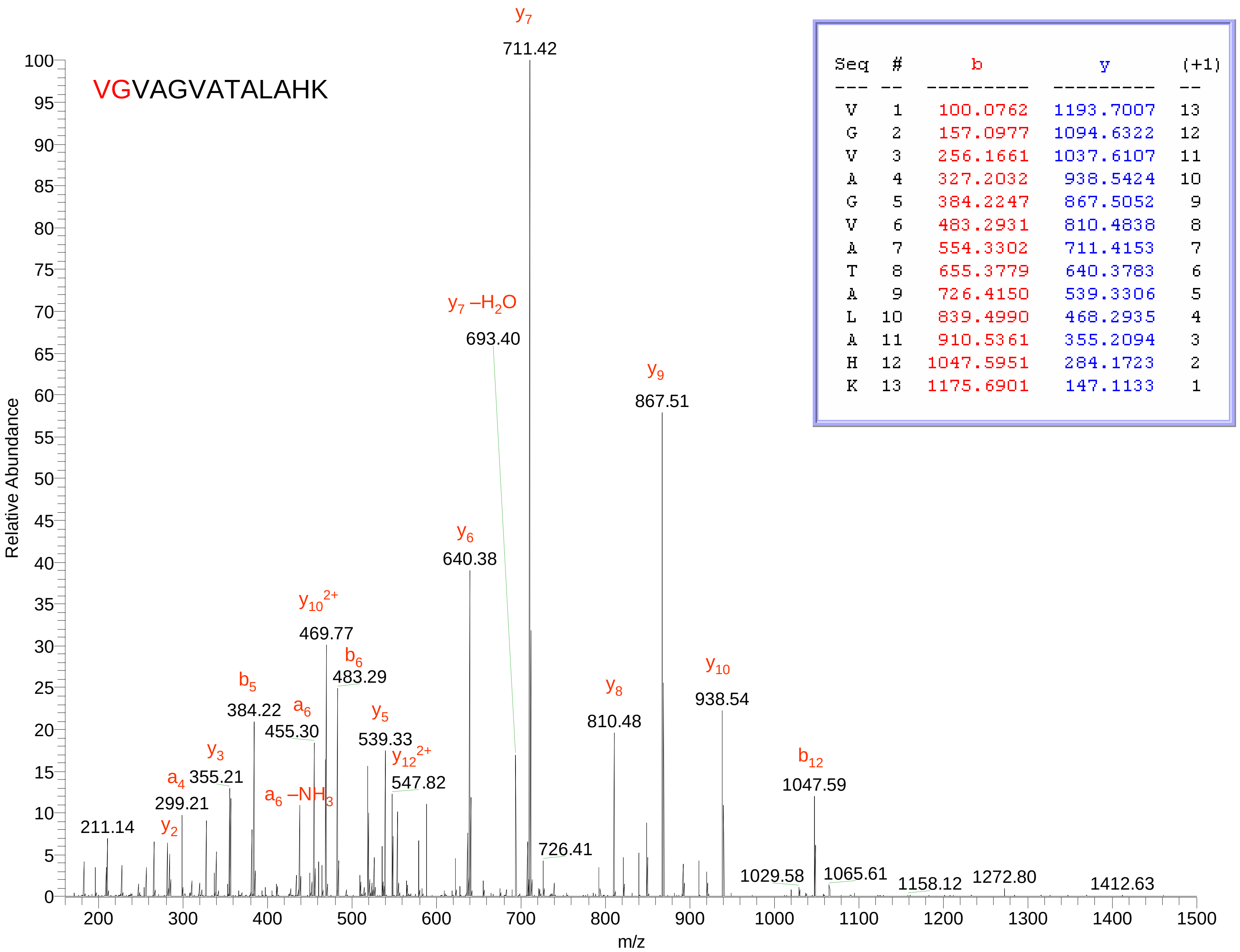

Seq	#	b	y	(+1)
V	1	100.0762	1193.7007	13
G	2	157.0977	1094.6322	12
V	3	256.1661	1037.6107	11
A	4	327.2032	938.5424	10
G	5	384.2247	867.5052	9
V	6	483.2931	810.4838	8
A	7	554.3302	711.4153	7
T	8	655.3779	640.3783	6
A	9	726.4150	539.3306	5
L	10	839.4990	468.2935	4
A	11	910.5361	355.2094	3
H	12	1047.5951	284.1723	2
K	13	1175.6901	147.1133	1
