# Supplementary material for: Global Analysis of Posttranslational Protein Arginylation
Source: PLoS Biol. 2007 Sep 25;5(10):e258. doi: 10.1371/journal.pbio.0050258 (PMC1988855; doi:10.1371/journal.pbio.0050258)
Supplement: Supplementary file 3 [file pbio.0050258.sg001.pdf]

Fig. S1. Frequencies of occurrence of different amino acids in the arginylated position and 20 flanking positions on each side of the arginylated residue calculated with the online WebLogo tool. Letter size corresponds to the frequency of the residue occurrence in each position adjusted to the total frequency of its occurrence in vivo.

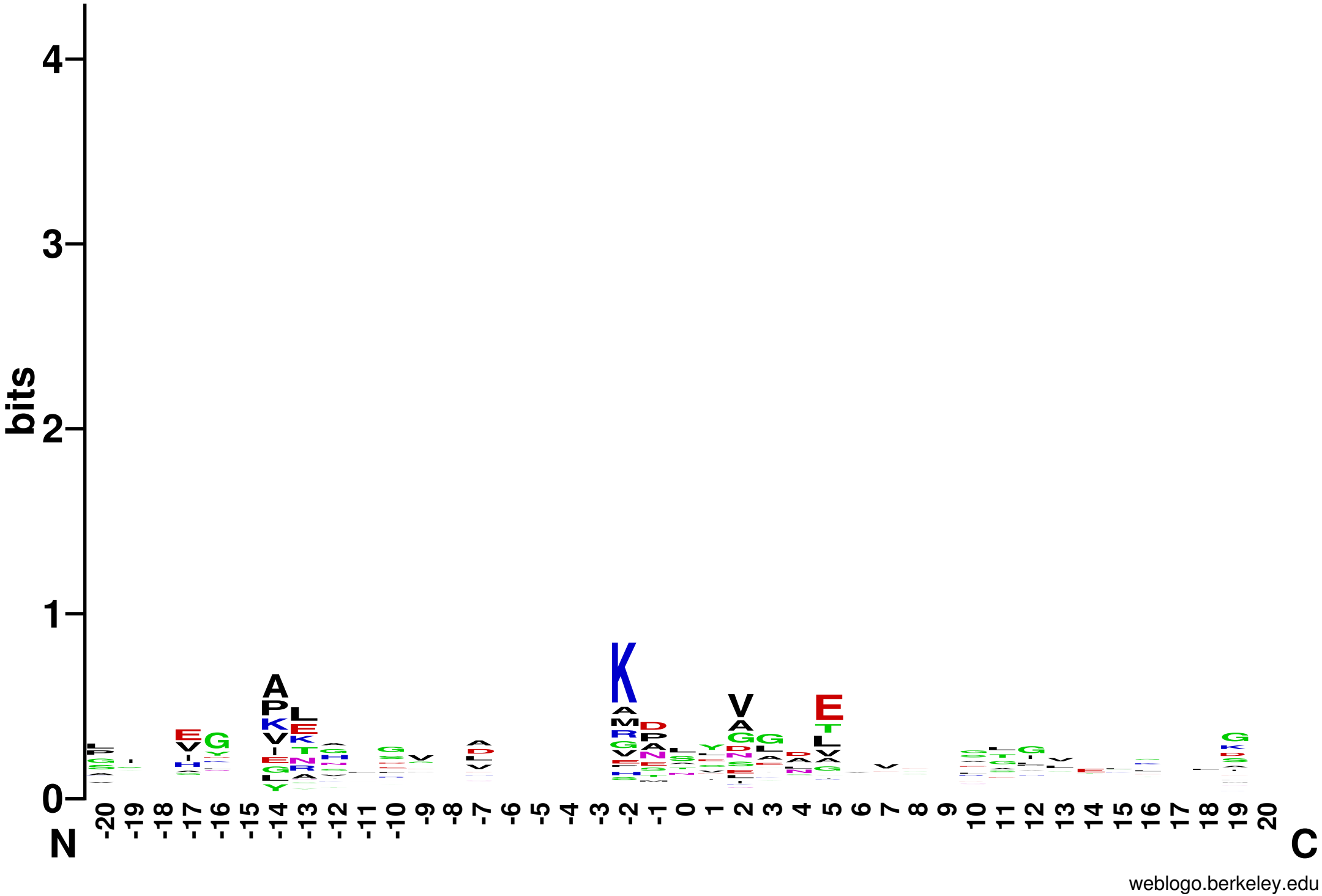
bits
4
3
2
1
0
-20 -19 -18 -17 -16 -15 -14 -13 -12 -11 -10 -9 -8 -7 -6 -5 -4 -3 -2 -1 0 1 2 3 4 5 6 7 8 9 10 11 12 13 14 15 16 17 18 19 20
N
C
weblogo.berkeley.edu
