# Supplementary material for: Global Analysis of Posttranslational Protein Arginylation
Source: PLoS Biol. 2007 Sep 25;5(10):e258. doi: 10.1371/journal.pbio.0050258 (PMC1988855; doi:10.1371/journal.pbio.0050258)
Supplement: Supplementary file 4 [file pbio.0050258.sd003.doc]

**Detailed legend to Figure 6: possible ways of regulation of actin, tubulin, hemoglobin, and GAPDH by arginylation.**

1. Actins.

Actin isoforms appear to be differentially arginylated. Beta actin is arginylated on the N-terminus, which has been shown to regulate the interaction between filaments in vivo. In addition, all three actin isoforms share a common arginylation site (S52/54 for non-muscle actins and alpha actin, respectively), found on the same surface as the N-terminus in the assembled subunit; this site could potentially be involved in neutralizing the mild negative charge of the unarginylated actin N-terminus, which could regulate surface properties and potentially facilitate filament interaction. Similar function could be attributed to the site at F90. Since these two sites are common for both beta and gamma non-muscle actins, there is no way to distinguish whether one or both of these isoforms are modified on these sites, however a mechanism could be envisioned where arginyltrasnferase complex recognizes the N-terminus of one isoform (beta) and then adds arginines on other adjacent surface sites; this possibility constitutes an exciting direction of future studies.

Alpha cardiac actin contains an additional site at L295, which does not appear in other actin isoforms. This site is located on the surface of the actin subunit opposite to the N-terminus. Thus surface is involved in subunit interaction during polymerization and addition of arginine at that site is likely to take modified subunits out of the polymerization pool and could therefore be envisioned to regulate the amount of polymer and actin assembly in cardiac cells. Given the severe cardiovascular phenotypes of Ate1-/- mice, which have underdeveloped hearts, such an effect on alpha cardiac actin is consistent with actin’s physiological role.

2. Tubulin.

Arginine residues in alpha and beta tubulin subunits (2 in beta and 1 in alpha) are located in the middle of the polypeptide chain, however they are found within the same surface of the alpha/beta tubulin dimer in the folded molecule. The arginylated surface is located at the subunit interface in the microtubule; it appears that such arginylation would regulate microtubule polymerization by either preventing or facilitating subunit interaction.

3. Hemoglobin and GAPDH.

Hemoglobin molecule is a tetramer of two alpha and two beta chains. Both alpha and beta chain contain a single arginylation site that is exposed on the surface of the folded subunits, putting four arginines on the outside of the assembled molecule. It is conceivable that such arginylation would regulate the surface properties of hemoglobin.

Similar location of arginylated sites is observed in the assembled GAPDH tetramer, with four arginines exposed on the outmost regions of the assembled molecule. Like hemoglobin, such arginylation is bound to regulate surface properties and molecular interations.
