# Supplementary material for: Global Analysis of Posttranslational Protein Arginylation
Source: PLoS Biol. 2007 Sep 25;5(10):e258. doi: 10.1371/journal.pbio.0050258 (PMC1988855; doi:10.1371/journal.pbio.0050258)
Supplement: Supplementary file 5 [file pbio.0050258.st001.doc]

**Supplemental Table 1. Proteins up-or down regulated in response to Ate1 knockout*^#^**

| **Name** | **Accession** | | **Gel MW**  **(KDa)** | | **PI** | **Aa residues** | **Peptides (amt)** |
| --- | --- | --- | --- | --- | --- | --- | --- |
| **Present only in +/+** | |  | |  |  |  |  |
| - Transitional endoplasmic reticulum ATPase - Nonmuscle heavy chain myosin II-A, Myh9^#^ | S25197  CAC85955 | | 132 | | 5.66 | 806  1946 | 49  4 |
| - heat shock protein 4 | NP_032326 | | 165 | | 5.66 | 841 | 28 |
| - Alpha crystallin B chain - Ribosomal protein L12 | P23927  AAH81469 | | 20 | | 7.83 | 175  165 | 21  2 |
| - heterogeneous nuclear ribonucleoprotein K - alpha isoform of regulatory subunit A, protein phosphatase 2 | AAH06694  NP_058587 | | 69.5 | | 5.63 | 464  589 | 18  2 |
| - pyruvate kinase 3^#^ - chaperonin^#^ subunit 6a (zeta) | NP_035229  NP_033968 | | 57 | | 7.74 | 531  531 | 49  2 |
| - ribosomal protein L3 - isocitrate dehydrogenase 1 (NADP+), soluble - cold shock domain protein A short isoform - phosphogluconate dehydrogenase | NP_038790  NP_034627  NP_035863  AAH14793 | | 48 | | 7.89 | 403  414  361  309 | 18  14  3  2 |
| - histone H1^#^ - Glutathione S-transferase omega 1 | CAA86288  AAH85165 | | 30.6 | | 7.49 | 221  240 | 3  2 |
| - heat shock protein^#^ HSP27 - Ribosomal protein S8 | AAA18335  AAH81465 | | 27.6 | | 6.36 | 208  208 | 5 |
| - deoxyuridine triphosphatase - Histone H4 | NP_076084  CAA72967 | | 22.4 | | 5.9 | 162  103 | 4  2 |
| **Up-regulated in +/+ 3-fold or more** | |  | |  |  |  |  |
| - enolase 1, alpha non-neuron - Y box transcription factor Nsep1 - Rab GDP dissociation inhibitor beta-2 | NP_075608  AAA63390  Q61598 | | 47 | | 7.27 | 434  322  445 | 5  4  2 |
| - alpha-2-macroglobulin receptor-associated protein precursor | JX0281 | | 42 | | 7.74 | 360 | 2 |
| - **beta actin^#^** - novel protein similar to mouse von Ebner salivary gland protein | XP_125034  NA | | 41 | | 7.34 | 352  NA | 4  3 |
| - Calponin 3, acidic | AAH85268 | | 39.8 | | 7.35 | 330 | 3 |
| - Twinfilin (actin minomer binding) - Heterogeneous nuclear ribonucleoprotein A/B - Ubiquitin fusion degradation 1 like - Poly(rC)-binding protein 3 (Alpha-CP3) | NP_032997  Q99020  AAH06630  P57722 | | 43 | | 6.87 | 350  285  307  339 | 3  3  2  2 |
| - nucleophosmin 1 - annexin I | NP_032748  LUMS1 | | 42 | | 6.89 | 292  346 | 5  2 |
| - Acidic ribosomal phosphoprotein P0 - PDZ and LIM domain protein 1 (LIM domain protein CLP-36) (C-terminal LIM domain protein 1) (Elfin)^#^. - annexin I - Mitochondrial ribosomal protein L39 | AAH11106  O70400  LUMS1  NP_059100 | | 40 | | 6.72 | 317  326  346  297 | 9  5  4  2 |
| - Protein phosphatase 1 | AAA37526 | | 39.99 | | 5.91 | 337 | 2 |
| - Phosphoglycerate mutase 1 | Q9DBJ1 | | 29 | | 7.11 | 254 | 4 |
| **Present only in -/-** | |  | |  |  |  |  |
| - lamin A/C - pyruvate kinase 3^#^ - chaperonin subunit 6a (zeta) | P48678 NP_035229  NP_033968 | | 57.7 | | 7.54 | 665  531  531 | 17  10  7 |
| - protein synthesis initiation factor 4A - **Actin^#^, alpha/beta/gamma** - eukaryotic translation initiation factor 3, subunit 4 - 26S protease regulatory subunit 7 | AAA50407  AAH21796  NP_058572  P46471 | | 45 | | 6.19 | 406  375  320  433 | 11  10  5  3 |
| - ribosomal protein L3 - phosphogluconate dehydrogenase - **Actin^#^, alpha/beta/gamma** - proliferation-associated 2G4 | NP_038790  NP_080077  AAH21796  NP_035249 | | 45.6 | | 7.48 | 403  483  375  394 | 4  2  2  2 |
| - peptidylprolyl isomerase D - mitotic checkpoint protein BUB3 - Aspartate aminotransferase, mitochondrial precursor | NP_080628  AAD38038  P05202 | | 39.9 | | 7.45 | 370  326  430 | 5  4  2 |
| - Med8 protein - tyrosine 3-monooxygenase\/tryptophan 5-monooxygenase activation protein, epsilon polypeptide (Protein kinase C inhibitor protein-1) - guanine nucleotide binding protein alpha stimulating isoform c | AAH21870  CAI26030  NP_068840 | | Nd | | Nd | 268  255  253 | 1  1  1 |
| - Annexin A2 | AAH03327 | | 33.9 | | 7.7 | 339 | 13 |
| - Annexin A2 - similar to glyceraldehyde-3-phosphate dehydrogenase^#^ - annexin I - Fibrillarin | AAH03327  XP_486720  LUMS1  AAH03813 | | 33.8 | | 7.57 | 339  333  346  327 | 19  12  4  2 |
| - Med8 protein - guanine nucleotide binding protein alpha stimulating isoform c | AAH21870  NP_068840 | | Nd | | Nd | 268  253 | 1  1 |
| - Ubiquitin carboxyl-terminal esterase L3 - tyrosine 3-monooxygenase\/tryptophan 5-monooxygenase activation protein, epsilon polypeptide (Protein kinase C inhibitor protein-1) | AAH48481  CAI26030 | | 28 | | 5.38 | 230  255 | 11  8 |
| - Rho, GDP dissociation inhibitor (GDI) beta - Phosphatidylethanolamine-binding protein (PEBP) (HCNPpp) (Contains: Hippocampal cholinergic neurostimulating peptide (HCNP)) - unnamed protein product - Peroxiredoxin 2 (Thioredoxin peroxidase 1) | NP_031512  P70296  BAB26609  Q61171 | | 26 | | 5.4 | 200  187  182  198 | 5  4  4  2 |
| - ATP synthase, H+ transporting, mitochondrial F0 complex, subunit d - Inosine triphosphatase - RNA-binding protein 8A (Ribonucleoprotein RBM8A) - tubulin alpha chain^#^ - Peroxiredoxin 2 (Thioredoxin peroxidase 1) | AAH81431  AAH26508  Q9CWZ3  AAH83344  Q61171 | | 22.7 | | 5.74 | 161  198  174  451  198 | 6  4  3  2  2 |
| **Up-regulated in -/- 3-fold or more** | |  | |  |  |  |  |
| - vitamin D receptor associated factor 1 | JC5402 | | 53.6 | | 4.76 | 319 | 12 |
| - polymerase (RNA) II (DNA directed) polypeptide C - 2010012F05Rik protein - **Actin^#^, alpha/beta/gamma** - nascent polypeptide-associated complex alpha polypeptide - Proliferating cell nuclear antigen - Ubiquitin thiolesterase protein OTUB1 (Otubain 1) | NP_033116  AAH11429  AAH21796  NP_038636  AAH05778  Q7TQI3 | | 38.7 | | 5.34 | 275  224  375  215  261  271 | 6  5  4  3  3  2 |
| - Glutathione S-transferase, mu 1 - Dodecenoyl-Coenzyme A delta isomerase (3,2 trans-enoyl-Coenyme A isomerase) - hydroxyacyl-Coenzyme A dehydrogenase type II - Proteasome^#^ subunit alpha type 7 (Proteasome subunit RC6-1). | AAH46758  AAH22712  NP_058043  Q9Z2U0 | | 28.5 | | 8.86 | 218  289  261  248 | 23  5  3  3 |
| - complement component 1, q subcomponent binding protein - similar to ubiquitin A-52 residue ribosomal protein fusion product1 | CAI26064  XP_356994 | | 24.7 | | 4.66 | 279  174 | 1  1 |
| - Ribosomal protein L12 - Ribosomal protein L24 - S-phase kinase-associated protein 1A (Cyclin A/CDK2-associated protein p19) (p19A) (p19skp1). | AAH81469  AAH53377  Q9WTX5 | | 20 | | 7.28 | 165  157  163 | 3  2  2 |

* note: proteins with mutually exclusive appearance in the two gels (i.e., present only in +/+ and at the same time present only in -/-) are likely exhibiting arginylation-dependent gel shifts or are highly abundant in both gels and are identified as contaminants in non-specific spots.

**^#^**proteins from the Supplemental Table1 that also appear in Table 1 in the main text
