# Supplementary material for: Global Analysis of Posttranslational Protein Arginylation
Source: PLoS Biol. 2007 Sep 25;5(10):e258. doi: 10.1371/journal.pbio.0050258 (PMC1988855; doi:10.1371/journal.pbio.0050258)
Supplement: Supplementary file 6 [file pbio.0050258.st002.doc]

**Supplemental Table 2. Proteins up- regulated in response to proteasome inhibition**

(only proteins with more than 3 peptides identified are reported)

3-ketoacyl-CoA thiolase B	NP_666342

40S ribosomal protein S26.	P62855

60S acidic ribosomal protein P0 (L10E)	P14869

5',3'-nucleotidase, cytosolic	NP_056622

acetyl-Coenzyme A acyltransferase 1	NP_570934

acidic ribosomal phosphoprotein P0	NP_031501

actin^#^	AAA37164

ADP-ribosylation factor 1^#^	NP_031502

ADP-ribosylation factor 3	NP_031504

aldehyde dehydrogenase 2, mitochondrial	NP_033786

aminolevulinate, delta-, dehydratase	NP_032551

aminopeptidase	AAD43215

annexin A2	NP_031611

ATP synthase, H+ transporting mitochondrial

F1 complex, beta subunit	NP_058054

ATP-binding cassette, subfamily E, member 1	NP_056566

BC031593 protein	AAH19155

calpain, small subunit 1	NP_033925

calponin 3, acidic	NP_082320

calreticulin	NP_031617

capping protein (actin filament) muscle Z-line,

alpha 2	NP_031630

carbonyl reductase 1	NP_031646

casein kinase II, alpha 2, polypeptide	NP_034104

caseinolytic protease, ATP-dependent,

proteolytic subunit homolog	NP_059089

CNDP dipeptidase 2

(metallopeptidase M20 family)	NP_075638

coactosin-like 1	NP_082347

coding region determinant binding protein	AAC72743.1

crystallin, alpha B	NP_034094

cytochrome c oxidase, subunit Va	NP_031773

DEAD (Asp-Glu-Ala-Asp) box polypeptide 5	NP_031866

dihydrolipoamide S-succinyltransferase

(E2 component of 2-oxoglutarate complex)	NP_084501

dihydropyrimidinase-like 2	NP_034085

double cortin and calcium/calmodulin-

dependent protein kinaselike 1	NP_064362

doublecortin	NP_034155

EH-domain containing 2	NP_694708

ELAV (embryonic lethal, abnormal vision,

Drosophila)-like 1 (Hu antigen R)	NP_034615

emerin	NP_031953

endoplasmic reticulum transmembrane

protein precursor.	AAA37573

enolase 1, alpha non-neuron	NP_075608

eukaryotic translation elongation factor 1 alpha 1	NP_034236

eukaryotic translation elongation factor 1 alpha 2	NP_031932

eukaryotic translation initiation factor 3, subunit 7

(zeta)	NP_061219

eukaryotic translation termination factor 1	NP_659115

ferritin light chain 1	NP_034370

ferritin light chain 2	NP_032075

fibrillarin	NP_032017

fibronectin 1	NP_034363

gelsolin	NP_666232

Glial fibrillary acidic protein, astrocyte (GFAP).	P03995

Glucosidase 2 beta subunit precursor

(Glucosidase II beta subunit) (Protein kinase

C substrate, 60.1 kDa protein, heavy chain)

(PKCSH) (80K-H protein).	O08795

glutamate oxaloacetate transaminase 2,

mitochondrial	NP_034455

glyceraldehyde-3-phosphate dehydrogenase^#^	NP_032110

growth suppressor 1L	AAF04806.1

H2A histone family, member X	NP_034566

H2A histone family, member Z	NP_058030

heat shock 70kD protein 5

(glucose-regulated protein)	NP_071705

heat shock protein 1	NP_038588

heat shock protein 1, alpha	NP_034610

heat shock protein 1, beta	NP_032328

heat shock protein 105	NP_038587

heat shock protein 4	NP_032326

heat shock 70kD protein 5	AAH50927.1|

heat shock protein 8^#^	NP_112442

hematopoietic lineage switch 2	AAC17945

heterogeneous nuclear ribonucleoprotein A1 isoform b	NP_001034218

heterogeneous nuclear ribonucleoprotein A2/B1 isoform 2	NP_872591

heterogeneous nuclear ribonucleoprotein C	NP_058580

heterogeneous nuclear ribonucleoprotein H1	NP_067485

heterogeneous nuclear ribonucleoprotein H2	NP_063921

heterogeneous nuclear ribonucleoprotein K	NP_079555

heterogeneous nuclear ribonucleoprotein L	NP_796275

hexosaminidase A	NP_034551

H2A histone family, member X	NP_034566

H2B histone family, member S,	Q8C622

His2b protein.	Q64477

Hist1h2bp protein (OTTMUSP00000000463).	Q6P8V8

histone 1, H4c	NP_835515

Histone 2b protein	Q64475

histone 3, H2bb	NP_996765

Histone H2B F	P10853

Histone H4.	NP_835515

Histone protein Hist1h2bk (OTTMUSP00000000486).	Q8CGP1

HLA-B-associated transcript 1A	NP_062667

hypothetical protein LOC213673	NP_694757

hypothetical protein LOC66508	NP_079881

hypothetical protein LOC68303	NP_080943

hypothetical protein LOC77055	NP_001028349

hypothetical protein LOC98238	NP_598568

immunoglobulin (CD79A) binding protein 1b	NP_056592

insulin-like growth factor 2 mRNA binding protein 2	NP_898850

insulin-like growth factor 2, binding protein 1	NP_034081

insulin-like growth factor 2, binding protein 3	NP_076159

junction plakoglobin	NP_034723

lamin A isoform A	NP_001002011

lectin, galactose binding, soluble 1	NP_032521

leprecan 1 isoform 1	NP_062756

leprecan 1 isoform 2	NP_062757

leukotriene B4 12-hydroxydehydrogenase	NP_080244

mannose-6-phosphate receptor binding protein 1	NP_080112

MO25 protein	NP_598542

myosin light chain, regulatory B-like	NP_080340

myosin regulatory light chain-like	NP_075891

myosin, heavy polypeptide 7, cardiac muscle, beta	NP_542766

myosin, heavy polypeptide 9, non-muscle isoform 1^#^	NP_071855

nexilin	NP_955759

NS1-associated protein 1 isoform 2	NP_062770

nucleolar protein 5A	NP_077155

nucleophosmin 1	NP_032748

nucleoside-diphosphate kinase 1^#^	NP_032730

peptide-O-fucosyltransferase 2	Q8VHI3

peptidylprolyl isomerase A	NP_032933

peptidylprolyl isomerase B	NP_035279

peroxiredoxin 4	NP_058044

peroxiredoxin 5 precursor	NP_036151

peroxiredoxin 6	NP_031479

phosphatidylinositol-binding clathrin assembly protein	NP_666306

phosphoserine aminotransferase 1	NP_803155

PREDICTED: heterogeneous nuclear ribonucleoprotein A0

isoform 2	XP_896535

PREDICTED: similar to Spectrin alpha chain, brain (Spectrin,

non-erythroid alpha chain) (Alpha-II spectrin) (Fodrin alpha

chain) isoform 6^#^	XP_992016

Programmed cell death protein 8, mitochondrial precursor

(Apoptosis-inducing factor)	Q9Z0X1

protease, serine, 1	NP_444473

proteasome^#^ 26S non-ATPase subunit 5	NP_542121

protein disulfide isomerase associated 3	NP_031978

protein disulfide isomerase-associated 6	NP_082235

protein kinase C and casein kinase substrate in neurons 3	NP_083009

protein O-fucosyltransferase 2	NP_084538

protein phosphatase 2A, regulatory subunit B, delta isoform	NP_080667

pyruvate dehydrogenase^#^ complex, component X	NP_780303

ras-GTPase-activating protein SH3-domain binding protein	NP_038744

RAS-related protein-1a	NP_663516

Rho GDP dissociation inhibitor (GDI) alpha	NP_598557

ribosomal protein L22	NP_033105

ribosomal protein L23a	NP_997406

ribosomal protein L24	NP_077180

ribosomal protein L30	NP_033109

ribosomal protein L31	NP_444487

ribosomal protein S14	NP_065625

ribosomal protein S16	NP_038675

ribosomal protein S18	NP_035426

ribosomal protein S19	NP_075622

ribosomal protein S20	NP_080423

ribosomal protein S23	NP_077137

ribosomal protein S25	NP_077228

ribosomal protein S8	NP_033124

RNA binding motif protein 3	NP_058089

selenoprotein	NP_444332

septin^#^ 8	NP_149156

serine/threonine kinase receptor associated protein	NP_035629

SET translocation	NP_076360

SH3-binding domain glutamic acid-rich protein like	NP_064373

SH3-domain GRB2-like endophilin B2	NP_647463

sideroflexin 3	NP_444427

signal recognition particle 14	NP_033299

similar to glyceraldehyde-3-phosphate dehydrogenase^#^	NP_032110

Similar to H2B histone family, member J.	Q8VEE3

SKI interacting protein	NP_079783

small nuclear ribonucleoprotein D2	NP_081219

stomatin-like protein 2	NP_075720

stress-induced phosphoprotein 1	NP_058017

superoxide dismutase 1, soluble	NP_035564

suppression of tumorigenicity 13	NP_598487

thioredoxin 1	NP_035790

transaminase A	P05202

transketolase	NP_033414

transmembrane emp24 domain-containing protein 10	NP_081051

triosephosphate isomerase 1	NP_033441

tropomyosin 1, alpha	NP_077745

tropomyosin 2, beta	NP_033442

tubulin, alpha 1	NP_035783

tubulin, alpha 2	NP_035784

tubulin, alpha 6	NP_033474

tubulin, beta 4	NP_033477

tubulin, beta 5	NP_035785

tubulin, beta 2c	AAH05547

ubiquitin B	NP_035794

ubiquitin-activating enzyme E1, Chr X	NP_033483

v-crk sarcoma virus CT10 oncogene homolog	NP_598417

v-crk sarcoma virus CT10 oncogene homolog (avian)-like	NP_031790

vimentin^#^	NP_035831

voltage-dependent anion channel 1	NP_035824

WD repeat domain 12	NP_067287

**^#^**proteins from the Supplemental Table 2 that also appear in Table 1 in the main text
