# Supplementary material for: Global Analysis of Posttranslational Protein Arginylation
Source: PLoS Biol. 2007 Sep 25;5(10):e258. doi: 10.1371/journal.pbio.0050258 (PMC1988855; doi:10.1371/journal.pbio.0050258)
Supplement: Supplementary file 7 [file pbio.0050258.st003.doc]

**Supplemental Table 3. Proteins identified in the spots that posttranslationally incorporate arginine in cultured fibroblasts**

**Arginylation increased 3-fold or above**

| **Name** | **Accession** | **Gel MW**  **(KDa)** | **PI** | **Aa residues** | **Peptides (amt)** |
| --- | --- | --- | --- | --- | --- |
| - Vimentin^#^ - Protein disulfide isomerase precursor (PDI) (Prolyl 4-hydroxylase beta) (PDI/PH) | CAA39807  P09103 | 65.434 | 5.28 | 466  509 | 11  8 |
| - Vimentin^#^ - Peripherin 1 (shares the vim. peptides) | CAA39807  CAA42499 | 50.454 | 5.33 | 466  475 | 23  3 |
| - Vimentin^#^ - Desmin (shares the vim. Peptides) | CAA39807  NP_034173 | 46.940 | 5.31 | 466  469 | 22  3 |
| - Tropomyosin 1 alpha | NP_077745 | 35.572 | 5.25 | 284 | 25 |
| - Heat-shock protein beta-1 (HspB1) - Sepiapterin reductase (SPR) - caseinolytic protease, ATP-dependent, proteolytic subunit homolog - peroxiredoxin 6 | P14602  Q64105  NP_059089  NP_031479 | 28.172 | 5.86 | 209  261  272  224 | 6  5  5  3 |
| - Heat-shock protein beta-1 (HspB1) - peroxiredoxin 4 - isopentenyl-diphosphate delta isomerase | P14602  NP_058044  NP_663335 | 27.570 | 6.19 | 209  274  227 | 12+10  6  5 |
| - similar to High mobility group protein 1 (HMG-1) (Amphoterin) (Heparin-binding protein p30) - ribosomal protein L13 - YTH domain family 3 | CAA56631  NP_058018  NP_766265 | 26551 | 6.30 | 215  211  596 | 7x6  2  2 |
| - alpha B-crystallin | NP_034094 | 19.997 | 7.89 | 175 | 4 |
| - Ribosomal protein S2 - proteasome activator subunit 3 - ribosomal protein S3 - ribosomal protein S6 - Ribosomal protein S3a | AAH87956  NP_035322  NP_036182  NP_033122  AAH84675 | 30.961 | 6.18 | 293  254  243  249  264 | 8  4  4  3  2 |
| - Tropomyosin 2 beta | A44131 | 41.344 | 5.17 | 284 | 26 + |
| - Dynactin 2 - Protein disulfide isomerase, thioredoxin related | NP_081427  NP_082235 | 54.367 | 5.64 | 402  445 | 13  10 |
| - Chaperonin^#^ subunit 6a (zeta) - Pyruvate kinase 3^#^ | NP_033968  NP_035229 | 62.962 | 7.28 | 531  531 | 12  6 |
| - Thioredoxin-like protein p46 (Endoplasmic reticulum protein ERp46) - Heterogeneous nuclear ribonucleoprotein F - Actin, beta, gamma - protein kinase, cAMP dependent regulatory, type I, alpha - nuclease sensitive element binding protein 1 | AAH46789  AAH33483  NP_033739  NP_031419  NP_068680  NP_035862 | 49.105 | 5.56 | 417  415  375  375  381  321 | 10  8  6  2  2 |
| - prolyl 4-hydroxylase, beta polypeptide, protein disulfide-isomerase (see also spot #58) - calreticulin - TFG protein   1-peptide proteins omitted | NP_035162  NP_031617  AAD11812 | 69.110 | 5.22 | 509  416  397 | 14  6  5 |
| - Myosin regulatory light chain 2, smooth muscle isoform | Q9CQ19 | 18.396 | 5.34 | 172 | 9 x4 |
| - Plasminogen activator inhibitor 1 RNA-binding protein (PAI1 RNA-binding protein 1) (PAI-RBP1). - 3-oxoacid CoA transferase 1 | Q9CY58  NP_077150 | 56.971 | 7.51 | 407  520 | 3  3 |
| - ATP synthase, H+ transporting, mitochondrial F1 complex, alpha subunit, isoform 1 - Txndc7 protein (thioredoxin)/ protein disulfide isomerase | NP_031531  AAH06865/  NP_082235 | 50.961 | 8.06 | 553  440/  445 | 15  4/4 |
| - protein kinase C substrate 80K-H - ubiquitin specific protease^#^ 5 (isopeptidase T) - Ran-binding protein 3 - calpastatin | NP_032951  NP_038728  NP_082209  NP_033947 | 108.443 | 5.38 | 521  858  593  754 | 6  5  3  3 |
| - calreticulin (see also spot #47) - prolyl 4-hydroxylase, beta polypeptide, protein disulfide-isomerase (see also spot #58, 47) - brain abundant, membrane attached signal protein 1 - dnaK-type molecular chaperone hsp70 | NP_031617  NP_035162  NP_081671  JH0095 | 70.208 | 5.04 | 416  509  226  642 | 10  3  5  2 |
| - UDP-glucose pyrophosphorylase 2 | NP_647458 | 55.800 | 7.34 | 508 | 2 |
| - pyruvate kinase 3^#^ - chaperonin^#^ subunit 6a (zeta) | NP_035229  NP_033968 | 57 | 7.74 | 531  531 | 49  2 |
| - ribosomal protein L3 | NP_038790 | 48 | 7.89 | 403 | 18 |
| - annexin I | NP_034860 | 40 | 6.72 | 317 | 9 |

**Arginylation increased 2-3 fold**

| **Highly abundant** | |  | |  |  |  |  |
| --- | --- | --- | --- | --- | --- | --- | --- |
| - dnaK-type molecular chaperone grp78 precursor | A37048 | |  | |  | 655 | 23 |
| - prolyl 4-hydroxylase, beta polypeptide - Heterogeneous nuclear ribonucleoprotein K - Calreticulin | NP_035162  P61979  AAH03453 | |  | |  | 509  463  416 | 4  3  2 |
| - Vimentin^#^ - prolyl 4-hydroxylase, beta polypeptide | CAA39807  NP_035162 | |  | |  | 466  509 | 74  3 |
| - Actin, alpha/beta/gamma - prolyl 4-hydroxylase, beta polypeptide - serine (or cysteine) proteinase inhibitor, clade B, member 6a | AAH21796  NP_035162  NP_033280 | |  | |  | 375  509  378 | 28  3  3 |
| - Transitional endoplasmic reticulum ATPase (TER ATPase) (15S Mg(2+)-ATPase p97 subunit) (Valosin-containing protein) (VCP) - Endoplasmin precursor (Endoplasmic reticulum protein 99) (94 kDa glucose-regulated protein) (GRP94) (ERP99) (Polymorphic tumor rejection antigen 1) (Tumor rejection antigen gp96). | Q01853  P08113 | | 107,477 | | 5.98 | 806  802 | 26  20 |
| - Reticulocalbin 2 | NP_036122 | | 58,769 | | 4.80 | 319 | 14 |
| - ATP synthase, H+ transporting, mitochondrial F1 complex, alpha subunit, isoform 1 - glutamate dehydrogenase 1 - TPA: embryonic type II keratin 1; K1-emb - protein disulfide isomerase-associated 6 | NP_031531  NP_032159  DAA05633  NP_082235 | | 53,343 | | 7.23 | 553  558  572  445 | 24  8  7  6 |
| - ATP synthase, H+ transporting mitochondrial F1 complex, beta subunit - tubulin, beta^#^ - vimentin^#^ - protein kinase, cAMP dependent regulatory, type II beta | NP_058054  NP_035785  CAA39807  NP_035288 | | 55,312 | | 5.45 | 529  444  466  416 | 44  20  12  2 |
| - ATP synthase, H+ transporting, mitochondrial F1 complex, alpha subunit, isoform 1 - fascin homolog 1, actin bundling protein - protein disulfide isomerase-associated 6 | NP_031531  NP_032010  NP_082235 | | 52,056 | | 7.12 | 553  493  445 | 25  9  6 |
| - ATP synthase, H+ transporting mitochondrial F1 complex, beta subunit - protein disulfide isomerase-associated 6 - tubulin, beta^#^ - vimentin^#^ | NP_058054  NP_082235  NP_035785  CAA39807 | | 52,073 | | 5.47 | 529  445  444  466 | 56  13  7  4 |
| - proteasome (prosome, macropain) 26S subunit, non-ATPase, 11 - Keratin, type I cytoskeletal 14 (Cytokeratin 14) (K14) (CK 14). - actin, gamma, cytoplasmic 1 | CAI24754  Q61781  NP_033739 | | 42,797 | | 6.41 | 422  484  375 | 24  6  2 |
| - serine (or cysteine) proteinase inhibitor, clade B, member 6a - actin, gamma, cytoplasmic 1 - SH3-domain GRB2-like 1, endophilin | NP_033280  NP_033739  NP_038692 | | 45,522 | | 6.01 | 378  375  368 | 17  12  5 |
| - actin, gamma, cytoplasmic 1 - 40S ribosomal protein SA (p40) (34/67 kDa laminin receptor). - serine (or cysteine) proteinase inhibitor, clade B, member 6a - similar to protein phosphatase 2A inhibitor-2 I-2PP2A | NP_033739  P14206  NP_033280  XP_354669 | | 41,507 | | 6.10 | 375  295  378  222 | 13+6  3  2  2 |
| - COP9 signalosome subunit 4 - actin, gamma, cytoplasmic 1 - SH3-domain GRB2-like B1 (endophilin) - proteasome 26S non-ATPase subunit 13 - keratinocyte associated protein 1 - RIKEN cDNA 2410018C20 | NP_036131  NP_033739  NP_062337  NP_036005  NP_579935  NP_080699 | | 41,236 | | 5.87 | 406  375  365  376  551  369 | 15  5  5  3  3  2 |
| - Poly(rC)-binding protein 3 (Alpha-CP3) - ubiquitin fusion-degradation 1 like protein; UFD1p | P57722  AAD08719 | | 39,148 | | 5.87 | 339  307 | 3  2 |
| - LIM and SH3 protein 1^#^ - Acidic ribosomal phosphoprotein P0 | NP_034818  AAH11106 | | 36,597 | | 7.10 | 263  317 | 7  6 |
| - tropomyosin 1, alpha - Proliferating cell nuclear antigen (PCNA) (Cyclin) | NP_077745  P17918 | | 36,933 | | 5.25 | 284  261 | 33  3 |
| - annexin A3 | NP_038498 | | 34,562 | | 5.84 | 323 | 24 |
| - Ferritin light chain 1; glutamate receptor 7 - ATP synthase, H+ transporting, mitochondrial F0 complex, subunit d (UNLIKELY) –left off the rest of minor proteins | AAH85309; XP_131647  AAH81431 | | 22,463 | | 5.89 | 183; 1489  161 | 13+5  3 |
| - myosin regulatory light chain-like | NP_080340 | | 18,896 | | 5.21 | 172 | 25 |
| - ATP synthase, H+ transporting, mitochondrial F1 complex, alpha subunit, isoform 1 - protein disulfide isomerase-associated 6 - glutamate dehydrogenase 1 | NP_031531  NP_082235  NP_032159 | | 53,109 | | 7.36 | 553  445  558 | 14  5  4 |
| - purine-nucleoside phosphorylase - Proteasome (prosome, macropain)^#^ 28 subunit, alpha - Ribosomal protein L7a | NP_038660  AAH88981  AAH84678 | | 30,249 | | 6.00 | 289  249  266 | 18  3  2 |
| - RAB1, member RAS oncogene family - Prefoldin subunit 3 (Von Hippel-Lindau binding protein 1) (VHL binding protein-1) (VBP-1). - Ran-specific GTPase-activating protein (Ran binding protein 1) (RANBP1) (HpaII tiny fragments locus 9a protein). | NP_033022  P61759  P34022 | | 26,674 | | 5.78 | 205  185  203 | 5+4+3  3  2 |
| - DEAD/H (Asp-Glu-Ala-Asp/His) box polypeptide 3, X-linked - Transferrin | NP_034158  NP_598738 | | 79,005 | | 7.24 | 662  697 | 9  2 |
| - tropomyosin beta 2 | A44131 | | 40,569 | | 5.16 | 284 | 30 |
| - enolase 1, alpha non-neuron - endoplasmic reticulum protein ERp29 precursor - 14-3-3 protein zeta/delta (Protein kinase C inhibitor protein-1) (KCIP-1) (SEZ-2); tyrosine 3-monooxygenase/tryptophan 5-monooxygenase activation protein | NP_075608  NP_080405  P63101  NP_035870 | | 29,980 | | 6.30 | 434  262  245 | 6  6  3 |
| - 14-3-3 protein subunits zeta, beta, tau, eta; tyrosine 3-monooxygenase/tryptophan 5-monooxygenase activation protein | JC5384 | | 29,547 | | 5.55 | 245 | 9+8+7+5+5+3 |
| - contains human keratin and bovine albumin |  | |  | |  |  |  |
| - EF hand domain containing 2 - chloride intracellular channel 1 - tyrosine 3-monooxygenase\ /tryptophan 5-monooxygenase activation protein, epsilon polypeptide; 14-3-3 epsilon - actin, gamma, cytoplasmic 1 - RIKEN cDNA 2010208K18 - preprocathepsin B - tumor protein D52-like 2 - 14-3-3 protein zeta/delta (Protein kinase C inhibitor protein-1) (KCIP-1) (SEZ-2); tyrosine 3-monooxygenase/tryptophan 5-monooxygenase activation protein - ribosomal protein S3 | NP_080270  NP_254279  CAI26030  NP_033739  NP_082371  AAA37494  NP_079758  P63101  NP_035870  NP_036182 | | 31,884 | | 5.50 | 240  241  255  375  244  339  220  245  243 | 9  7  7  6  4  4  3  2  2 |
| - Calpain small subunit 1 (CSS1) Calcium-dependent protease small subunit 1) (Calcium-dependent protease small subunit) (CDPS) (Calpain regulatory subunit) Calcium-activated neutral proteinase small subunit) (CANP small subunit). - heat shock protein^#^ HSP27 | O88456  AAA18336 | | 27,922 | | 5.62 | 269  197 | 5  2+2 |

**^#^**proteins from the Supplemental Table 3 that also appear in Table 1 in the main text
