# Supplementary material for: Global Analysis of Posttranslational Protein Arginylation
Source: PLoS Biol. 2007 Sep 25;5(10):e258. doi: 10.1371/journal.pbio.0050258 (PMC1988855; doi:10.1371/journal.pbio.0050258)
Supplement: Supplementary file 8 [file pbio.0050258.st004.doc]

**Supplemental Table 4. 2D gel protein spot comparison between wild type cells (WT), wild type cells treated with proteasome inhibitor (WTPI), and Ate1-/- cells. See text and Fig. 1 for other details.**

| **Type** | **Description** | **# of spots** |
| --- | --- | --- |
| Proteasome dependent N-end rule degradation | Up- in WTPI and Ate1-/- compared to WT | 83 |
| Proteasome independent up-regulated in Ate1-/- | Up in Ate1-/- compared to WT | 208 |
| Secondary proteasome dependent up-regulation in Ate1-/- | Up in Ate1-/- compared to WT, down in WTPI compared to Ate1-/- and WT | 19 |
| Proteasome independent down-regulated in Ate1-/- | Down in Ate1-/- compared to WT | 313 |
| Secondary proteasome dependent down regulation in Ate1-/- I | Down in Ate1-/-, up in WTPI | 19 |
| Secondary proteasome dependent down regulation in Ate1-/- II | Down in WTPI, down in Ate1-/- | 91 |
| Ate1- independent proteasome degradation | up- in WTPI compared to WT and Ate1-/- | 99 |
| ___” ___  “ | up in WTPI compared to WT, no change in Ate1-/- | 43 |
| Ate1- independent proteasome dependent secondary regulation | Up- in WT and sometimes Ate1-/- compared to WTPI | 155 |

Totals:

- 1030 different altered spots between the three gels
- 297 altered in proteasome-dependent Ate1-independent way (up- or down)
- 733 altered in Ate1-dependent way

Wild type:

- 313 up-regulated proteasome independent
- 110 up-regulated with secondary proteasome-dependent effects

Ate1-/-:

- 208 up-regulated proteasome-independent
- 83 up-regulated proteasome-dependent – N-end rule
- 19 up-regulated with secondary proteasome-dependent effects
